# Supplementary material for: PTEN Regulates PI(3,4)P2 Signaling Downstream of Class I PI3K
Source: Mol Cell. 2017 Nov 2;68(3):566–580.e10. doi: 10.1016/j.molcel.2017.09.024 (PMC5678281; doi:10.1016/j.molcel.2017.09.024)
Supplement: Document S1. Figures S1–S7 [file mmc1.pdf]

Molecular Cell, Volume *68*

Supplemental Information

PTEN Regulates PI(3,4)P_2 Signaling

Downstream of Class I PI3K

Mouhannad Malek, Anna Kielkowska, Tamara Chessa, Karen E. Anderson, David Barneda, Pınar Pir, Hiroki Nakanishi, Satoshi Eguchi, Atsushi Koizumi, Junko Sasaki, Véronique Juvin, Vladimir Y. Kiselev, Izabella Niewczas, Alexander Gray, Alexandre Valayer, Dominik Spensberger, Marine Imbert, Sergio Felisbino, Tomonori Habuchi, Soren Beinke, Sabina Cosulich, Nicolas Le Novère, Takehiko Sasaki, Jonathan Clark, Phillip T. Hawkins, and Len R. Stephens

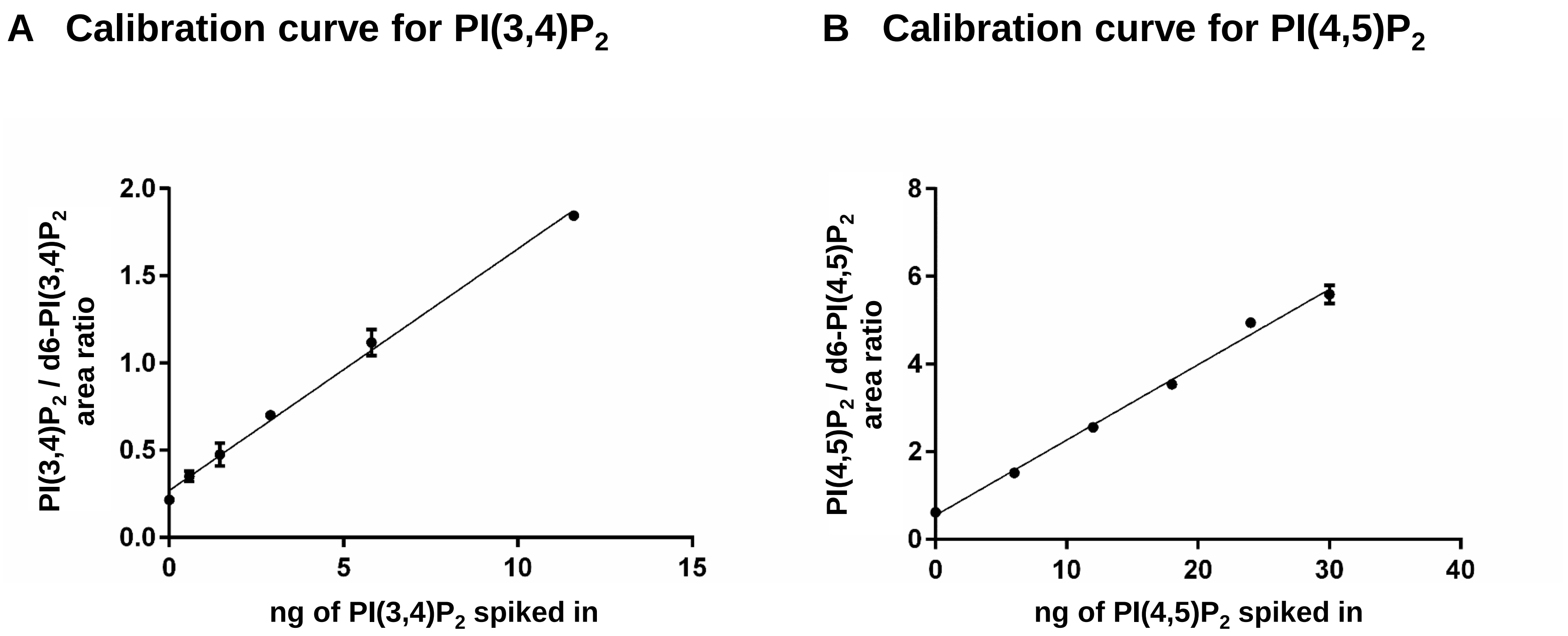

Figure S1. A HPLC-MS method for measuring PI(3,4)P_2 and PI(4,5)P_2. Related to Figure 1.
(A) Calibration curve for PI(3,4)P_2. **(B**) Calibration curve for PI(4,5)P_2. **Methods:** Mcf10a cells were incubated with 100 µM rotenone and 100 µM CCCP decoupler for 24 hrs to reduce endogenous levels of polyphosphoinositides. Cells were then killed in 1.0 M HCL and processed to form an initial homogenous lipid extraction phase, as described in Methods. The indicated quantities of synthetic C38:4 PI(3,4)P_2 or PI(4,5)P_2 and a fixed quantity of d6-C38:4 PI(3,4)P_2 and PI(4,5)P_2 were then added to portions of this extraction phase and processed by HPLC-MS. The data shown are for means +/- SD of three technical replicates.

A PI(3,4,5)P_3 levels in Mcf10a cells with altered expression of phosphatases

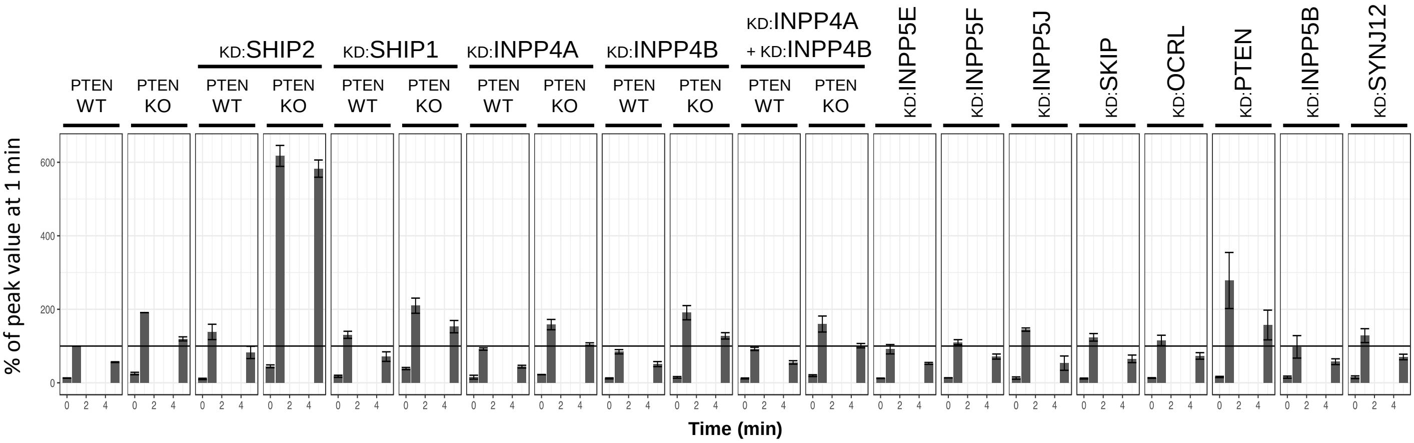

B PIP levels in Mcf10a cells with altered expression of phosphatases

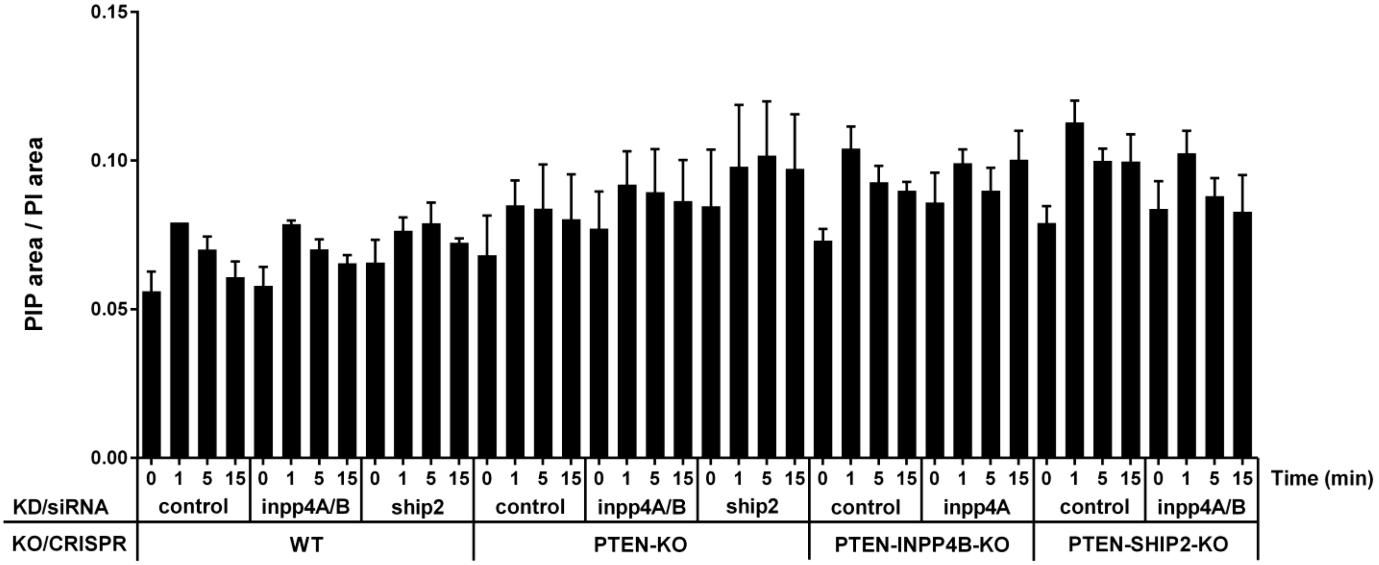

C Phosphorylation of the EGFR in genetically modified Mcf10a cells

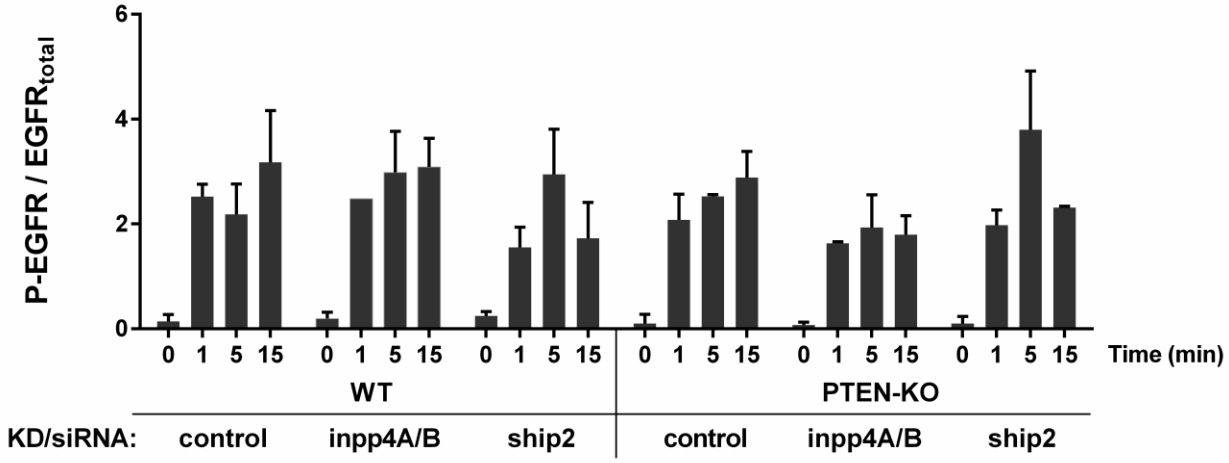

Figure S2. Characterisation of genetically modified Mcf10a cells. Related to Figure 2.

Figure S2. Characterisation of genetically modified Mcf10a cells. Related to Figure 2.

(A) $PI(3,4,5)P_3$ levels in WT or $PTEN^{-/-}$ (PTEN-KO) Mcf10a cells treated with the indicated siRNAs, starved and then stimulated with EGF (10ng/ml) for 0, 1 or 5 min. Phosphoinositides were analysed by HPLC-MS using a C4 column and $PI(3,4,5)P_3$/PI ratios calculated, as described in the Methods. For each population of cells, the data shown are normalised to the value for 1 min EGF. Data are means +/- SD of three independent experiments.

(B) PIP levels in WT or $PTEN^{-/-}$ (PTEN-KO) Mcf10a cells treated with the indicated siRNAs, starved and then stimulated with EGF (10ng/ml) for 0, 1, 5 or 15 min. Phosphoinositides were analysed by HPLC-MS using a C4 column and PIP/PI ratios calculated, as described in the Methods. Data represent means ± SD of 3 biological replicates (for siRNA suppression in WT or PTEN-KO cells) or 3 technical replicates (for PTEN-INPP4B-KO or PTEN-SHIP2-KO cells).

(C) The phosphorylation of the EGFR in Mcf10a cells treated with the indicated siRNAs, starved and then stimulated for the indicated times with EGF. The levels of EGFR and phospho-EGFR were quantified by Western blot and LiCor and represent means +/- SD of three independent experiments.

A Expression of phosphatases in cell populations treated with siRNA

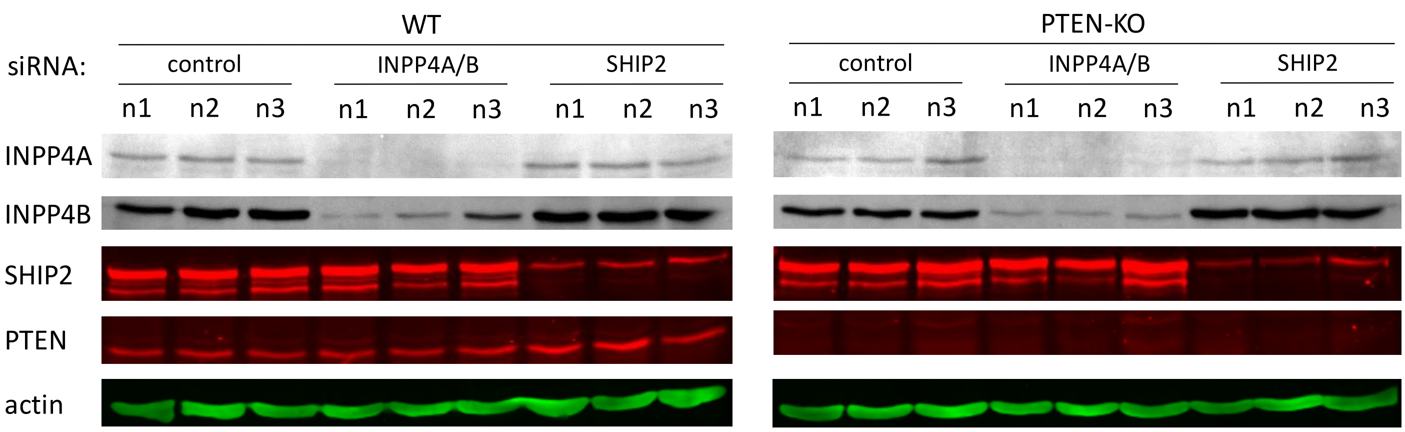

B Expression of phosphatases in clones derived by CRISPR/Cas9 gene editing

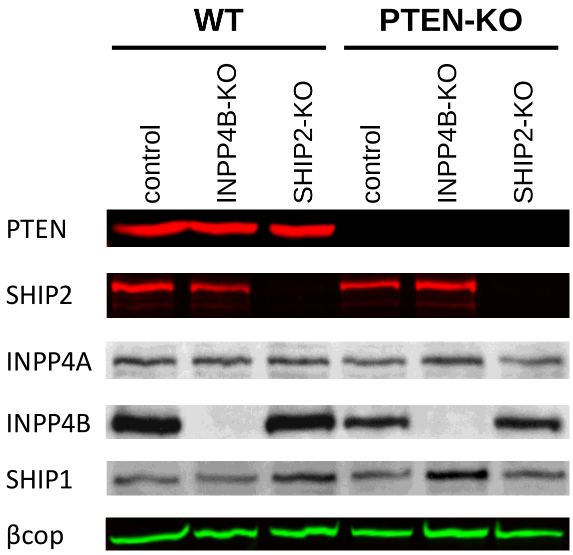

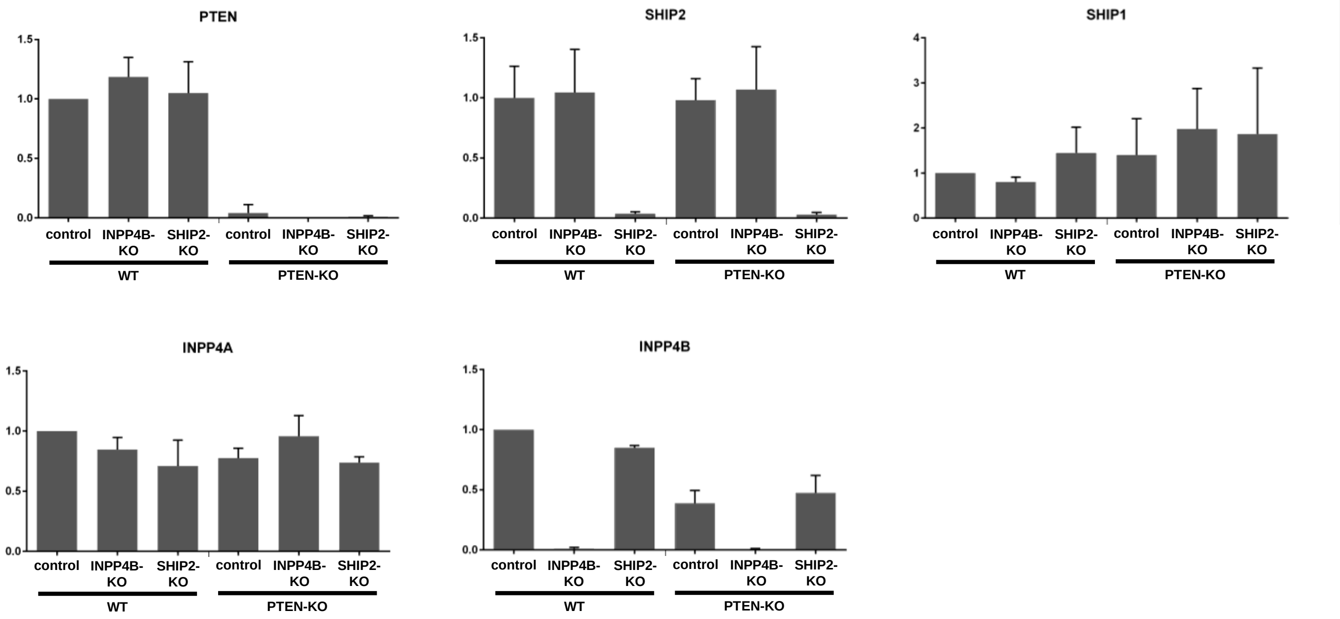

Figure S3. Expression of phosphatases in genetically modified Mcf10a cells. Related to Figure 2.

Expression of phosphatases in Mcf10a cell clones derived by siRNA gene suppression (**A**) or CRISPR/Cas9 gene editing (**B**). A representative Western blot is shown, together with the results of quantifying analogous blots using LiCor or Aida software; data are for expression levels of the indicated protein normalised to WT and are means +/- SD of three independent experiments.

A The effect of PI-103 on the accumulation of PI(3,4,5)P_3 and PI(3,4)P_2 in EGF-stimulated [INPP4A/B-KD, PTEN-KO] Mcf10a cells

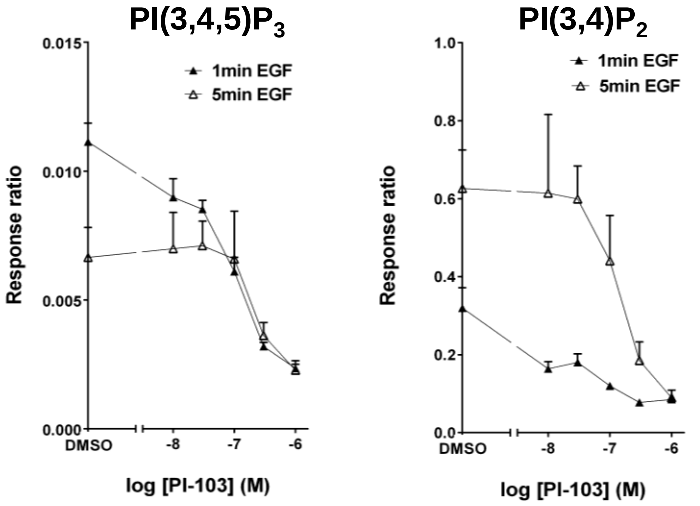

B The effect of BYL-719 on the accumulation of PI(3,4,5)P_3 and PI(3,4)P_2 in EGF-stimulated [PTEN-INPP4B-KO] Mcf10a cells

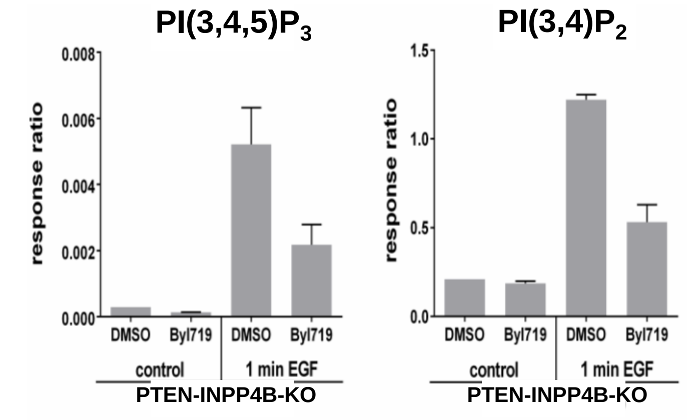

C The effect of siRNA-directed suppression of Class II PI3Kα and β on the accumulation of PI(3,4,5)P_3 and PI(3,4)P_2 in EGF-stimulated [INPP4A/B-KD, PTEN-KO] Mcf10a cells

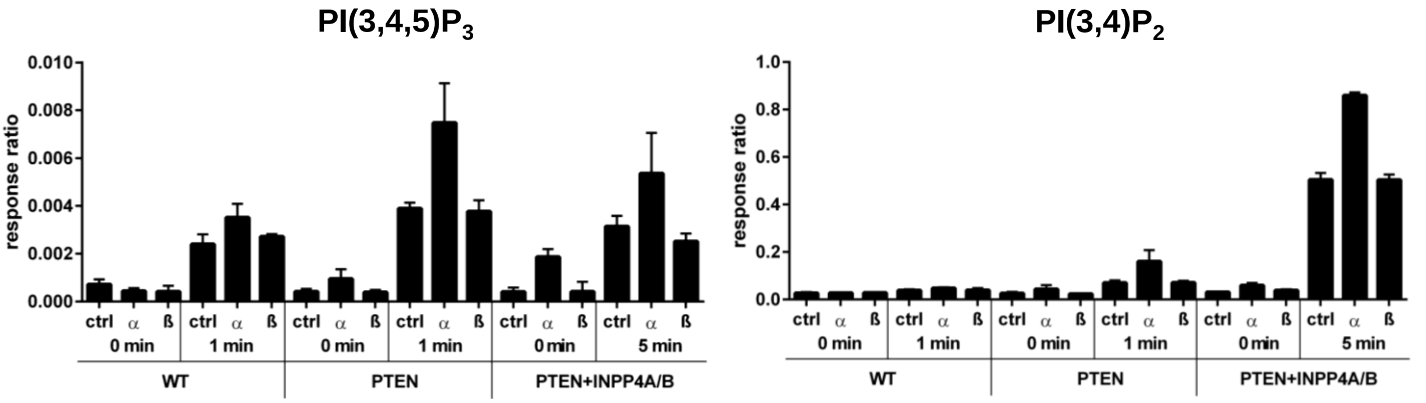

D siRNA-directed suppression of Class II PI3Kα and β in Mcf10a cells

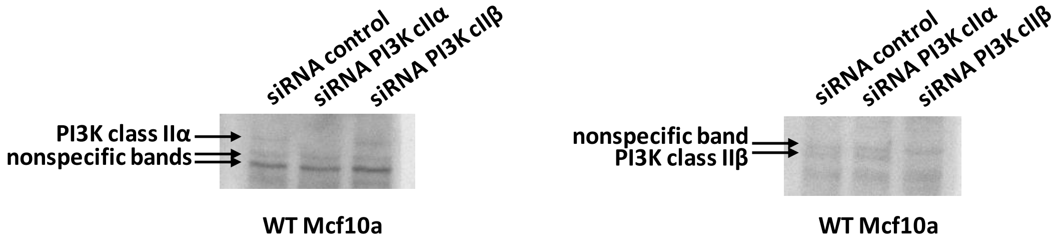

E The effect of reduced 5-phosphatase expression on EGF-stimulated PI(3,4,5)P_3 and PI(3,4)P_2 accumulation in EGF-stimulated Mcf10a cells

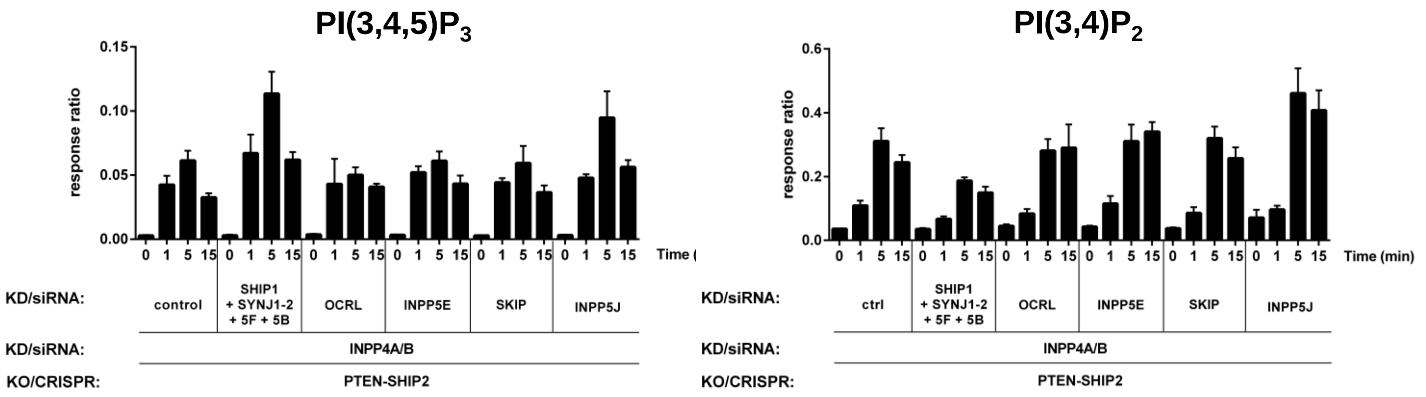

Figure S4. Evidence for Class I PI3K-driven accumulation of PI(3,4)P_2 in EGF-stimulated Mcf10a cells. Related to Figure 4.

(A) PI(3,4,5)P_3 and PI(3,4)P_2 measurements in Mcf10a cells starved, treated with the indicated concentration of PI-103 for 20 min and then stimulated with EGF (10ng/ml) for 1 min or 5 min. Data are means +/- SD of 3 technical replicates.

(B) PI(3,4,5)P_3 and PI(3,4)P_2 measurements in [PTEN-INPP4B-KO] cells starved, treated with 2μM BYL-719 or vehicle for 10 min and then stimulated with EGF (10ng/ml) or vehicle for 1 min. Data are means +/- SD of three biological replicates.

(C) PI(3,4,5)P_3 and PI(3,4)P_2 measurements in WT, PTEN-KO and [INPP4A/B-KD, PTEN-KO] cells incubated with the indicated siRNAs directed against Class II PI3Kα or β, starved, and then stimulated with EGF (10ng/ml) for 5 min. Representative data of 1 of 3 biological replicates is shown, as means +/- SD of 3 technical replicates.

(D) Representative Western blots showing expression of Class II PI3Kα or β in analogous experiments to those shown in Panel (C).

(E) PI(3,4,5)P3 and PI(3,4)P2 measurements in PTEN-SHIP2-KO Mcf10a cells treated with the indicated siRNAs and then starved and stimulated with EGF (10 ng/ml) for 0, 1, 5 or 15 min. Data are means +/- SD of 3 technical replicates.

A Comparison between HPLC-MS and ^{33}P-labelling: example HPLC traces

HPLC-MS on C18-column **HPLC on anion exchange column**

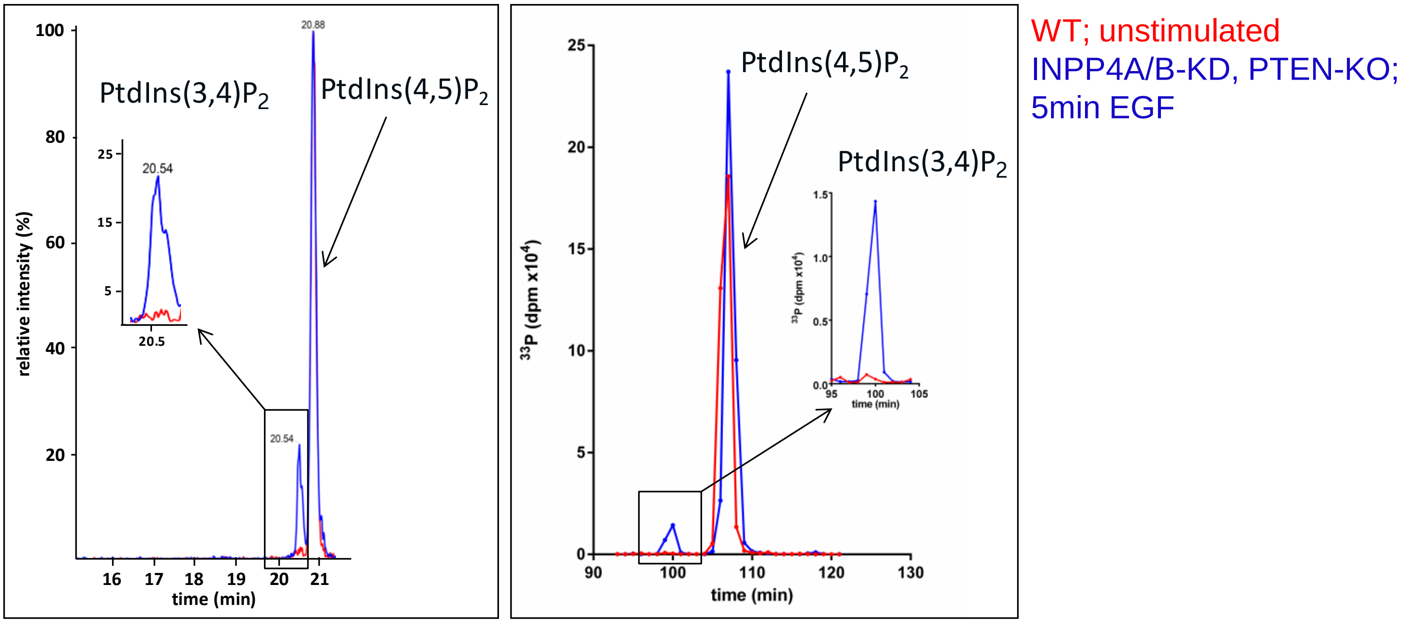

B Comparison between HPLC-MS and ^{33}P-labelling

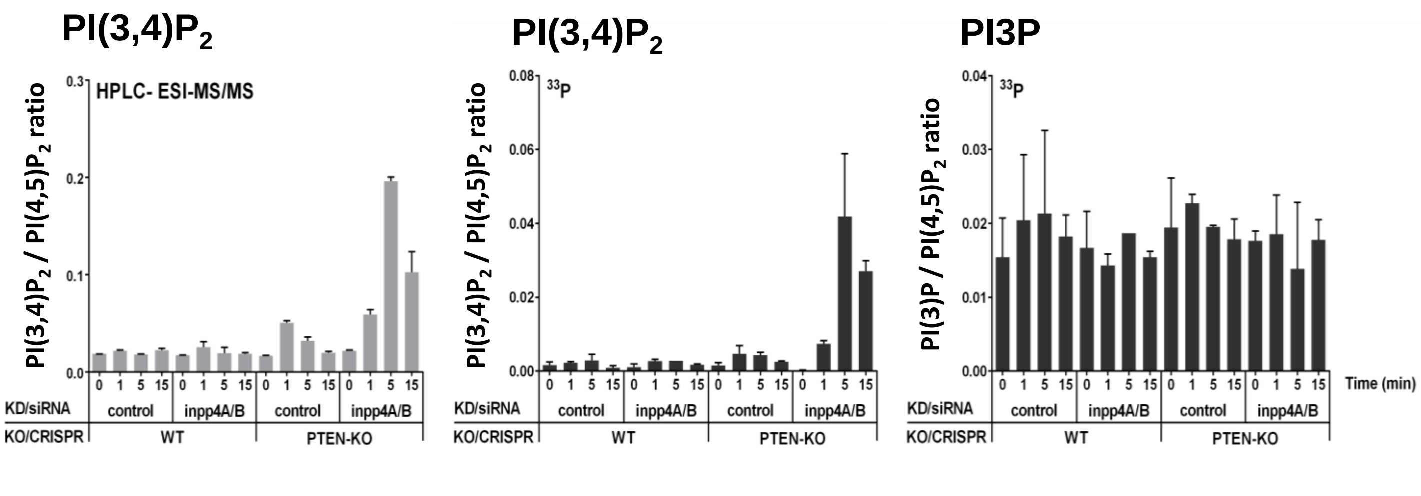

C Comparison between MS methods

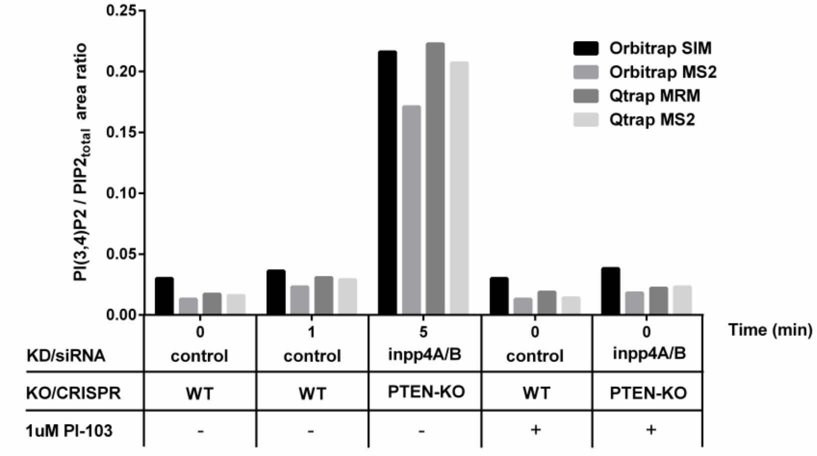

Figure S5. Comparison between different methods used to measure PI(3,4)P_2. Related to Figure 4.

(A) Example HPLC traces illustrating the raw data derived from the analysis of parallel samples of unlabelled or [^{33}-P]Pi-labelled, WT or [INPP4A/B-KD, PTEN-KO] Mcf10a cells. Cells were starved and then left unstimulated or stimulated with EGF (10ng/ml) for 5 min and analysed by HPLC-MS (left panel) or via deacylation and anion-exchange chromatography (right panel); see methods.

(B) Quantitation of traces analogous to those shown in Panel A. WT or PTEN-KO cells treated with the indicated siRNA were starved and then stimulated with EGF for 0, 1, 5 or 15 min. For the HPLC-MS values, peak areas derived from molecules corresponding to C38:4 PI(3,4)P_2 and PI(4,5)P_2 were first corrected for the recovery of internal deuterated standards before calculating a PI(3,4)P_2/PI(4,5)P_2 ratio. For the ^{33}P-labelling values, HPLC fractions corresponding to the appropriate glycerophosphoinositide species were summed, background radioactivity subtracted, and then a simple PI(3,4)P_2/PI(4,5)P_2 or PI3P/PI(4,5)P_2 ratio derived. The data shown are means +/- SD of two biological replicates.

(C) A comparison of the values obtained for PI(3,4)P_2 using identical HPLC chromatography conditions but different fragmentation strategies. WT or PTEN-KO Mcf10a cells were incubated with the indicated siRNAs, starved, treated with or without PI-103 (1 µM) for 20 min and then stimulated with EGF (10ng/ml) for 0, 1 or 5 min. Levels of PI(3,4)P_2 were measured using triple quadrupole mass spectrometer (AB SCIEX 4000 QTrap) and fragment ions were detected in multiple reaction monitoring (MRM) or MS2 mode. Parallel measurements were performed on an orbitrap mass spectrometer (Thermo Scientific Orbitrap Elite), where ions' m/z was measured with high accuracy using single ion monitoring (SIM) or MS2 approach. The values we obtained using alternative fragmentation strategies for our MS analyses reported similarly high values for PI(3,4)P_2 in both starved and PI-103-treated cells (C). A comparison between measurements for PI(3,4)P_2 obtained by HPLC-MS or [^{33}P]Pi-radiolabeling in both starved and EGF-stimulated WT, INPP4A/B-KD, PTEN-KO and [INPP4A/B-KD, PTEN-KO] cells generated a remarkably similar pattern of changes in PI(3,4)P_2/PI(4,5)P_2 ratios between conditions, but the absolute values for this ratio were higher, and the relative increases upon EGF-stimulation were lower, for the values generated by mass spectrometry (B). Note: the radiolabelling methodology allowed an estimate of PI3P levels and these are included for comparison to show they were not significantly affected by loss of PTEN (B).

Comparisons between HPLC-MS and [^{33}P]Pi-radiolabeling are difficult, because the radiolabeling approach only measures phosphoinositides that have both incorporated ^{33}P and are susceptible to methylamine-mediated deacylation, whilst our HPLC-MS method only measures intact C38:4 species. However, if the assumption is made that EGF would stimulate a similar increase in PI(3,4)P_2 measured by each method, then it must be considered plausible that contamination from non-PI(3,4)P_2 derived molecules significantly contributes to our HPLC-MS measurements for PI(3,4)P_2 in starved or PI-103-treated cells, and hence leads to an overestimation of the Class I PI3K-insensitive pool predicted by the modelling.

A Identification of PIP derived from PI(3,4)P_2 by Mcf10a cytosol as PI4P

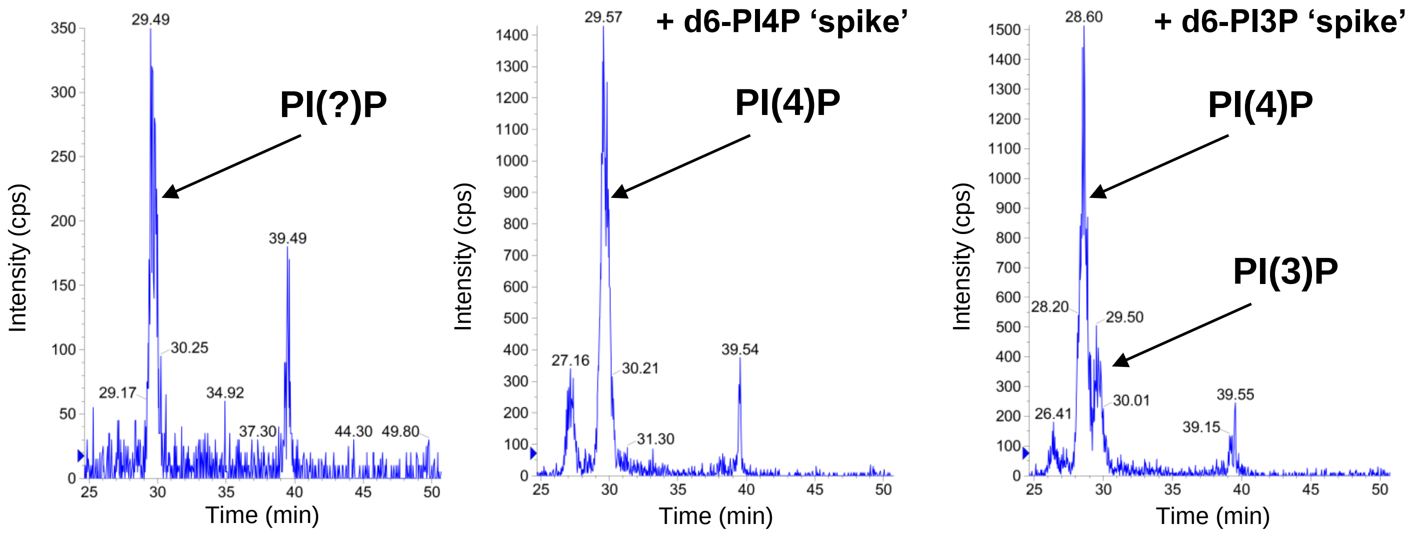

B Recombinant PTEN acts as PI(3,4)P_2 phosphatase in Mcf10a cytosol

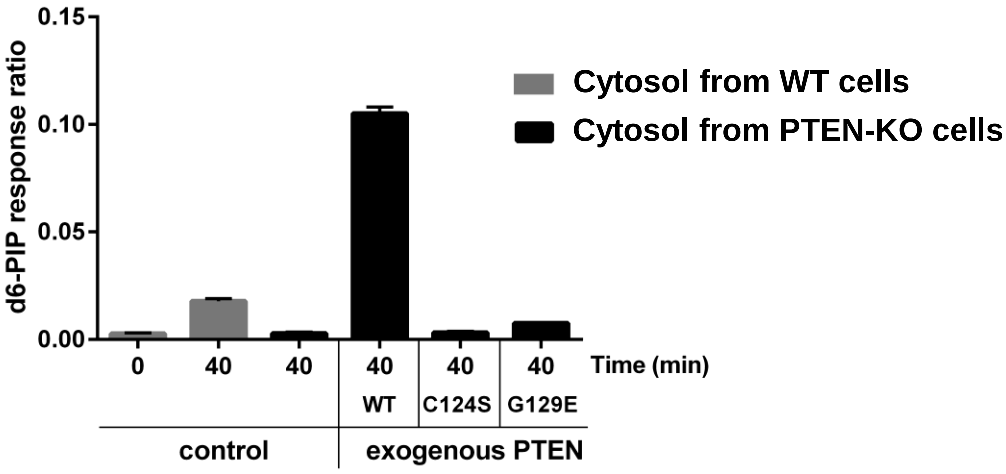

Figure S6. Dephosphorylation of PI(3,4,5)P_3 and PI(3,4)P_2 by Mcf10a cytosol. Related to Figure 5.

(A) HPLC-MS separation of the products of d6-PI(3,4)P_2 dephosphorylation by [INPP4B CRSIPR-KO] Mcf10a cytosol (left panel), together with analogous separations of samples in which additional d6-PI4P (middle) or d6-PI3P (right panel) had been added after reactions were terminated.

(B) The formation of d6-PIP by Mcf10a cytosol incubated with d6-PI(3,4)P_2. Cytosol was prepared from WT or PTEN-KO cells, and supplemented with 30nM recombinant WT or mutant PTEN, as indicated; see Methods for details.

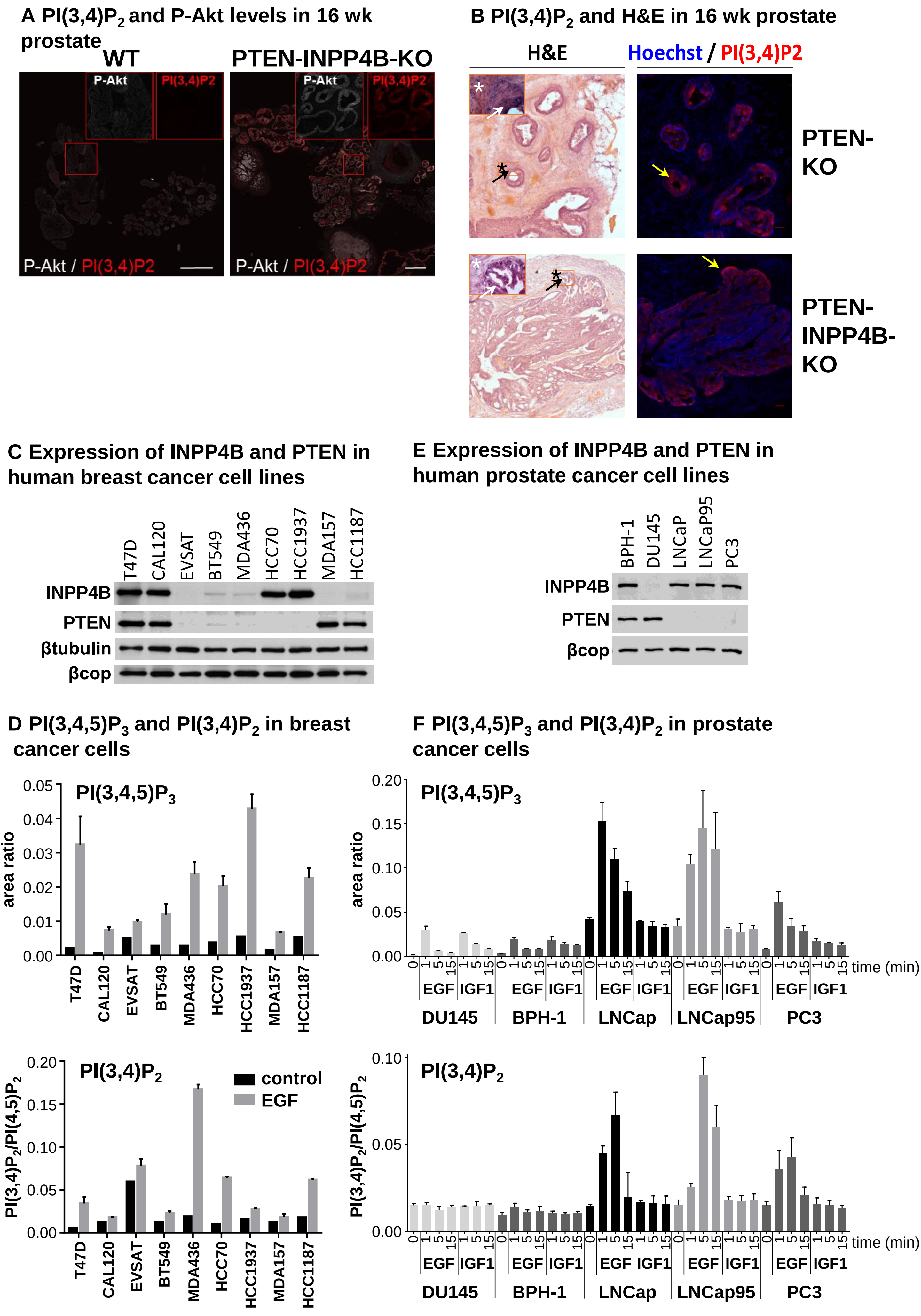

A PI(3,4)P2 and P-Akt levels in 16 wk prostate
WT
PTEN-INPP4B-KO
P-Akt
PI(3,4)P2
P-Akt / PI(3,4)P2
B PI(3,4)P2 and H&E in 16 wk prostate
H&E
Hoechst / PI(3,4)P2
PTEN-KO
PTEN-INPP4B-KO
C Expression of INPP4B and PTEN in human breast cancer cell lines
T47D
CAL120
EVSAT
BT549
MDA436
HCC70
HCC1937
MDA157
HCC1187
INPP4B
PTEN
βtubulin
βcop
E Expression of INPP4B and PTEN in human prostate cancer cell lines
BPH-1
DU145
LNCaP
LNCaP95
PC3
INPP4B
PTEN
βcop
D PI(3,4,5)P3 and PI(3,4)P2 in breast cancer cells
PI(3,4,5)P3
area ratio
0.05
0.04
0.03
0.02
0.01
0.00
T47D
CAL120
EVSAT
BT549
MDA436
HCC70
HCC1937
MDA157
HCC1187
PI(3,4)P2
control
EGF
PI(3,4)P2/PI(4,5)P2
0.20
0.15
0.10
0.05
0.00
T47D
CAL120
EVSAT
BT549
MDA436
HCC70
HCC1937
MDA157
HCC1187
F PI(3,4,5)P3 and PI(3,4)P2 in prostate cancer cells
PI(3,4,5)P3
area ratio
0.20
0.15
0.10
0.05
0.00
0 1 5 15 1 5 15
time (min)
EGF
IGF1
DU145
BPH-1
LNCap
LNCap95
PC3
PI(3,4)P2
PI(3,4)P2/PI(4,5)P2
0.10
0.05
0.00
0 1 5 15 1 5 15
time (min)
EGF
IGF1
DU145
BPH-1
LNCap
LNCap95
PC3

Figure S7. The impact of PTEN on PI(3,4)P_2 levels in mouse prostate and human prostate and breast cancer lines. Related to Figure 7.

(A) An example of anti-phospho-S473-AKT and anti-PI(3,4)P_2 stained sections of prostates taken from PTEN$^{flox/flox}$, PbCre$^{-/-}$ ('WT') and PTEN$^{flox/flox}$, PbCre$^{+/-}$ INPP4B$^{-/-}$ ('PTEN-INPP4B-KO') mice at 16 weeks of age (12μm sections). Some areas in anterior prostate of WT and PTEN-INPP4B-KO wide field images are shown at higher magnification, to show localisation of antibody staining. Scale bar represents 1 mm.

(B) H&E, DAPI and anti-PI(3,4)P_2 stained sections of prostates taken from PTEN$^{flox/flox}$, PbCre$^{+/-}$ ('PTEN-KO') and PTEN$^{flox/flox}$, PbCre$^{+/-}$ INPP4B$^{-/-}$ ('PTEN-INPP4B-KO') mice at 16 weeks of age (scale bar represents 69 μm). In the H&E panel and insets, arrows indicate position of hyperproliferative epithelium and asterisks indicate reactive stroma and loss of smooth muscle cell layer. In the Hoechst/PI(3,4)P_2 panel, yellow arrows indicate high PI(3,4)P_2 levels in the acini that correspond to the insets in the H&E images. The images shown are typical of 3 prostate sections analysed from 3 mice in each genotype. H&E images were stitched manually using Volocity software with brightness correction.

(C) Western blot showing relative expression of INPP4B, PTEN, βtubulin and βcop in the indicated human breast cancer cell lines (20μg total protein loaded per lane).

(D) PI(3,4,5)P_3 and PI(3,4)P_2 levels in human breast cancer cell lines starved and then stimulated with EGF (10ng/ml) for 5 min or left unstimulated. Phosphoinositides were analysed by HPLC-MS using a C4 or C18 column for measurement of PI(3,4,5)P_3 and PI(3,4)P_2, respectively. The value for the ion current area for endogenous PIP_3 was divided by the value for the ion current area of endogenous PIP_2, to correct for cell mass. The values for the ion current areas of PI(3,4P_2 and PI(4,5)P_2 were first corrected for the recovery of the appropriate internal deuterated standards and then presented as an approx molar ratio for PI(3,4P_2 divided by PI(4,5)P_2 (for details refer to the Methods section). Data are means +/- range of two biological replicates (EGF) or a single biological replicate (control).

(E) Western blot showing relative expression of INPP4B, PTEN and βcop in the indicated human prostate cancer cell lines (20μg total protein loaded per lane).

(F) PI(3,4,5)P_3 and PI(3,4)P_2 levels in human prostate cancer cell lines starved and then stimulated with EGF (10ng/ml) of IGF1 (50ng/ml) for 0, 1, 5 or 15 min. Phosphoinositides were analysed by HPLC-MS using a C4 or C18 column for measurement of PI(3,4,5)P_3 and PI(3,4)P_2, respectively. Data are presented as described for Panel (D) and are means +/- SD of at least two independent experiments.
