# Supplementary material for: PTEN Regulates PI(3,4)P2 Signaling Downstream of Class I PI3K
Source: Mol Cell. 2017 Nov 2;68(3):566–580.e10. doi: 10.1016/j.molcel.2017.09.024 (PMC5678281; doi:10.1016/j.molcel.2017.09.024)
Supplement: Data S3. Mathematical Model Description [file mmc4.pdf]

Computational model of phosphatase activities on PI(3,4,5)P3 and PI(3,4)P2

The model was developed using the software COPASI, version 4.19 (Hoops et al, 2006).

List of Reactions

All reactions are irreversible.
k_{A1} is fixed at 1, all other kinetic constants were estimated using the experimental measurements.

Name	Reaction, Modifier	Rate Law
EGF binding (lumped reaction)	2 EGFR + 2 EGF -> EGFR-P	Linear tetramolecular
EGFR internalization	EGFR-P -> Ø	Linear unimolecular
PI(4,5)P2 to PI(3,4,5)P3 by PI3K	PI(4,5)P2 -> PI(3,4,5)P3; PI3Keff1	Linear unimolecular
PI(3,4,5)P3 to PI(4,5)P2 by PTEN	PI(3,4,5)P3 -> PI(4,5)P2; PTEN	Linear unimolecular
PI(3,4,5)P3 to PI(3,4)P2 by SHIP2	PI(3,4,5)P3 -> PI(3,4)P2; SHIP2	Linear unimolecular
PI(3,4,5)P3 to PI(3)P by INPP4	PI(3,4)P2 -> PI(3)P; INPP4A/B	Linear unimolecular
PI(3,4)P2 to PI4 by PI(4)P	PI(3,4)P2 -> PI(4)P; PTEN	Linear unimolecular
PI(3,4,5)P3 to PI(3,4)P2 by X	PI(3,4,5)P3 -> PI(3,4)P2; X	Linear unimolecular
PI-103 diffusion	PI-103_ext -> PI-103_int; mode_PI-103_diff	Linear unimolecular
PI(3,4)P2 to PI(Y)P by Y	PI(3,4)P2-> PI(Y)P; Y	Linear unimolecular

List of Assignments

1. PI3Keff1 is the fraction of activated PI3K calculated by the following algebraic equation:

$$PI3Keff1 = \frac{[EGFR - P]}{K_{PI3KAct} + [EGFR - P]} \cdot \frac{K_{PI-103}}{K_{PI-103} + [PI - 103]}$$

$K_{PI3KAct}$ was estimated. $K_{PI\text{-}103}$ was fixed at 0.001.

2. PI(3,4)P2_BG: The background concentration of PI(3,4)P2 in the cells, this pool is not accessible to the phosphatases and kinases, therefore has a fixed value which is estimated from all experiments.

The total concentration of PI(3,4)P2 in the cells, P(3,4)P2_total, is calculated as:

PI(3,4)P2_total = PI(3,4)P2 + PI(3,4)P2_BG

During parameter estimation procedures, the experimental measurements are mapped to the total concentrations of the model.

An identical approach was used to tackle concentrations of PI(3,4,5)P3.

Implementation of the enzyme manipulations in the model

The variable "mode_E" indicates the mode of E where E is an enzyme. The value of the variable mode_E is 1 to simulate a wild type situation (default) and 0 to simulate silenced/knocked-out mutants. More complex codes were used to discriminate between siRNA and CRISPR experiment (not shown). The value of the mode is used to decide on the fraction of active enzyme. In the current model, silencing of SHIP2 and INPP4 are considered total, while silencing of 'X' is partial since we only used the data corresponding to the silencing of some 5-phosphatases.

The same procedure applies for inhibitor diffusion reaction, the mode_PI-103 variable allowing or blocking the reaction transporting the PI3K inhibitor in the cell.

Implementation of Activation and Inhibition Events

Two discrete events are used to represent the stimulation of phophoinositide signalling by EGF and the inhibition of the signal by PI-103.

The activation event is triggered 300 sec after start of the experiment in the current model. The signal is fired by setting the concentration of EGF to 0.00157 uM.

The inhibition event is triggered 365 sec after the start of the experiment (only when mode_PI-103 = 1).

List of Parameters and Predicted Values

Parameter	Value of best fit	StDev of 15 best fits
(PI(3,4,5)P3 to PI(4,5)P2 by PTEN).kmax:	0.133003	0.025504077
(PI(3,4,5)P3 to PI(3,4)P2 by SHIP2).kmax:	0.11987	0.011574055
(PI(3,4,5)P3 to PI(3)P by INPP4).kmax:	0.0687371	0.00886913
(PI(3,4)P2 to PI4 by PTEN).kmax:	0.0968116	0.078980283
(PI(3,4,5)P3 to PI(3,4)P2 by X).kmax:	0.0447312	0.002821947
(PI(3,4)P2 to PI(Y)P by Y).kmax:	0.00624926	0.000634545
(PI-103 diffusion).kmax:	5.56E-05	7.59458E-06
Values[partial_X].InitialValue:	0.500856	0.019293653
Values[P(3,4)P2_BG].InitialValue:	0.741007	0.035144284
Values[P(3,4,5)P3_BG].InitialValue:	0.0501727	0.010940671
Values[K_PI3KAct].InitialValue:	0.65605	0.271068568
(EGF binding).k1:	620.385	56.92199762
(EGFR internalization).k1:	0.13297	0.11854518

Parameter Estimation

The estimation of parameters was performed using COPASI and SBpipe (Dalle Pezze, Le Novère, 2017) using the Genetic Algorithm with Stochastic Ranking (Runarsson, Yao, 2000), with 400 generations and a population size of 400, all other settings were kept at their default values.

References

Dalle Pezze P, Le Novère N, 2017. SBpipe: a collection of pipelines for automating repetitive simulation and analysis tasks. BMC Syst Biol 11, 46

Hoops S, Sahle S, Gauges R, Lee C, Pahle J, Simus N, Singhal M, Xu L, Mendes P, Kummer U, 2006. COPASI--a COmplex PAthway Simulator. Bioinformatics 22(24), 3067-3074

T. Runarsson and X. Yao, 2000. Stochastic ranking for constrained evolutionary optimization. EEE Trans Evolutionary Comput. 4, 284-294
