# Supplementary material for: PTEN Regulates PI(3,4)P2 Signaling Downstream of Class I PI3K
Source: Mol Cell. 2017 Nov 2;68(3):566–580.e10. doi: 10.1016/j.molcel.2017.09.024 (PMC5678281; doi:10.1016/j.molcel.2017.09.024)
Supplement: Document S2. Article plus Supplemental Information [file mmc5.pdf]

Article

Molecular Cell

PTEN Regulates $PI(3,4)P_2$ Signaling Downstream of Class I PI3K

Graphical Abstract

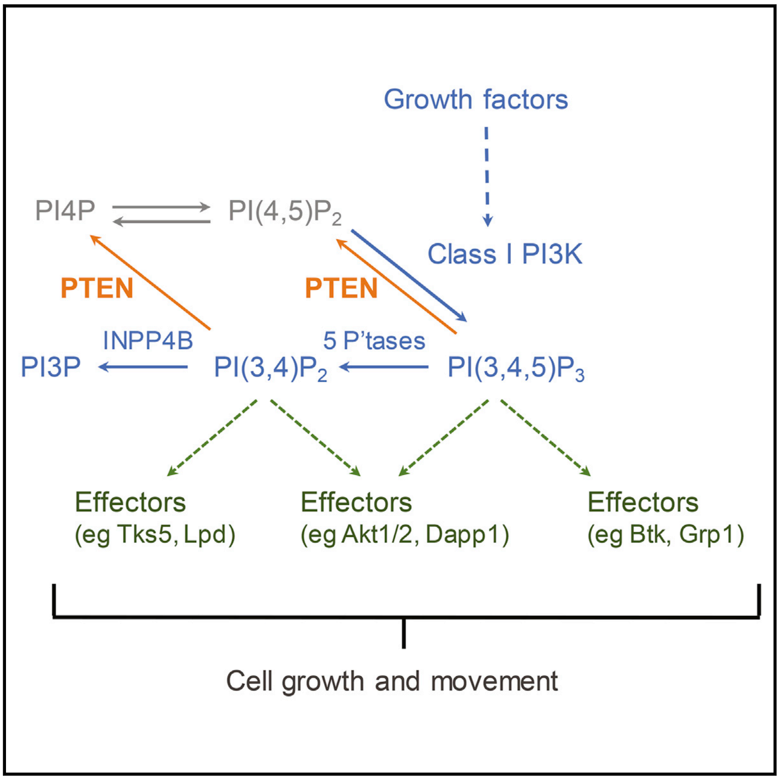

Highlights

- PTEN is a $PI(3,4)P_2$ 3-phosphatase
- PTEN and INPP4B regulate $PI(3,4)P_2$ accumulation downstream of class I PI3K
- PTEN regulates $PI(3,4)P_2$-dependent activation of Akt and formation of invadopodia
- $PI(3,4)P_2$ signaling may play a role in the tumor suppressor function of PTEN

Authors

Mouhannad Malek, Anna Kielkowska, Tamara Chessa, ..., Jonathan Clark, Phillip T. Hawkins, Len R. Stephens

Correspondence

phillip.hawkins@babraham.ac.uk (P.T.H.),
len.stephens@babraham.ac.uk (L.R.S.)

In Brief

Malek et al. show that the tumor suppressor PTEN acts as a $PI(3,4)P_2$ 3-phosphatase within the growth factor-stimulated PI3K signaling network, in addition to its accepted role as a $PI(3,4,5)P_3$ 3-phosphatase. This suggests that specific $PI(3,4)P_2$ effector functions, such as invadopodia formation, play a role in the PTEN-loss-of-function phenotype.

Malek et al., 2017, Molecular Cell *68*, 566–580
November 2, 2017 © 2017 The Authors. Published by Elsevier Inc.
https://doi.org/10.1016/j.molcel.2017.09.024

CellPress

PTEN Regulates $PI(3,4)P_2$ Signaling Downstream of Class I PI3K

Mouhannad Malek,[1,8] Anna Kielkowska,[1,8] Tamara Chessa,[1] Karen E. Anderson,[1] David Barneda,[1,5] Pınar Pir,[1] Hiroki Nakanishi,[2] Satoshi Eguchi,[2] Atsushi Koizumi,[3] Junko Sasaki,[2] Véronique Juvin,[1] Vladimir Y. Kiselev,[1] Izabella Niewczas,[1] Alexander Gray,[4] Alexandre Valayer,[1] Dominik Spensberger,[1] Marine Imbert,[1] Sergio Felisbino,[6] Tomonori Habuchi,[3] Soren Beinke,[7] Sabina Cosulich,[5] Nicolas Le Novère,[1] Takehiko Sasaki,[2] Jonathan Clark,[1] Phillip T. Hawkins,[1,9,10,*] and Len R. Stephens[1,9,*]
[1]Signalling Programme, Babraham Institute, Cambridge, UK
[2]Department of Medical Biology
[3]Department of Urology
Akita University Graduate School of Medicine, 1-1-1 Hondo, Akita, Japan
[4]School of Life Sciences, University of Dundee, Dow St., Dundee, UK
[5]AstraZeneca R&D Cambridge, CRUK Cambridge Institute, Cambridge, UK
[6]Department of Morphology, Institute of Biosciences of Botucatu, Sao Paulo State University – UNESP, Botucatu, Sao Paulo, Brazil
[7]Refractory Respiratory Inflammation Discovery Performance Unit, GlaxoSmithKline, Stevenage, UK
[8]These authors contributed equally
[9]Senior author
[10]Lead Contact
*Correspondence: phillip.hawkins@babraham.ac.uk (P.T.H.), len.stephens@babraham.ac.uk (L.R.S.)
https://doi.org/10.1016/j.molcel.2017.09.024

SUMMARY

The PI3K signaling pathway regulates cell growth and movement and is heavily mutated in cancer. Class I PI3Ks synthesize the lipid messenger $PI(3,4,5)P_3$. $PI(3,4,5)P_3$ can be dephosphorylated by 3- or 5-phosphatases, the latter producing $PI(3,4)P_2$. The PTEN tumor suppressor is thought to function primarily as a $PI(3,4,5)P_3$ 3-phosphatase, limiting activation of this pathway. Here we show that PTEN also functions as a $PI(3,4)P_2$ 3-phosphatase, both in vitro and in vivo. PTEN is a major $PI(3,4)P_2$ phosphatase in Mcf10a cytosol, and loss of PTEN and INPP4B, a known $PI(3,4)P_2$ 4-phosphatase, leads to synergistic accumulation of $PI(3,4)P_2$, which correlated with increased invadopodia in epidermal growth factor (EGF)-stimulated cells. PTEN deletion increased $PI(3,4)P_2$ levels in a mouse model of prostate cancer, and it inversely correlated with $PI(3,4)P_2$ levels across several EGF-stimulated prostate and breast cancer lines. These results point to a role for $PI(3,4)P_2$ in the phenotype caused by loss-of-function mutations or deletions in PTEN.

INTRODUCTION

The class I PI3K-signaling pathway is part of the large regulatory network that allows various cell surface receptors to control cell function (Hawkins and Stephens, 2015; Vanhaesebroeck et al., 2010). This pathway plays a particularly important role in the mechanisms that allow growth factor receptors to regulate cell growth. Growth factors stimulate class I PI3Ks to catalyze the phosphorylation of the membrane lipid $PI(4,5)P_2$ to form $PI(3,4,5)P_3$. $PI(3,4,5)P_3$ is retained in the lipid bilayer and promotes the translocation and/or activation of a variety of effectors that recognize the head group of this lipid with appropriate affinity and specificity. The best studied of these effectors are the PH domain-containing serine/threonine protein kinases PDK-1 and AKT1/2, which indirectly regulate the mTORC1 complex and promote anabolic growth and survival (Dibble and Cantley, 2015; Engelman et al., 2006). This pathway is heavily mutated in human cancers, harboring several prevalent oncogenes, including genes encoding PI3K subunits and AKT, and also several tumor suppressors, for example, PTEN and INPP4B (Fruman and Rommel, 2014; Mayer and Arteaga, 2016; Okkenhaug et al., 2016; Thorpe et al., 2015).

$PI(3,4,5)P_3$ can be dephosphorylated by 3- and 5-phosphatases to form $PI(4,5)P_2$ and $PI(3,4)P_2$, respectively. The best-studied 3-phosphatase is PTEN, and loss-of-function mutants cause significant elevations in $PI(3,4,5)P_3$ in various cell and animal models (Hollander et al., 2011). The major 5-phosphatases that act on $PI(3,4,5)P_3$ are less clear; good evidence has been provided that SHIP1 and -2 can regulate $PI(3,4,5)P_3$ levels in leukocytes and other tissues, but recent studies suggest other 5-phosphatases may also play a role, depending on cell context (Dyson et al., 2012; Erneux et al., 2011; Ooms et al., 2015).

The relative flux through 3- versus 5-dephosphorylation of $PI(3,4,5)P_3$ is also unclear, as is the physiological significance of dephosphorylation via either route. Removal of the 5-phosphate from $PI(3,4,5)P_3$ produces $PI(3,4)P_2$, and recent evidence points to additional signaling roles for this lipid. $PI(3,4)P_2$ can bind to some effectors with similar affinity to $PI(3,4,5)P_3$, for example, AKT or DAPP1, but specific roles for this lipid have also been defined, particularly in the regulation of actin

 © 2017 The Authors. Published by Elsevier Inc.
This is an open access article under the CC BY license (http://creativecommons.org/licenses/by/4.0/).

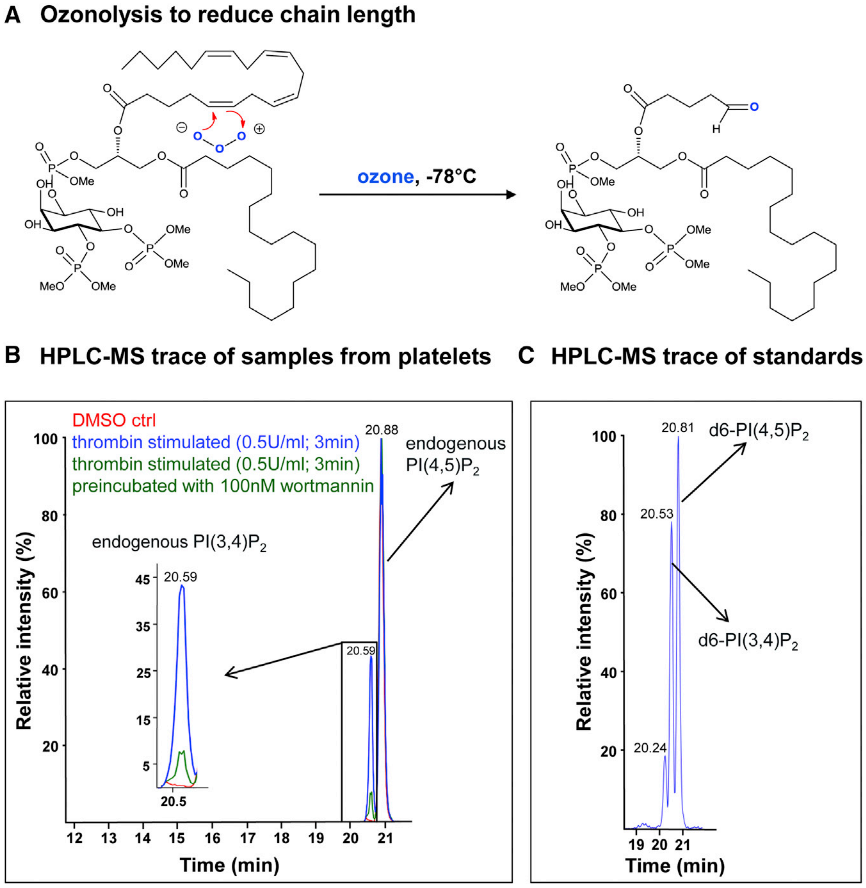

Figure 1. An HPLC-MS Method for Measuring $PI(3,4)P_2$ and $PI(4,5)P_2$

(A) An illustration of the reaction of ozone with methylated stearoyl/arachidonoyl $PI(4,5)P_2$.

(B) HPLC-MS traces derived from human platelets, showing the separation of molecules derived from endogenous stearoyl/arachidonoyl $PI(3,4)P_2$ and stearoyl/arachidonoyl $PI(4,5)P_2$. Note: $PI(3,5)P_2$ migrates with a slightly greater retention time than $PI(4,5)P_2$ in this system, but it is present at such low endogenous levels relative to $PI(4,5)P_2$ that it is below the levels of detection.

(C) A typical HPLC-MS trace derived from the separation of molecules derived from chemically synthesized d6-stearoyl/arachidonoyl $PI(3,4)P_2$ and d6-stearoyl/arachidonoyl $PI(4,5)P_2$ added as internal standards to a human platelet cell extract. Methods: Lipid extracts were prepared, treated with TMS-diazomethane and ozone, and the resulting molecules were separated by HPLC on a C18 column, as described in the STAR Methods. Supporting information is presented in Figure S1.

mesh-works associated with endocytic structures, lamellipodia, and podosomes/invadopodia (Hawkins and Stephens, 2016; Li and Marshall, 2015). Further, INPP4B has been recently identified as a conditional tumor suppressor, and a plausible mechanism of action has been constructed based on its ability to act as a specific $PI(3,4)P_2$ 4-phosphatase, thus limiting the activation of AKT (Fedele et al., 2010; Gewinner et al., 2009).

A major problem with defining the impact of specific phosphatases in shaping the $PI(3,4,5)P_3$ and $PI(3,4)P_2$ signals generated by activation of class I PI3Ks is current technical limitations in their quantitative measurement. Historically, the most accurate method for measuring these lipids has been radiolabelling, lipid extraction, deacylation, and separation of the relevant head groups by anion-exchange high-performance liquid chromatography (HPLC), which is laborious, requires substantial amounts of radiolabelled precursors, and cannot be applied to tissue biopsies. Recently, we described an approach to measure $PI(3,4,5)P_3$ by HPLC-mass spectrometry (MS) that circumvents many of these issues, but this method does not distinguish between regio-isomers of the same mass, for example, $PI(3,4)P_2$ and $PI(4,5)P_2$ (Clark et al., 2011).

We describe here an extension to our MS method, which allows HPLC separation and measurement of $PI(3,4)P_2$ and $PI(4,5)P_2$. We then applied this and existing methods to execute a systematic screen for the impact of gene deletion and/or small interfering RNA (siRNA) suppression of known phosphoinositide phosphatases on shaping $PI(3,4,5)P_3$ and $PI(3,4)P_2$ signals in response to growth factor stimulation. These and follow-up studies led us to discover that PTEN acts as a major $PI(3,4)P_2$ 3-phosphatase, in addition to its known role as a $PI(3,4,5)P_3$ 3-phosphatase. Moreover, deletion of PTEN in a mouse model of prostate cancer results in very high levels of $PI(3,4)P_2$ accumulation in hyperplasic epithelial cells, revealing another mechanism by which PTEN acts as a tumor suppressor.

RESULTS

A Method to Measure $PI(3,4)P_2$ and $PI(4,5)P_2$ by HPLC-MS

We have previously shown that methylation of the acidic phosphate groups of polyphosphoinositides with trimethylsilyl (TMS)-diazomethane allows sensitive detection of these molecules by HPLC-electrospray ionization-mass spectrometry (HPLC-ESI-MS) (Kielkowska et al., 2014). This method uses reverse-phase chromatography on a C4 column, which does not allow separation of $PI(3,4)P_2$ and $PI(4,5)P_2$. We have now developed a modification to this method based on ozone-catalyzed cleavage of C = C double bonds to reduce acyl chain length (Figure 1A). Chromatography of these shortened derivatives on a C18-amide column yields a sufficiently good separation of molecules derived from the most common species of $PI(3,4)P_2$ and $PI(4,5)P_2$ found in mammalian tissues (C38:4; stearoyl/arachidonoyl) to allow an independent estimate of their relative amounts (Figures 1B and 1C). This method was able to detect PI3K-dependent accumulation of $PI(3,4)P_2$ in thrombin-stimulated human platelets (Figure 1B), a response previously defined using traditional radiolabeling approaches (Giuriato et al., 2003; Rittenhouse, 1996). Further, the routine inclusion

of synthetic d6-labeled standards allowed us to accurately quantify the amounts of endogenous C38:4 PI(3,4)P_2 and PI(4,5)P_2 in our biological extracts (Figures S1A and S1B).

Identification of the Major Phosphatases Controlling PI(3,4,5)P_3 and PI(3,4)P_2 Accumulation in EGF-Stimulated Mcf10a Cells

We disrupted expression of phosphoinositide phosphatases in Mcf10a cells, and then we used our new and existing HPLC-MS methods to measure the effect on epidermal growth factor (EGF)-stimulated accumulation of PI(3,4,5)P_3 and PI(3,4)P_2. Initially, we employed an siRNA screen directed against all phosphoinositide phosphatases previously reported to act on these two lipids and expressed at significant levels in these cells (INPP5B, INPP5E, INPP5F, INPP5J, INPP5K, SHIP1, SHIP2, SYNJ1/2, OCRL, and PTEN; Balla, 2013; Kiselev et al., 2015), and we focused on measuring PI(3,4,5)P_3 in both starved and then EGF-stimulated wild-type (WT) cells (Figure S2A). We also evaluated the impact of suppressing selected phosphatases (SHIP1, SHIP2, INPP4A, and INPP4B) in a *PTEN*$^{-/-}$ isogenic cell line (PTEN-knockout [KO]) (Figure S2A). We then followed up these initial studies by disrupting expression of INPP4B and SHIP2 in both WT and PTEN-KO cells by CRISPR/Cas9-mediated gene editing and measuring PI, PIP, PI(3,4)P_2, PI(4,5)P_2, and PI(3,4,5)P_3 in selected starved and EGF-stimulated cells.

In WT cells, EGF stimulated a rapid and transient increase in the levels of PI(3,4,5)P_3 (Figure 2A), a small transient increase in PIP (Figure S2B), and barely detectable changes in PI(3,4)P_2 (Figure 2B) and PI(4,5)P_2 (Figure 2C). These responses are consistent with much previous work describing EGF stimulation of PI3Ks and PIPKs under these conditions (Anderson et al., 2016; Jackson et al., 1992). The only genetic manipulations that reliably altered the PI(3,4,5)P_3 response to EGF were knockdown (KD) or deletion of PTEN or SHIP2 (Figures 2A and S2A). In each case, the effect was relatively modest, causing an ~25%–50% increase in peak PI(3,4,5)P_3 accumulation at 1 min. However, siRNA knockdown of SHIP2 in PTEN-KO cells or CRISPR/Cas9-mediated deletion of SHIP2 expression in PTEN-KO cells resulted in a substantial, synergistic elevation in peak PI(3,4,5)P_3 levels (3- to 4-fold), though in each case the PI(3,4,5)P_3 response remained transient, with levels falling within 5–15 min of EGF stimulation (Figure 2A). These results clearly identify PTEN and SHIP2 as phosphatases that act on PI(3,4,5)P_3 during EGF stimulation, but they suggest that each can substantially compensate for loss of the other.

We also note that loss of PTEN caused an apparent drop in the levels of PI(4,5)P_2 (Figure 2C) and a small increase in PIP (Figure S2B). However, these changes were confined to the C38:4 species of these lipids and were not reflected in the total PIP_2 and PIP pools (data not shown), and, therefore, they probably result from an indirect effect on acyl composition.

Combined knockdown of INPP4A and B did not produce a measurable increase in the levels of PI(3,4)P_2 under either starved or EGF-stimulated conditions (Figure 2B). This was surprising, given that INPP4A and B are thought to be the major phosphatases controlling the levels of PI(3,4)P_2 in mammalian cells (Balla, 2013). However, knockdown of INPP4A/B in PTEN-KO cells produced a very large accumulation of PI(3,4)P_2 in response to EGF (Figure 2B). The peak PI(3,4)P_2 accumulation was at 5 min, reaching levels ~10-fold greater than the peak levels of PI(3,4,5)P_3 in the same cells and amounting to ~20% of the total PIP_2 pool (Figures 2A–2C).

We used CRISPR/Cas9 editing in PTEN-KO cells to eliminate expression of INPP4B. EGF stimulated very similar accumulations of PI(3,4)P_2 in these cells compared to our analogous siRNA knockdown studies (Figure 2B). Further, siRNA knockdown of INPP4A in these cells did not increase levels of PI(3,4)P_2 (Figure 2B), indicating INPP4B and not INPP4A was acting together with PTEN to control the levels of this lipid.

EGF-stimulated EGF receptor (EGFR) auto-phosphorylation was very similar across the relevant knockdown and knockout cell lines (Figure S2C). Further, we saw no evidence of compensatory increases in phosphatase expression in lines in which expression of PTEN, INPP4B, or SHIP2 had been deleted (Figures S3A and S3B). Interestingly, however, expression of INPP4B was significantly reduced in the PTEN-KO line (Figure S3B), possibly contributing to the small elevation in PI(3,4)P_2 consistently seen in these cells across multiple experiments.

PI(3,4,5)P_3 and PI(3,4)P_2 Accumulate in the Plasma Membrane of EGF-Stimulated Mcf10a Cells

We used confocal fluorescence imaging with EGFP-PH-GRP1 and mCherry-PH-TAPP1 reporters to visualize the PI(3,4,5)P_3 and PI(3,4)P_2 pools in WT, PTEN-KO, SHIP2-KD, SHIP2-KD,PTEN-KO, and INPP4A/B-KD,PTEN-KO cells. EGF stimulated clear accumulation of the PI(3,4,5)P_3 reporter in the proximity of the plasma membrane in all cells examined (Figure 3A), and EGF stimulated clear accumulation of the PI(3,4)P_2 reporter in the proximity of the plasma membrane in INPP4A/B-KD,PTEN-KO cells (Figure 3B). Further, this pattern of accumulation of the PI(3,4)P_2 reporter in these cells was confirmed by immunofluorescence using an anti-PI(3,4)P_2 antibody (Figure 3C).

The Accumulation of PI(3,4)P_2 in EGF-Stimulated Mcf10a Cells Is Class I PI3K Dependent

Several alternative pathways for cell surface receptor-stimulated accumulation of PI(3,4)P_2 have been suggested, including 5-dephosphorylation of PI(3,4,5)P_3, 3-phosphorylation of PI4P by class I PI3Ks, 3-phosphorylation of PI4P by class II PI3Ks, or sequential phosphorylation from PI3P by unidentified kinases (Posor et al., 2015; Rittenhouse, 1996; Stephens et al., 1993). We used a combination of experimentation and mathematical modeling to identify the major pathways that generate PI(3,4,5)P_3 and PI(3,4)P_2 in our system.

We first evaluated the effect of a combination of small molecule inhibitors with widely different potencies for the inhibition of class I PI3Kα/β versus class II PI3Kα/β (PIK75 and TGX115; see Figure 4A for relevant IC_{50}s). These molecules inhibited the EGF-stimulated accumulation of PI(3,4,5)P_3 and PI(3,4)P_2 in INPP4A/B-KD,PTEN-KO cells with very similar potency (Figure 4A), suggesting both were derived from the same source and that this source was a class I PI3K. This role for a class I PI3K was supported by the sensitivity of both PI(3,4,5)P_3 and PI(3,4)P_2 accumulation to PI-103 and the PI3Kα-selective inhibitor BYL-719 (Figures S4A and S4B). The sensitivity to BYL-719

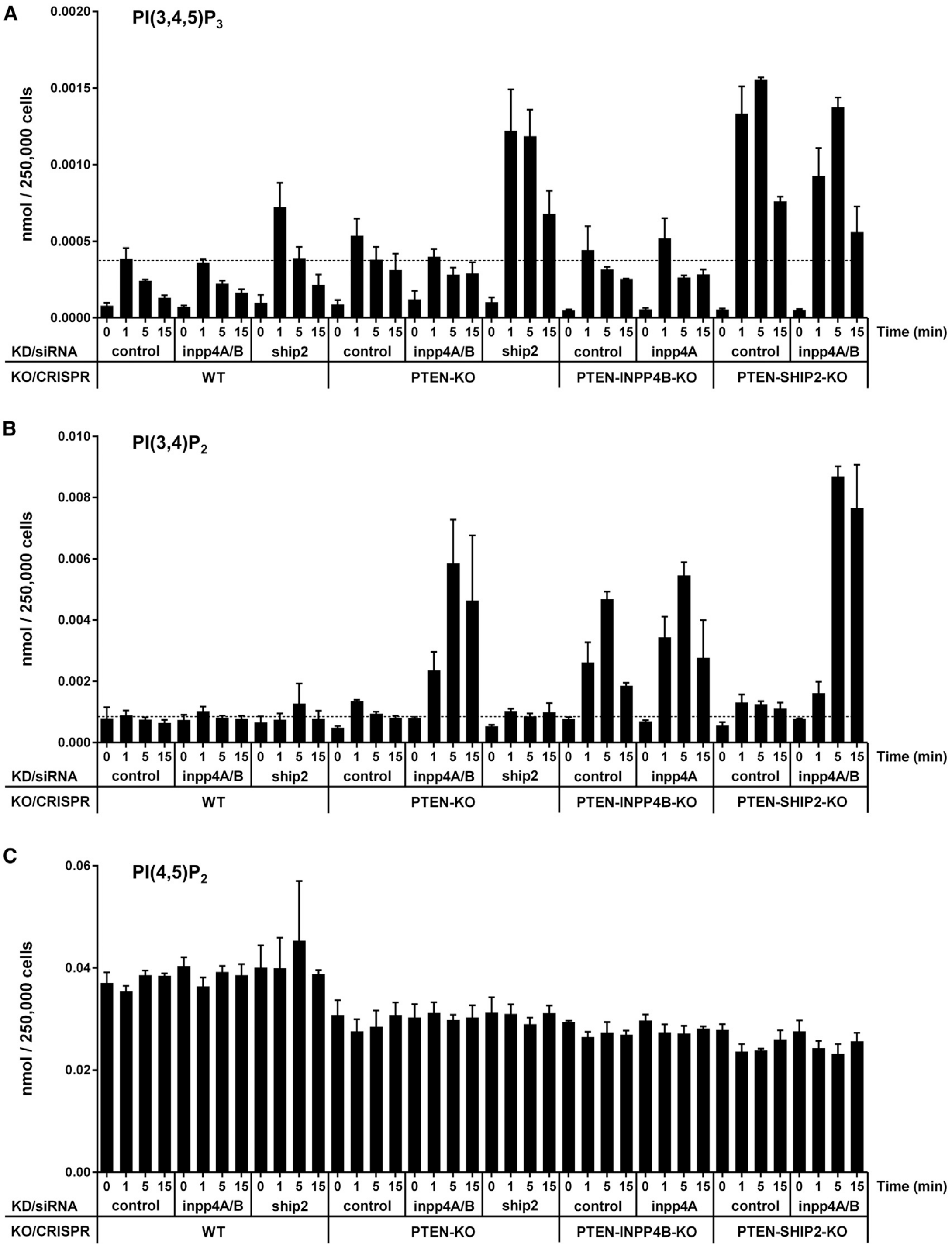

Figure 2. The Identification of Phosphatases that Shape $PI(3,4,5)P_3$ and $PI(3,4)P_2$ Signals in EGF-Stimulated Mcf10a Cells

(A) $PI(3,4,5)P_3$ levels in Mcf10a cells treated with EGF (10 ng/mL) for 0, 1, 5, or 15 min.

(B) $PI(3,4)P_2$ levels in Mcf10a cells treated with EGF (10 ng/mL) for 0, 1, 5, or 15 min.

(C) $PI(4,5)P_2$ levels in Mcf10a cells treated with EGF (10 ng/mL) for 0, 1, 5, or 15 min.

Methods: Isogenic WT or *PTEN*$^{-/-}$ Mcf10a cells were genetically manipulated through siRNA-mediated suppression or CRISPR-gene editing, as indicated, then starved and stimulated with EGF. Measurement of $PI(3,4,5)P_3$ or $PI(3,4)P_2$ and $PI(4,5)P_2$ was performed by HPLC-MS using C4 or C18 columns, respectively, and data represent means ± SD of 3 biological replicates (for siRNA suppression in WT or PTEN-KO cells) or 3 technical replicates (for PTEN-INPP4B-KO or PTEN-SHIP2-KO cells).

Supporting information is presented in Figures S2 and S3.

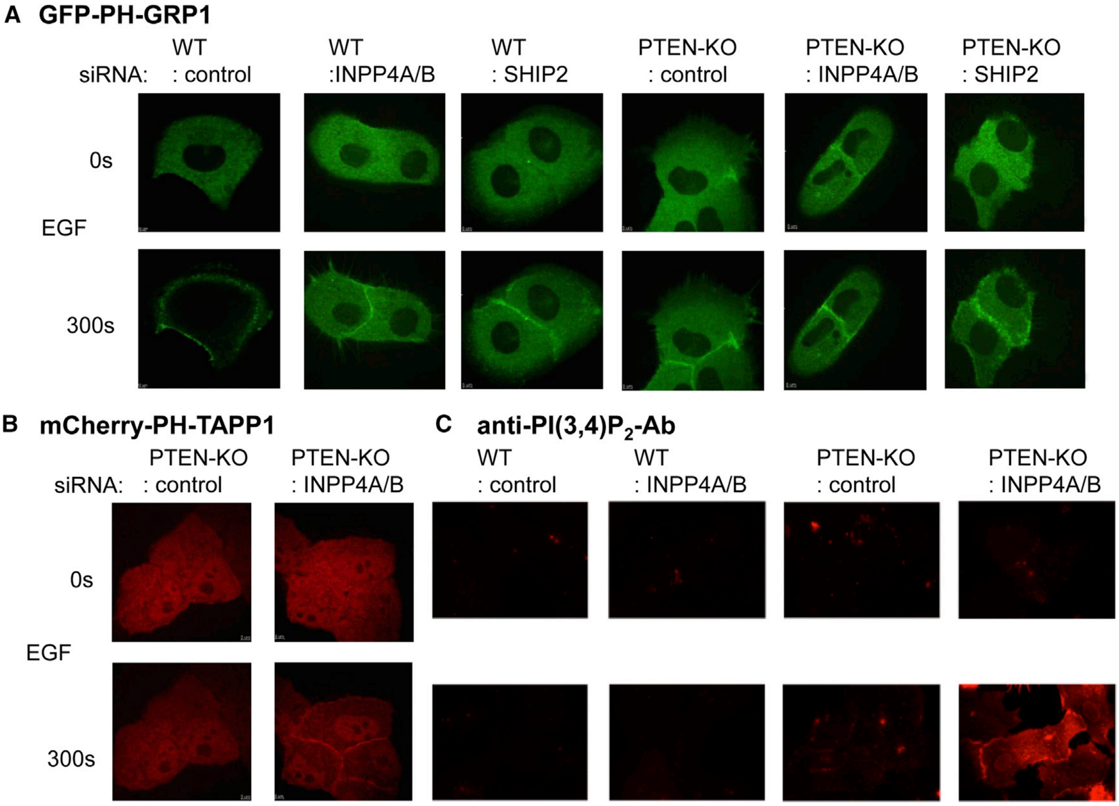

Figure 3. The Subcellular Location of PI(3,4,5)P_3 and PI(3,4)P_2 in EGF-Stimulated Mcf10a Cells

(A) Confocal fluorescent images of WT or *PTEN*$^{-/-}$ Mcf10a cells stably expressing the EGFP-GRP1-PH domain. Cells were treated with the indicated siRNA, starved, and then stimulated with EGF (10 ng/mL) for 0 or 300 s.

(B) Confocal fluorescent images of WT or *PTEN*$^{-/-}$ Mcf10a cells stably expressing the mCherry-TAPP1-PH domain. Cells were treated with the indicated siRNA, starved, and then stimulated with EGF (10 ng/mL) for 0 or 300 s.

(C) Wide-field fluorescent images of WT or *PTEN*$^{-/-}$ Mcf10a cells treated with the indicated siRNA, starved, and then stimulated with EGF (10 ng/mL) for 0 or 300 s. PI(3,4)P_2 was visualized with an anti-PI(3,4)P_2 antibody.

is in agreement with previous studies indicating EGF signals primarily through PI3Kα in these cells (Juvin et al., 2013). Further, the lack of involvement of class II PI3Ks in generating PI(3,4)P_2 responses was confirmed by siRNA knockdown of these enzymes (Figures S4C and S4D).

The identification of a class I PI3K as the source of PI(3,4)P_2 accumulation in these experiments implied a major role for 5-phosphatase-mediated dephosphorylation of PI(3,4,5)P_3. Surprisingly, given that we had demonstrated an involvement of SHIP2 in regulating PI(3,4,5)P_3 levels, siRNA knockdown of INPP4A/B caused similar accumulations of PI(3,4)P_2 in PTEN-KO cells and PTEN-KO cells in which SHIP2 expression had also been deleted by CRISPR/Cas9, though possibly with slightly slower kinetics (Figure 2B). We reasoned this may be due to redundancy among multiple PI(3,4,5)P_3 5-phosphatases, which could maintain flux through 5-dephosphorylation in the presence of elevated levels of PI(3,4,5)P_3 (caused by the loss of both SHIP2 and PTEN). We therefore investigated the effect of multiple knockdowns of known 5-phosphatases in INPP4A/B-KD,PTEN-SHIP2-KO cells. Of the combinations examined, only combined knockdown of SHIP1, SYNJ1/2, INPP5F, and INPP5B caused significant elevations in PI(3,4,5)P_3 and a modest reduction in PI(3,4)P_2 (Figure S4E).

It therefore remained possible that most of the PI(3,4)P_2 produced on EGF stimulation may be derived directly by class I PI3K-mediated 3-phosphorylation of PI4P, rather than indirectly via 5-dephosphorylation of PI(3,4,5)P_3. To estimate the flux through PI(3,4,5)P_3 dephosphorylation, we added 1 μM PI-103 1 min after adding EGF, to prevent further activity of class I PI3K, and then we monitored the fall in PI(3,4,5)P_3 levels (Figure 4B). PI(3,4,5)P_3 levels dropped so quickly in WT cells that it was difficult to model the kinetics and derive an accurate rate of dephosphorylation, but the half-life was <5 s (Figure 4B, upper left panel). Loss of PTEN, knockdown of SHIP2, and combined loss of PTEN and knockdown of SHIP2 slowed the fractional rate of PI(3,4,5)P_3 removal, but the higher starting levels of PI(3,4,5)P_3 maintained a high rate of PI(3,4,5)P_3 dephosphorylation (Figure 4B, upper right panel).

We built mathematical models to test our hypotheses on PI(3,4,5)P_3 and PI(3,4)P_2 dephosphorylations (Figures 4C–4E; STAR Methods; Data S1 and S2). The minimal model able to explain all of our observations includes the activation of EGFRs

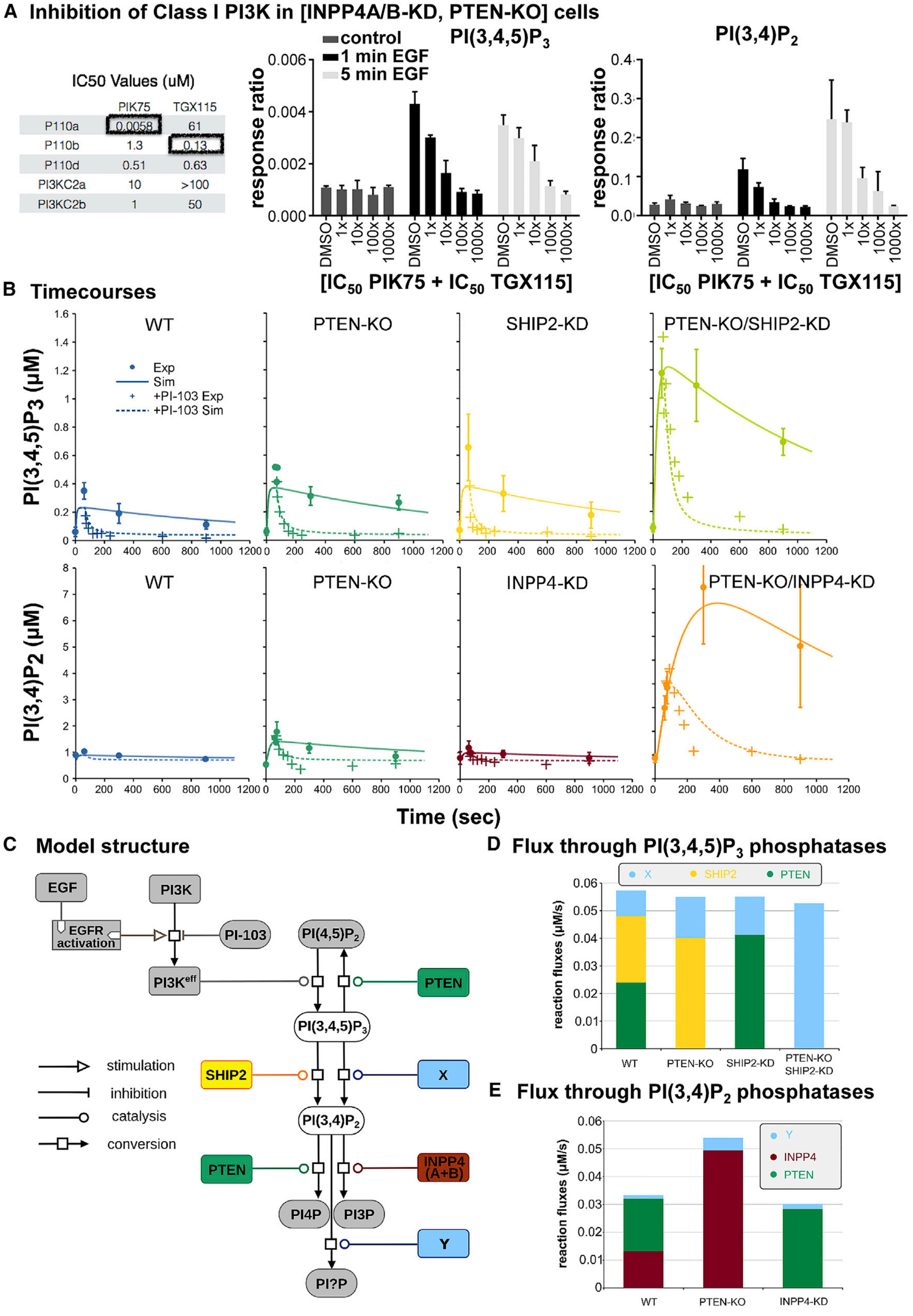

(legend on next page)

and the subsequent stimulation of class I PI3K activity, as well as the formation and consumption of PI(3,4,5)P_3, PI(3,4)P_2, PI(4,5)P_2, PI3P, and PI4P (Figure 4C). We parameterized the model using all the time course measurements in WT, knockdown and knockout backgrounds, with and without PI3K inhibition (presence or absence of PI-103), using the genetic algorithm in COPASI software (Dalle Pezze and Le Novère, 2017; Mendes et al., 2009). Parameterization of alternative models with different kinetic expressions demonstrated that the best fit to the experimental data was obtained if the assumption is made that the phosphatases involved in class I PI3K-activated phosphoinositide signaling pathways operate in their linear range, i.e., they are not saturated with their lipid substrate (STAR Methods; Data S1 and S2). Simulations created by the operation of this model are depicted by continuous lines in Figure 4B.

The predicted ratio of flux through 5-phosphatase and 3-phosphatase attack on PI(3,4,5)P_3 is 1.4:1, suggesting a substantial fraction of PI(3,4,5)P_3 is recycled back to PI(4,5)P_2 upon class I PI3K activation (Figure 4D). When PTEN is genetically deleted, this recycling is not possible and PI(3,4)P_2 is produced at a higher rate, as a result of more PI(3,4,5)P_3 being available to 5-phosphatases (Figure 4D). EGF-stimulated accumulation of PI(3,4)P_2 in all mutant cells could be simulated if SHIP2 plus other 5-phosphatases (X) support PI(3,4)P_2 production with a relative flux of ~2.65:1 (Figure 4D). The observed level cannot be simulated if SHIP2 is the only 5-phosphatase processing PI(3,4,5)P_3.

This model predicts that both PTEN and INPP4B directly regulate PI(3,4)P_2 dephosphorylation, together with a further unknown phosphatase (Y). Simulations show that PTEN must act directly on PI(3,4)P_2 and not merely increase the levels of PI(3,4,5)P_3 and thence flux through its 5-dephosphorylation. In INPP4A/B-KD cells, all PI(3,4)P_2 dephosphorylation flux is routed through PTEN and the unknown phosphatase Y, with a relative flux of approximately 16:1 (Figure 4E). The model predicts that Y must provide about 6% of the total PI(3,4)P_2-phosphatase activity, based on the continued dephosphorylation of PI(3,4)P_2 in INPP4A/B-KD,PTEN-SHIP2-KO cells in the presence of PI-103 (Figure 4B, lower right panel). Candidates for the unknown phosphatase Y are the PTEN homologs TPTE or TPIP (Walker et al., 2001), however, there is no evidence to date that these proteins are expressed in Mcf10a cells (Wang et al., 2011).

The model is not able to simulate the basal levels of PI(3,4)P_2 in starved, unstimulated cells without invoking a separate pool of PI(3,4)P_2 that is both insensitive to EGF and is not synthesized by class I PI3K (PI(3,4)P_2_BG; STAR Methods; Data S1 and S2). We interrogated the nature of this EGF-insensitive pool by alternative MS analyses and [^{33}P]-Pi-radiolabelling (Figure S5). The results were inconclusive and it remains plausible that this pool is significantly contaminated with non-PI(3,4)P_2-derived molecules, leading to an overestimation of the class I PI3K-insensitive pool predicted by the modeling (see the legend to Figure S5 for more detailed arguments).

PTEN Directly Dephosphorylates PI(3,4)P_2 in Mcf10a Cytosol

A clear prediction of our mathematical model is that PTEN acts as a direct PI(3,4)P_2 phosphatase and provides around 57% of the total PI(3,4)P_2-phosphatase activity. PTEN has previously been shown to be a poor PI(3,4)P_2 phosphatase in assays with recombinant protein and simplified lipid substrates (McConnachie et al., 2003). We re-examined the potential for PTEN to act as a PI(3,4)P_2 phosphatase by constructing assays that were a better mimic of the physiological environment; this involved combining Mcf10a cytosol with a complex lipid substrate that contained phospholipids, cholesterol, sphingomyelin, and isotope-enriched d6-PI(3,4,5)P_3 or d6-PI(3,4)P_2 substrates (to avoid any ambiguity as to the origin of products derived from these substrates).

Under assay conditions resulting in less than 10% consumption of substrate, cytosol from WT cells dephosphorylated d6-PI(3,4,5)P_3 at both 3- and 5- positions, producing d6-PI(4,5)P_2 and d6-PI(3,4)P_2, respectively (Figures 5A and 5B). Cytosol from PTEN-KO cells produced no significant d6-PI(4,5)P_2, indicating PTEN is the only PI(3,4,5)P_3 3-phosphatase active under these conditions. Cytosol from cells lacking SHIP2

Figure 4. Evidence for Class I PI3K-Driven Accumulation of PI(3,4)P_2 in EGF-Stimulated Mcf10a Cells

(A) Measurements by HPLC-MS of PI(3,4,5)P_3 (left panel) or PI(3,4)P_2 (right panel) in INPP4BA/B-KD,PTEN-KO Mcf10a cells that were starved; pretreated for 20 min with the indicated dilutions of a mixture of PIK75 and TGX115 (1× represents 0.0058 μM PIK75 and 0.13 μM TGX115); and then stimulated with EGF (10 ng/mL) for 0, 1, or 5 min. Data are means ± SD of 3 technical replicates. The table shows IC_{50}s of PIK75 and TGX115 against relevant PI3K activities assayed in vitro.

(B) Time courses of PI(3,4,5)P_3 (top) and PI(3,4)P_2 (bottom) accumulation during EGF stimulation of WT, PTEN-KO, SHIP2-KD, INPP4A/B-KD, SHIP2-KD,PTEN-KO, and INPP4A/B-KD,PTEN-KO cells. Cells were starved and stimulated with EGF (10 ng/mL) at time 0. At 60 s post-EGF stimulation, samples were treated with either vehicle (DMSO; solid circles) or PI-103 (1 μM; crosses), and incubations continued for the times indicated. Data are represented as means ± SD of 3 biological replicates. Lines represent simulations of our mathematical model (see below) in the absence (continuous) or presence (dashed lines) of PI-103. Only INPP4A/B-KD,PTEN-KO cells exhibit a large increase in PI(3,4)P_2.

(C) List of biochemical reactions represented in the mathematical model, represented using Systems Biology Graphical Notation (SBGN) process descriptions (Le Novère et al., 2009).

(D) Simulated maximal fluxes of dephosphorylation of PI(3,4,5)P_3 into PI(4,5)P_2 by PTEN and into PI(3,4)P_2 by SHIP2 and X (the combination of all other relevant 5-phosphatases). In PTEN-KO cells, all PI(3,4,5)P_3 is converted into PI(3,4)P_2, SHIP2 and X compensating for the absence of PTEN. In SHIP2-KD cells, while X compensates partially for the lack of SHIP2, a larger share of the fluxes is re-routed toward PI(4,5)P_2. When both PTEN and SHIP2 are absent, the flux through X cannot fully compensate for the lack of both enzymes.

(E) Simulated maximal fluxes of dephosphorylation of PI(3,4)P_2 by INPP4B, PTEN, and an unknown phosphatase Y in WT, INPP4A/B-KD, and PTEN-KO cells. In the WT, the fluxes through INPP4B and PTEN are balanced. The fluxes are re-routed in the mutants. While an unknown phosphatase is required to accurately reproduce the experimental results, its activity remains very limited.

Supporting information is presented in Figures S4 and S5, the STAR Methods, and Data S1 and S2.

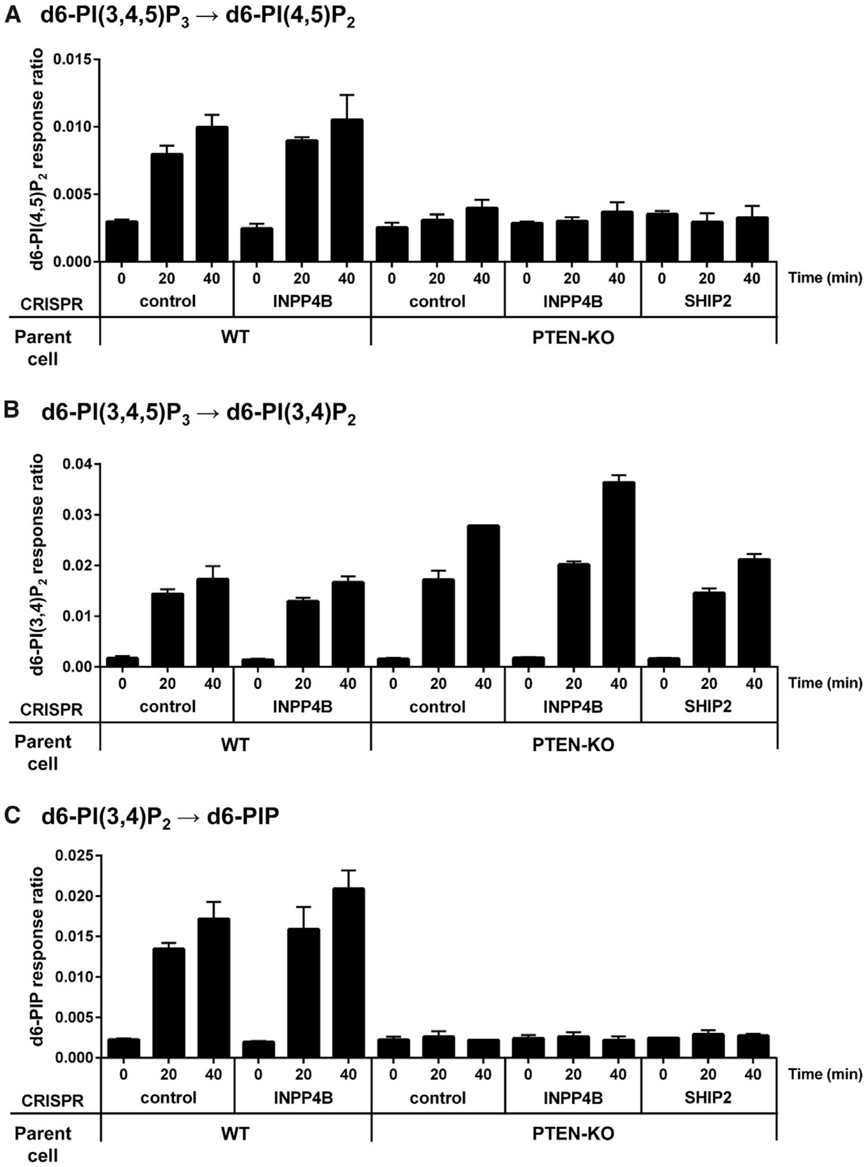

Figure 5. Dephosphorylation of $PI(3,4,5)P_3$ and $PI(3,4)P_2$ by Mcf10a Cytosol

(A) Measurement of 3-phosphatase activity against d6-$PI(3,4,5)P_3$ present in cytosol prepared from the indicated Mcf10a cell genotypes. Data are means ± SD of 3 technical replicates.

(B) Measurement of 5-phosphatase activity against d6-$PI(3,4,5)P_3$ present in cytosol prepared from the indicated Mcf10a cell genotypes. Data are means ± SD of 3 technical replicates.

(C) Measurement of d6-$PI(3,4)P_2$-phosphatase activity present in cytosol prepared from the indicated Mcf10a cell genotypes. Data are means ± SD of 3 technical replicates.

Methods: Mcf10a cytosol was prepared and incubated with liposomes containing the indicated d6-labeled phosphoinositide at 30°C, for the times indicated, and then lipids were extracted and measured by HPLC-MS, as described in the STAR Methods.

Supporting information is presented in Figure S6.

produced similar amounts of d6-$PI(3,4)P_2$ to cytosol derived from cells containing SHIP2, consistent with the conclusion above that Mcf10a cells contain multiple $PI(3,4,5)P_3$ 5-phosphatase activities.

Cytosol from cells lacking INPP4B was able to dephosphorylate d6-$PI(3,4)P_2$, generating d6-PIP, at a very similar rate to cytosol from WT cells (Figure 5C). Remarkably, however, cytosol from cells lacking PTEN produced no measurable d6-PIP in these assays (Figure 5C). The dephosphorylation of d6-$PI(3,4)P_2$ by PTEN-KO cytosol could be rescued by the addition of catalytically active, but not catalytically dead, recombinant PTEN (Figure S6B), demonstrating that PTEN can act as a direct $PI(3,4)P_2$ phosphatase under these conditions. Further, we were able to modify our HPLC-MS method to distinguish between PI3P and PI4P (see the STAR Methods), and this was sufficient to identify the d6-PIP resulting from PTEN-dependent dephosphorylation of d6-$PI(3,4)P_2$ as PI4P and, hence, define PTEN as a $PI(3,4)P_2$ 3-phosphatase (Figure S6A).

$PI(3,4)P_2$ Accumulation in EGF-Stimulated Mcf10a Cells Correlates with the Activation of AKT and Increased Numbers of Invadopodia

Previous work has shown deletion of PTEN or INPP4B results in hyperactivation of AKT (Gewinner et al., 2009). We compared the EGF-stimulated phosphorylation of T308-AKT and S473-AKT in WT and genetically modified Mcf10a cells (Figures 6A and 6B). The knockdown of INPP4A/B or deletion of PTEN augmented the activation of AKT, and this was further increased by combined knockdown and deletion of these two enzymes (Figures 6A and 6B). The phosphorylation of AKT did not correlate closely with levels of $PI(3,4)P_2$, but this might be predicted from our lack of understanding of the relative efficiency with which $PI(3,4,5)P_3$ and $PI(3,4)P_2$ activate AKT.

$PI(3,4)P_2$ also has a separate, specific role in mediating Tks5-dependent formation of actin-rich structures called invadopodia (Seals et al., 2005; Sharma et al., 2013; Yamaguchi et al., 2011). These structures are involved in matrix degradation and have been implicated in metastasis (Leong et al., 2014; Seals et al., 2005). We measured the formation of invadopodia in transforming growth factor β (TGF-β)-primed Mcf10a cells grown on fluorescent-gelatin by correlating holes in the gelatin with focal accumulations of cortactin. We found that EGF stimulated a significant increase in the numbers of invadopodia and that this response was significantly augmented in cells lacking both PTEN and INPP4B (Figures 6C and 6D).

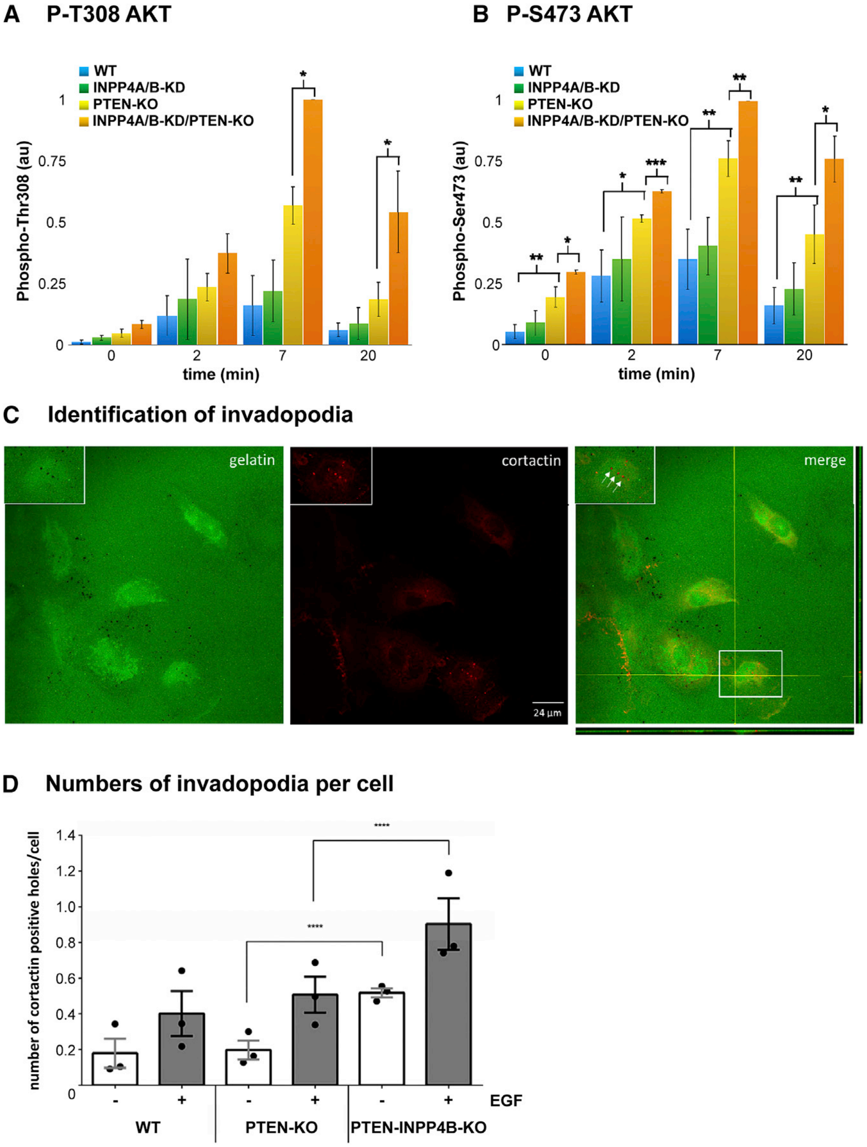

Figure 6. The Impact of Deleting PTEN and INPP4B on Activation of AKT and the Formation of Invadopodia in Mcf10a Cells

(A and B) Phosphorylation of Thr-308-AKT (A) and Ser-473-AKT (B) in WT or PTEN-KO Mcf10a cells treated with either Ctrl or INPP4A/B siRNA and then starved and stimulated for the indicated times with EGF (0.3 ng/mL). AKT phosphorylation was measured in cell lysates after SDS-PAGE, western blotting with the relevant antibody, and then normalization against an actin loading control (see the STAR Methods). Data are means ± SD of 3 technical replicates.

(C) An example of images taken of WT Mcf10a cells grown for 6 days in the presence of hTGF-β1 (10 ng/mL), plated on fluorescent gelatin for 2 hr, starved (4 hr), and then stimulated with EGF (20 ng/mL; 6 hr), before fixing and staining with an anti-cortactin antibody (see the STAR Methods). Invadopodia were identified by the co-localization of holes in the gelatin (green) with accumulations of cortactin (red; see arrows). Orthogonal projection of z stacked images shows cross-section through the gelatin surface (typically 1.5–2 μm) and invadopodia labeled with antibody against cortactin.The scale bar represents 24 μm.

(D) Quantification of invadopodia formed in Mcf10a cells of the indicated genotype. The data represent means ± SD of 3 biological replicates. Statistical analysis was done using Tukey's multiple comparisons test (with p values of $^{*}p < 0.05$, $^{**}p < 0.01$, $^{***}p < 0.001$, and $^{****}p < 0.0001$). The effect of EGF treatment was significant in all compared genotypes.

PTEN Regulates $PI(3,4)P_2$ Accumulation in a Mouse Model of Prostate Cancer

The above studies clearly identify PTEN as a major $PI(3,4)P_2$ phosphatase in vitro. To extend these observations to an in vivo context in which PTEN has been shown to play an important role, we investigated the prostate epithelial cell-specific deletion of PTEN in a mouse model of prostate cancer (Trotman et al., 2003). In the PB-Cre4 × PTEN model, expression of Cre-recombinase occurs from the onset of prostate development in newborn mice, and deletion of PTEN leads to hyperplasic growth, followed by prostate intraepithelial neoplasia (PIN) from 5 weeks and adenocarcinoma from 6 months (C. Sandi, personal communication).

We measured $PI(3,4)P_2$ and $PI(3,4,5)P_3$ accumulation by HPLC-MS in prostate biopsies taken from *Pten*$^{flox/flox}$,*PbCre*$^{-/-}$ (WT) or *Pten*$^{flox/flox}$,*PbCre*$^{+/-}$ (PTEN-KO) mice at 10 weeks of age (Figures 7A and 7B). We also visualized $PI(3,4)P_2$ accumulation by immunofluorescence in prostate sections taken from WT or PTEN-KO mice at 12 weeks of age (Figure 7C) and sections from WT; PTEN-KO; *Pten*$^{flox/flox}$, *PbCre*$^{-/-}$,*Inpp4b*$^{-/-}$ (INPP4B-KO; Kofuji et al., 2015); or *Pten*$^{flox/flox}$,*PbCre*$^{+/-}$, *Inpp4b*$^{-/-}$ (PTEN-INPP4B-KO) mice at 16 weeks of age (Figures 7D and 7E). Loss of PTEN alone caused a dramatic accumulation of $PI(3,4)P_2$, which amounted to ~50% of the levels of $PI(4,5)P_2$ in whole-prostate biopsies (Figures 7A and 7B; luminal epithelial cells represent ~70% of the total cells in these samples). Further, immunohistochemistry (IHC) analysis indicated preferential accumulation of $PI(3,4)P_2$ in the growing tips of hyperplasic acini in sections taken from PTEN-KO and PTEN-INPP4B-KO prostates at 16 weeks (Figure 7E). $PI(3,4)P_2$ accumulation also correlated closely with areas stained by an anti-phospho-S473-AKT antibody (Figure S7A). In PTEN-KO and PTEN-INPP4B-KO prostates at 16 weeks of age, $PI(3,4)P_2$ accumulation was also particularly evident in acini with surrounding sites of reactive stroma and where the smooth muscle cell layer had lost its integrity (Figure S7B). Hence, $PI(3,4)P_2$ accumulation correlated with both the earliest stages of tumor progression and later stages that constitute the first steps toward microinvasion.

CellPress

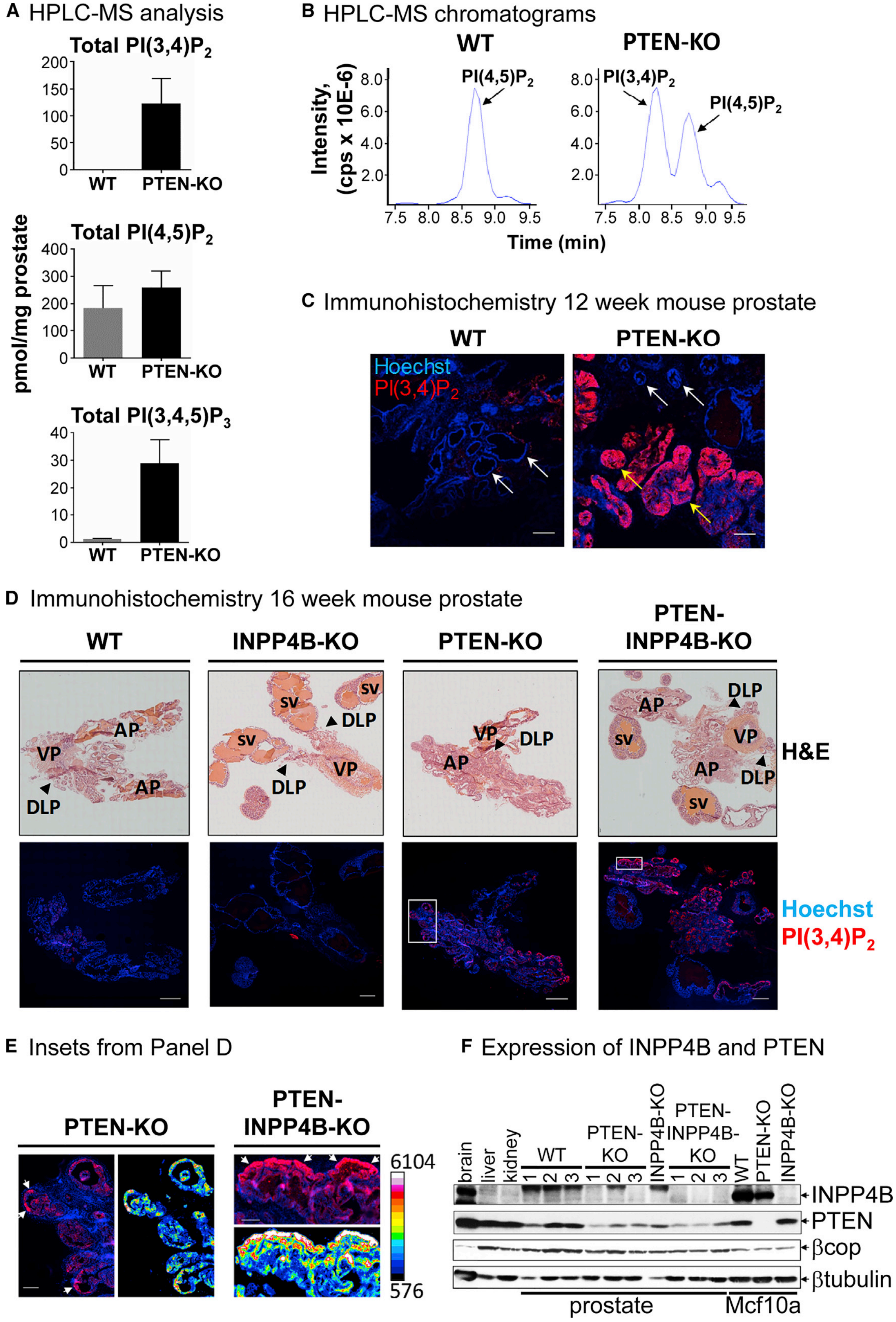

(legend on next page)

We found no evidence for PI(3,4)P_2 accumulation above WT in INPP4B-KO prostates and similar levels of PI(3,4)P_2 in PTEN-KO versus INPP4B-PTEN-KO prostates (Figures 7D and S7B). The remarkable accumulation of PI(3,4)P_2 in mouse prostate in response to the loss of PTEN alone and the reduced contribution from INPP4B in regulating PI(3,4)P_2 levels suggested there may be lower expression of INPP4B in mouse prostate compared to Mcf10a cells. We therefore compared the relative expression levels of PTEN and INPP4B in several mouse tissues and Mcf10a cells by western blotting. PTEN expression was similar across all samples analyzed, but expression of INPP4B was very variable, with high expression in mouse brain (Ferron and Vacher, 2006) and Mcf10a cells but undetectable expression in WT or PTEN-KO mouse prostate (Figure 7F). The low expression of INPP4B in mouse prostate was also confirmed by RNA sequencing (RNA-seq) (C. Sandi, personal communication), and it is consistent with similar rates of prostate cancer development in PTEN single-KO and PTEN-INPP4B double-KO mice (data not shown).

PTEN Regulates PI(3,4)P_2 Accumulation in Human Cancer Cell Lines

We measured PI(3,4)P_2 and PI(3,4,5)P_3 in a range of human prostate and breast cancer cells and also relative expression of INPP4B and PTEN (Figures S7C–S7F). Loss of PTEN generally correlated with increased basal and EGF-stimulated PI(3,4,5)P_3 responses and increased EGF-stimulated accumulation of PI(3,4)P_2. Two exceptions were T-47D and HCC-1187, which had normal expression of PTEN but large PI(3,4,5)P_3 responses. T-47D has previously been shown to possess an H1047 gain-of-function mutation and increased copy number of *PI3KCA* (Wu et al., 2005). HCC-1187 has been shown to have unusually high levels of phosphorylated EGFR and platelet-derived growth factor receptor (PDGFR) (Cuenca-López et al., 2014). The cell line with standout, selective accumulation of PI(3,4)P_2 in response to EGF was MDA-MB-436, which lacks expression of both PTEN and INPP4B, though this was not so obvious in the two other cell lines lacking both phosphatases (BT-549 and EVSA-T). Clearly, there will be many factors at play in determining the flux through 5-dephosphorylation of PI(3,4,5)P_3, including levels of active receptor, and thus a comparison between a relatively small number of cancer cells, with multiple differences in mutational status and gene expression, is difficult. Nevertheless, these results broadly support the idea that PTEN is likely to be a widespread regulator of PI(3,4)P_2 levels but that this role may be more apparent in cells with reduced expression of INPP4B.

Interestingly, prostate lines lacking PTEN exhibited relatively reduced responses to IGF-1 (Figure S7E). We suspect this is due to selective downregulation of the insulin/IGF-1/IRS-1-signaling pathway by PI3K/mTORC1-mediated feedback inhibition (T.C., unpublished data). However, the relative efficacy of different agonists to stimulate PI(3,4)P_2 accumulation is something that clearly requires further investigation.

DISCUSSION

We systematically screened for phosphoinositide phosphatases in Mcf10a cells that can selectively shape the PI(3,4,5)P_3 and PI(3,4)P_2 signals produced in response to EGF. While our results confirm the importance of PTEN and SHIP2 as phosphatases that regulate the accumulation of PI(3,4,5)P_3, they also point to a complex picture in these cells that is most easily explained by significant compensation among multiple phosphatases for the loss of any one individual enzyme. The extent to which these compensatory mechanisms reflect shared roles under normal conditions, are simply driven by the mass action effect of a rise in PI(3,4,5)P_3, or depend on additional activation mechanisms is unclear. Significant redundancy among 5-phosphatases may also explain the lack of prevalent tumor suppressors among this family, though the recent identification of PIPP (INPP5J) as a potential tumor suppressor in breast cancer points to contexts in which individual enzymes may predominate (Ooms et al., 2015). We also cannot rule out that significant PI(3,4)P_2 is produced by direct class I PI3K-catalyzed phosphorylation of PI4P, though previous studies showing analogous PI(3,4)P_2 production lags behind that of PI(3,4,5)P_3 (Hawkins et al., 1992) and our own observations that the rate of PI(3,4,5)P_3 degradation is sufficient to account for the rate of PI(3,4)P_2 accumulation suggest it is not necessary to invoke this explanation.

We clearly demonstrate that PTEN is an active PI(3,4)P_2 3-phosphatase in Mcf10a cytosol. This is surprising given that

Figure 7. The Impact of Deleting PTEN and INPP4B in Mouse Prostate

(A) Measurement by HPLC-MS of the indicated phosphoinositides in prostate biopsies taken from WT or PTEN-KO mice at 10 weeks of age. Data represent means ± SD of three individual mice for each group.

(B) Representative HPLC-MS chromatograms showing levels of PIP_2 in prostate biopsies taken from WT or PTEN-KO mice at 10 weeks of age.

(C) An example of Hoechst and anti-PI(3,4)P_2-stained sections of prostates taken from WT and PTEN-KO mice at 12 weeks of age. White arrows indicate normal acini and yellow arrows indicate regions in the prostate where acini exhibit HG-PIN. Images are confocal sections of a 12-μm specimen and scale bars represent 0.2 mm.

(D and E) H&E, Hoechst, and anti-PI(3,4)P_2-stained sections of prostates taken from WT, PTEN-KO, INPP4B-KO, or PTEN-INPP4B-KO mice at 16 weeks of age (D; scale bar represents 1 mm). Some areas of PTEN-KO and PTEN-INPP4B-KO sections are shown at higher magnification (E; scale bar represents 0.2 mm), and levels of anti-PI(3,4)P_2 staining are represented on the pseudo-color scale shown; examples of the tips of growing acini are indicated by white arrows. The images shown are typical of 3 prostate sections analyzed from 3 mice in each genotype. H&E images were stitched together using AxioVision 4 software and gaps automatically filled in using Adobe Photoshop; seminal vesicles (SV), anterior (A), dorsolateral (DLP), and ventral (V) lobes of the prostate are indicated by black arrows.

(F) A representative western blot is shown describing the relative expression of INPP4B and PTEN in the indicated mouse tissues (estimated 20 μg total protein loaded per lane) and human Mcf10a cell clones (15 μg total protein loaded per lane). This experiment was repeated 3 times with similar results. Note: in PTEN-KO prostates, Cre expression and hence PTEN deletion are restricted to prostate epithelial cells, which represent only ~70% of total cellular content.

Supporting information is presented in Figure S7.

careful in vitro studies have previously suggested that PI(3,4)P_2 is a very poor substrate for PTEN, at least compared to PI(3,4,5)P_3 (McConnachie et al., 2003). Further, PTEN was actually a much more effective PI(3,4)P_2 phosphatase in our assays than INPP4B, an established 4-phosphatase that is considered to catalyze the major route of PI(3,4)P_2 dephosphorylation, to form PI3P (Fedele et al., 2010; Gewinner et al., 2009). Phosphoinositide-metabolizing enzymes are notoriously susceptible to in vitro assay conditions, particularly with respect to substrate presentation (Irvine et al., 1984), and we think it is probable that the use of cytosol and a complex lipid interface in our assays (which included PI(4,5)P_2, an established co-factor for PTEN; Redfern et al., 2008) favored the detection of PTEN's PI(3,4)P_2-phosphatase activity compared to analogous previous studies. In intact Mcf10a cells, deletion of PTEN alone had a very small impact on EGF-stimulated accumulation of PI(3,4)P_2, and deletion of INPP4B alone had no discernible effect. However, combined deletion of PTEN and INPP4B had a very large, synergistic effect on EGF-stimulated PI(3,4)P_2 accumulation, suggesting that in the cellular environment these two enzymes can each compensate effectively for the other's absence with respect to PI(3,4)P_2 hydrolysis.

Our results indicate that the degree to which PI(3,4)P_2 produced by class I PI3K at the plasma membrane can be dephosphorylated by PTEN to form PI4P has been underestimated. This conclusion is supported by studies showing overexpressed PTEN can influence PI(3,4)P_2-reporter distributions in lymphocytes (Cheung et al., 2007), and it is also consistent with the relatively small accumulations of PI3P seen in most examples of class I PI3K activation (Hawkins et al., 1992; Stephens et al., 1991). It is also consistent with original observations that cell lysates can actively dephosphorylate PI(3,4)P_2 at the 3- position (Stephens et al., 1991). It is important to note, however, that PI(3,4)P_2 produced via different routes (e.g., class II PI3Ks) or in different locations (e.g., endosomes or clathrin-coated pits) is likely to be controlled by different phosphatases, and, at least in some of these contexts, the dephosphorylation of PI(3,4)P_2 by INPP4A/B to form PI3P is likely to predominate (Posor et al., 2015; Sasaki et al., 2010).

In mouse prostate, deleting PTEN alone had a profound effect on PI(3,4)P_2 levels; in 10-week-old prostates the levels of PI(3,4)P_2 rose from undetectable to approximately 50% of PI(4,5)P_2. Deletion of INPP4B alone had an insignificant effect on PI(3,4)P_2 accumulation, and combined deletion of INPP4B and PTEN had an insignificant impact above deletion of PTEN alone. Previous work has shown that deletion of PTEN in mouse prostate drives class I PI3K-dependent epithelial cell hyperplasia and tumor growth with 100% penetrance (Jia et al., 2008; Trotman et al., 2003), and, thus, our results represent a stunning example of how deletion of PTEN in vivo can result in massive PI(3,4)P_2 accumulation in the context of class I PI3K pathway activation.

The lesser impact of deleting INPP4B in mouse prostate versus Mcf10a cells reflects the relative abundance of this protein in these two tissues, and it suggests that different cells and tissues may vary with respect to the relative involvement of PTEN and INPP4B in regulating PI(3,4)P_2, a conclusion supported by our analysis of a limited collection of breast and prostate cancer cell lines. Direct evidence has been presented for a context-dependent role for INPP4B as a tumor suppressor, with deletion of *Inpp4b* driving tumor formation in mouse thyroid only in the absence of one allele of *Pten* (Vo and Fruman, 2015). A simple analysis of cancer genomic data using cBioPortal (Cerami et al., 2012) suggests INPP4B is not frequently mutated in human cancer and there is no striking correlation between mutation in *PTEN* and *INPP4B*. However, significant co-reductions in INPP4B and PTEN expression have been noted in human breast, ovarian, and thyroid cancers (Fedele et al., 2010; Gewinner et al., 2009; Kofuji et al., 2015; Vo and Fruman, 2015). Further, in human prostate, INPP4B expression is regulated by androgen receptor signaling, and loss of both PTEN and INPP4B proteins is highly prevalent in castration-resistant, late-stage cancers (Rynkiewicz et al., 2015; Hodgson et al., 2011). Our results describing PTEN and INPP4B as PI(3,4)P_2 3- and 4-phosphatases, respectively, would provide a natural explanation for the impact of their combined loss in driving tumor progression.

The magnitude of EGF-stimulated PI(3,4)P_2 accumulation in PTEN-INPP4B-KO cells suggests the flux through 5-dephosphorylation of PI(3,4,5)P_3 is surprisingly large and that the steady-state levels of PI(3,4,5)P_3 are dynamically regulated by very fast rates of synthesis and degradation. Whether 5-dephosphorylation brings a quantitative or qualitative element to the class I PI3K-signaling network has been debated, with strong evidence now presented for both roles (Erneux et al., 2011; Hawkins and Stephens, 2016; Li and Marshall, 2015). The loss of INPP4B has been argued to drive increased activation of AKT through increased accumulation of PI(3,4)P_2 (Fedele et al., 2010; Gewinner et al., 2009), and several PI(3,4)P_2-selective signaling roles have also been described, for example, involving the putative PI(3,4)P_2 effectors TAPP1/2 (Dowler et al., 2000), TKS5 (Abram et al., 2003), SNX9 (Posor et al., 2013), and lamellipodin (Krause et al., 2004) in the regulation of receptor desensitization, invadopodia, endocytosis, and lamellipodia, respectively (Hawkins and Stephens, 2016; Li and Marshall, 2015). We present evidence that combined deletion of PTEN and INPP4B in Mcf10a cells significantly potentiates EGF-stimulated phosphorylation of AKT and also the formation of invadopodia, confirming that PI(3,4)P_2 synthesized in this context can signal through these routes. In contrast, however, a reduction in INPP4B expression in PTEN-null breast cancer lines has recently been argued to drive PI(3,4)P_2-dependent negative feedback, reducing activation of AKT and sensitizing cell growth to PI3Kβ inhibitors, an effect suggested to result from direct PI(3,4)P_2-mediated inhibition of class I PI3Ks (Reed and Shokat, 2017). Clearly, in these cells, the impact of reducing INPP4B expression on AKT phosphorylation was the opposite to that shown here for Mcf10a cells, an untransformed breast epithelial cell line (Figures 6A and 6B). Thus, the qualitative response of a given cell to a rise in PI(3,4)P_2 may depend exquisitely on the magnitude of this rise and the poise of the signaling pathway to respond to it.

Our results reveal an important and widespread role for PTEN as a PI(3,4)P_2 3-phosphatase. We think it is likely that this property of PTEN has been overlooked because of technical difficulties in measuring PI(3,4)P_2 in cellular extracts and the properties of recombinant PTEN in the in vitro assays constructed to

date. In the context of class I PI3K activation, loss of PTEN raises the levels of $PI(3,4,5)P_3$, increases flux through $PI(3,4,5)P_3$ 5-phosphatases, and slows dephosphorylation of the resulting $PI(3,4)P_2$. In some tissues, loss of PTEN alone is sufficient to drive huge accumulations of $PI(3,4)P_2$ (e.g., mouse prostate). In other cells, combined loss of both PTEN and INPP4B is required to see equivalent increases in $PI(3,4)P_2$ (e.g., Mcf10a cells). These very large accumulations of $PI(3,4)P_2$ will distort class I PI3K pathway signaling, through both a quantitative effect on the activation of common $PI(3,4)P_2$ and $PI(3,4,5)P_3$ effectors (e.g., AKT) and a signaling imbalance through the activation of $PI(3,4)P_2$-selective effectors (e.g., Tks5). The effects of this distortion will be context dependent, but, for PTEN-dependent tumorigenesis and metastasis, the contribution of $PI(3,4)P_2$-specific processes is clearly an area that now demands further investigation. The potential role of PTEN as a $PI(3,4)P_2$ phosphatase under normal physiological conditions, within both class I and class II PI3K-signaling pathways, is also a concept that now warrants further attention.

STAR★METHODS

Detailed methods are provided in the online version of this paper and include the following:

- KEY RESOURCES TABLE
- CONTACT FOR REAGENT AND RESOURCE SHARING
- EXPERIMENTAL MODEL AND SUBJECT DETAILS
  - Mice
  - Cell Lines
  - Human Tissue (Platelets)
- METHOD DETAILS
  - Preparation of Platelets for $PI(3,4)P_2$ Measurement
  - siRNA Suppression
  - Gene Editing of Mcf10a Cell Lines Using CRISPR/Cas9
  - Generation of Mcf10a Cells Stably Expressing Fluorescent Reporters
  - Western Blot
  - Lipid Extraction
  - Measurement of $PI(3,4)P_2$ and $PI(4,5)P_2$
  - Measurement of PI3P and PI4P
  - ^{33}P-Pi Labeling of Mcf10a Cells
  - In Vitro Phosphatase Assay
  - Invadopodia Assay
  - Live Cell Imaging of Mcf10a Cells
  - Mouse Prostate Dissection and Processing
  - Mouse Prostate Imaging
  - HPLC-MS Measurement of Mouse Prostate Phophoinositides
  - Mathematical Modeling
  - Statistics
  - Experimental Design

SUPPLEMENTAL INFORMATION

Supplemental Information includes seven figures and and three data files and can be found with this article online at https://doi.org/10.1016/j.molcel.2017.09.024.

AUTHOR CONTRIBUTIONS

Conceptualization, P.T.H. and L.R.S.; Methodology, A. Kielkowska, J.C., and T.S.; Software, V.Y.K., P.P., and N.L.N.; Investigation, M.M., A. Kielkowska, T.C., K.E.A., D.B., P.P., H.N., S.E., A. Koizumi, J.S., V.J., A.G., A.V., D.S., M.I., and N.L.N.; Resources, I.N., S.C., and T.S.; Writing – Original Draft, P.T.H., L.R.S., A. Kielkowska, M.M., and T.C.; Supervision, P.T.H., L.R.S., D.S., S.C., N.L.N., T.H., T.S., and S.F.; Funding Acquisition, P.T.H., L.R.S., S.B., T.S., T.H., and S.C.

ACKNOWLEDGMENTS

We would like to acknowledge Simon Walker for help with microscopy, Natalie Rynkiewicz for help in preparing and interpreting mouse prostate histology, and Marija Jankovic for help with model development. We would also like to thank Tom Durrant (University of Bristol) for platelet samples and Roger Williams/Glenn Masson (LMB, Cambridge) for the gift of recombinant PTEN. We thank Dr. A. Ramos-Montoya and Professor D.E. Neal from the former Uro-Oncology Research Group at the Cancer Research UK-Cambridge Research Institute for kindly transferring the PB-Cre4 mouse strain. N.L.N., L.R.S. and P.T.H. acknowledge financial support from the BBSRC (BB/J004456/1, BB/I003428/1, and BB/I003916/1) and The Wellcome Trust (WT085889MA). T.S. acknowledges financial support from the Japan Society for the Promotion of Science (JSPS) (KAKENHI grant JP15H05899) and the Japan Agency for Medical Research and Development (AMED) (grant 16gm0710002h0304). A. Kielkowska is in receipt of a BBSRC-CASE PhD studentship with GSK. S.C. is an employee and stakeholder of AstraZeneca. D.B. is an employee of AstraZeneca. S.B. is an employee and stakeholder of GSK. T.S. and H.N. are co-founders and stakeholders of Akita Lipid Technologies LCC.

Received: April 24, 2017
Revised: August 9, 2017
Accepted: September 18, 2017
Published: October 19, 2017

REFERENCES

Abram, C.L., Seals, D.F., Pass, I., Salinsky, D., Maurer, L., Roth, T.M., and Courtneidge, S.A. (2003). The adaptor protein fish associates with members of the ADAMs family and localizes to podosomes of Src-transformed cells. J. Biol. Chem. *278*, 16844–16851.

Anderson, K.E., Juvin, V., Clark, J., Stephens, L.R., and Hawkins, P.T. (2016). Investigating the effect of arachidonate supplementation on the phosphoinositide content of MCF10a breast epithelial cells. Adv. Biol. Regul. *62*, 18–24.

Balla, T. (2013). Phosphoinositides: tiny lipids with giant impact on cell regulation. Physiol. Rev. *93*, 1019–1137.

Cerami, E., Gao, J., Dogrusoz, U., Gross, B.E., Sumer, S.O., Aksoy, B.A., Jacobsen, A., Byrne, C.J., Heuer, M.L., Larsson, E., et al. (2012). The cBio cancer genomics portal: an open platform for exploring multidimensional cancer genomics data. Cancer Discov. *2*, 401–404.

Cheung, S.M.S., Kornelson, J.C., Al-Alwan, M., and Marshall, A.J. (2007). Regulation of phosphoinositide 3-kinase signaling by oxidants: hydrogen peroxide selectively enhances immunoreceptor-induced recruitment of phosphatidylinositol (3,4) bisphosphate-binding PH domain proteins. Cell. Signal. *19*, 902–912.

Clark, J., Anderson, K.E., Juvin, V., Smith, T.S., Karpe, F., Wakelam, M.J.O., Stephens, L.R., and Hawkins, P.T. (2011). Quantification of PtdInsP3 molecular species in cells and tissues by mass spectrometry. Nat. Methods *8*, 267–272.

Cuenca-López, M.D., Montero, J.C., Morales, J.C., Prat, A., Pandiella, A., and Ocana, A. (2014). Phospho-kinase profile of triple negative breast cancer and androgen receptor signaling. BMC Cancer *14*, 302.

Dalle Pezze, P., and Le Novère, N. (2017). SBpipe: a collection of pipelines for automating repetitive simulation and analysis tasks. BMC Syst. Biol. *11*, 46.

Dibble, C.C., and Cantley, L.C. (2015). Regulation of mTORC1 by PI3K signaling. Trends Cell Biol. *25*, 545–555.

Dowler, S., Currie, R.A., Campbell, D.G., Deak, M., Kular, G., Downes, C.P., and Alessi, D.R. (2000). Identification of pleckstrin-homology-domain-containing proteins with novel phosphoinositide-binding specificities. Biochem. J. *351*, 19–31.

Dyson, J.M., Fedele, C.G., Davies, E.M., Becanovic, J., and Mitchell, C.A. (2012). Phosphoinositide phosphatases: just as important as the kinases. Subcell. Biochem. *58*, 215–279.

Engelman, J.A., Luo, J., and Cantley, L.C. (2006). The evolution of phosphatidylinositol 3-kinases as regulators of growth and metabolism. Nat. Rev. Genet. *7*, 606–619.

Erneux, C., Edimo, W.E., Deneubourg, L., and Pirson, I. (2011). SHIP2 multiple functions: a balance between a negative control of PtdIns(3,4,5)P_3 level, a positive control of PtdIns(3,4)P_2 production, and intrinsic docking properties. J. Cell. Biochem. *112*, 2203–2209.

Fedele, C.G., Ooms, L.M., Ho, M., Vieusseux, J., O'Toole, S.A., Millar, E.K., Lopez-Knowles, E., Sriratana, A., Gurung, R., Baglietto, L., et al. (2010). Inositol polyphosphate 4-phosphatase II regulates PI3K/Akt signaling and is lost in human basal-like breast cancers. Proc. Natl. Acad. Sci. USA *107*, 22231–22236.

Ferron, M., and Vacher, J. (2006). Characterization of the murine Inpp4b gene and identification of a novel isoform. Gene *376*, 152–161.

Fruman, D.A., and Rommel, C. (2014). PI3K and cancer: lessons, challenges and opportunities. Nat. Rev. Drug Discov. *13*, 140–156.

Gewinner, C., Wang, Z.C., Richardson, A., Teruya-Feldstein, J., Etemadmoghadam, D., Bowtell, D., Barretina, J., Lin, W.M., Rameh, L., Salmena, L., et al. (2009). Evidence that inositol polyphosphate 4-phosphatase type II is a tumor suppressor that inhibits PI3K signaling. Cancer Cell *16*, 115–125.

Giuriato, S., Pesesse, X., Bodin, S., Sasaki, T., Viala, C., Marion, E., Penninger, J., Schurmans, S., Erneux, C., and Payrastre, B. (2003). SH2-containing inositol 5-phosphatases 1 and 2 in blood platelets: their interactions and roles in the control of phosphatidylinositol 3,4,5-trisphosphate levels. Biochem. J. *376*, 199–207.

Gligorijevic, B., Bergman, A., and Condeelis, J. (2014). Multiparametric classification links tumor microenvironments with tumor cell phenotype. PLoS Biol. *12*, e1001995.

Guillou, H., Stephens, L.R., and Hawkins, P.T. (2007). Quantitative measurement of phosphatidylinositol 3,4,5-trisphosphate. Methods Enzymol. *434*, 117–130.

Halet, G., Viard, P., and Carroll, J. (2008). Constitutive PtdIns(3,4,5)P3 synthesis promotes the development and survival of early mammalian embryos. Development *135*, 425–429.

Hawkins, P.T., and Stephens, L.R. (2015). PI3K signalling in inflammation. Biochim. Biophys. Acta *1851*, 882–897.

Hawkins, P.T., and Stephens, L.R. (2016). Emerging evidence of signalling roles for PI(3,4)P2 in Class I and II PI3K-regulated pathways. Biochem. Soc. Trans. *44*, 307–314.

Hawkins, P.T., Jackson, T.R., and Stephens, L.R. (1992). Platelet-derived growth factor stimulates synthesis of PtdIns(3,4,5)P3 by activating a PtdIns(4,5)P2 3-OH kinase. Nature *358*, 157–159.

Hodgson, M.C., Shao, L.J., Frolov, A., Li, R., Peterson, L.E., Ayala, G., Ittmann, M.M., Weigel, N.L., and Agoulnik, I.U. (2011). Decreased expression and androgen regulation of the tumor suppressor gene INPP4B in prostate cancer. Cancer Res. *71*, 572–582.

Hollander, M.C., Blumenthal, G.M., and Dennis, P.A. (2011). PTEN loss in the continuum of common cancers, rare syndromes and mouse models. Nat. Rev. Cancer *11*, 289–301.

Hoops, S., Sahle, S., Gauges, R., Lee, C., Pahle, J., Simus, N., Singhal, M., Xu, L., Mendes, P., and Kummer, U. (2006). COPASI—a COmplex PAthway SImulator. Bioinformatics *22*, 3067–3074.

Hucka, M., Finney, A., Sauro, H.M., Bolouri, H., Doyle, J.C., Kitano, H., Arkin, A.P., Bornstein, B.J., Bray, D., Cornish-Bowden, A., et al. (2003). The systems biology markup language (SBML): a medium for representation and exchange of biochemical network models. Bioinformatics *19*, 524–531.

Irvine, R.F., Letcher, A.J., and Dawson, R.M. (1984). Phosphatidylinositol-4,5-bisphosphate phosphodiesterase and phosphomonoesterase activities of rat brain. Some properties and possible control mechanisms. Biochem. J. *218*, 177–185.

Jackson, T.R., Stephens, L.R., and Hawkins, P.T. (1992). Receptor specificity of growth factor-stimulated synthesis of 3-phosphorylated inositol lipids in Swiss 3T3 cells. J. Biol. Chem. *267*, 16627–16636.

Jia, S., Liu, Z., Zhang, S., Liu, P., Zhang, L., Lee, S.H., Zhang, J., Signoretti, S., Loda, M., Roberts, T.M., and Zhao, J.J. (2008). Essential roles of PI(3)K-p110beta in cell growth, metabolism and tumorigenesis. Nature *454*, 776–779.

Juvin, V., Malek, M., Anderson, K.E., Dion, C., Chessa, T., Lecureuil, C., Ferguson, G.J., Cosulich, S., Hawkins, P.T., and Stephens, L.R. (2013). Signaling via class IA Phosphoinositide 3-kinases (PI3K) in human, breast-derived cell lines. PLoS ONE *8*, e75045.

Kielkowska, A., Niewczas, I., Anderson, K.E., Durrant, T.N., Clark, J., Stephens, L.R., and Hawkins, P.T. (2014). A new approach to measuring phosphoinositides in cells by mass spectrometry. Adv. Biol. Regul. *54*, 131–141.

Kiselev, V.Y., Juvin, V., Malek, M., Luscombe, N., Hawkins, P., Le Novère, N., and Stephens, L. (2015). Perturbations of PIP3 signalling trigger a global remodelling of mRNA landscape and reveal a transcriptional feedback loop. Nucleic Acids Res. *43*, 9663–9679.

Kofuji, S., Kimura, H., Nakanishi, H., Nanjo, H., Takasuga, S., Liu, H., Eguchi, S., Nakamura, R., Itoh, R., Ueno, N., et al. (2015). INPP4B is a PtdIns(3,4,5)P3 phosphatase that can act as a tumor suppressor. Cancer Discov. *5*, 730–739.

Krause, M., Leslie, J.D., Stewart, M., Lafuente, E.M., Valderrama, F., Jagannathan, R., Strasser, G.A., Rubinson, D.A., Liu, H., Way, M., et al. (2004). Lamellipodin, an Ena/VASP ligand, is implicated in the regulation of lamellipodial dynamics. Dev. Cell *7*, 571–583.

Le Novère, N., Bornstein, B., Broicher, A., Courtot, M., Donizelli, M., Dharuri, H., Li, L., Sauro, H., Schilstra, M., Shapiro, B., et al. (2006). BioModels Database: a free, centralized database of curated, published, quantitative kinetic models of biochemical and cellular systems. Nucleic Acids Res. *34*, 689–691.

Le Novère, N., Hucka, M., Mi, H., Moodie, S., Schreiber, F., Sorokin, A., Demir, E., Wegner, K., Aladjem, M.I., Wimalaratne, S.M., et al. (2009). The Systems Biology Graphical Notation. Nat. Biotechnol. *27*, 735–741.

Leong, H.S., Robertson, A.E., Stoletov, K., Leith, S.J., Chin, C.A., Chien, A.E., Hague, M.N., Ablack, A., Carmine-Simmen, K., McPherson, V.A., et al. (2014). Invadopodia are required for cancer cell extravasation and are a therapeutic target for metastasis. Cell Rep. *8*, 1558–1570.

Li, H., and Marshall, A.J. (2015). Phosphatidylinositol (3,4) bisphosphate-specific phosphatases and effector proteins: a distinct branch of PI3K signaling. Cell. Signal. *27*, 1789–1798.

Martin, K.H., Hayes, K.E., Walk, E.L., Ammer, A.G., Markwell, S.M., and Weed, S.A. (2012). Quantitative measurement of invadopodia-mediated extracellular matrix proteolysis in single and multicellular contexts. J. Vis. Exp. *66*, e4119.

Mayer, I.A., and Arteaga, C.L. (2016). The PI3K/AKT pathway as a target for cancer treatment. Annu. Rev. Med. *67*, 11–28.

McConnachie, G., Pass, I., Walker, S.M., and Downes, C.P. (2003). Interfacial kinetic analysis of the tumour suppressor phosphatase, PTEN: evidence for activation by anionic phospholipids. Biochem. J. *371*, 947–955.

Mendes, P., Hoops, S., Sahle, S., Gauges, R., Dada, J., and Kummer, U. (2009). Computational modeling of biochemical networks using COPASI. Methods Mol. Biol. *500*, 17–59.

Okkenhaug, K., Graupera, M., and Vanhaesebroeck, B. (2016). Targeting PI3K in cancer: impact on tumor cells, their protective stroma, angiogenesis, and immunotherapy. Cancer Discov. *6*, 1090–1105.

Ooms, L.M., Binge, L.C., Davies, E.M., Rahman, P., Conway, J.R.W., Gurung, R., Ferguson, D.T., Papa, A., Fedele, C.G., Vieusseux, J.L., et al. (2015). The

inositol polyphosphate 5-phosphatase PIPP regulates AKT1-dependent breast cancer growth and metastasis. Cancer Cell *28*, 155–169.

Posor, Y., Eichhorn-Gruenig, M., Puchkov, D., Schöneberg, J., Ullrich, A., Lampe, A., Müller, R., Zarbakhsh, S., Gulluni, F., Hirsch, E., et al. (2013). Spatiotemporal control of endocytosis by phosphatidylinositol-3,4-bisphosphate. Nature *499*, 233–237.

Posor, Y., Eichhorn-Grünig, M., and Haucke, V. (2015). Phosphoinositides in endocytosis. Biochim. Biophys. Acta *1851*, 794–804.

Ran, F.A., Hsu, P.D., Wright, J., Agarwala, V., Scott, D.A., and Zhang, F. (2013). Genome engineering using the CRISPR-Cas9 system. Nat. Protoc. *8*, 2281–2308.

Redfern, R.E., Redfern, D., Furgason, M.L.M., Munson, M., Ross, A.H., and Gericke, A. (2008). PTEN phosphatase selectively binds phosphoinositides and undergoes structural changes. Biochemistry *47*, 2162–2171.

Reed, D.E., and Shokat, K.M. (2017). INPP4B and PTEN loss leads to PI-3,4-P2 accumulation and inhibition of PI3K in TNBC. Mol. Cancer Res. *15*, 765–775.

Rittenhouse, S.E. (1996). Phosphoinositide 3-kinase activation and platelet function. Blood *88*, 4401–4414.

Rynkiewicz, N.K., Fedele, C.G., Chiam, K., Gupta, R., Kench, J.G., Ooms, L.M., McLean, C.A., Giles, G.G., Horvath, L.G., and Mitchell, C.A. (2015). INPP4B is highly expressed in prostate intermediate cells and its loss of expression in prostate carcinoma predicts for recurrence and poor long term survival. Prostate *75*, 92–102.

Sasaki, J., Kofuji, S., Itoh, R., Momiyama, T., Takayama, K., Murakami, H., Chida, S., Tsuya, Y., Takasuga, S., Eguchi, S., et al. (2010). The PtdIns(3,4)P(2) phosphatase INPP4A is a suppressor of excitotoxic neuronal death. Nature *465*, 497–501.

Seals, D.F., Azucena, E.F., Jr., Pass, I., Tesfay, L., Gordon, R., Woodrow, M., Resau, J.H., and Courtneidge, S.A. (2005). The adaptor protein Tks5/Fish is required for podosome formation and function, and for the protease-driven invasion of cancer cells. Cancer Cell *7*, 155–165.

Sharma, V.P., Eddy, R., Entenberg, D., Kai, M., Gertler, F.B., and Condeelis, J. (2013). Tks5 and SHIP2 regulate invadopodium maturation, but not initiation, in breast carcinoma cells. Curr. Biol. *23*, 2079–2089.

Stephens, L.R., Hughes, K.T., and Irvine, R.F. (1991). Pathway of phosphatidylinositol(3,4,5)-trisphosphate synthesis in activated neutrophils. Nature *351*, 33–39.

Stephens, L.R., Jackson, T.R., and Hawkins, P.T. (1993). Agonist-stimulated synthesis of phosphatidylinositol(3,4,5)-trisphosphate: a new intracellular signalling system. Biochim. Biophys. Acta *1179*, 27–75.

Thorpe, L.M., Yuzugullu, H., and Zhao, J.J. (2015). PI3K in cancer: divergent roles of isoforms, modes of activation and therapeutic targeting. Nat. Rev. Cancer *15*, 7–24.

Trotman, L.C., Niki, M., Dotan, Z.A., Koutcher, J.A., Di Cristofano, A., Xiao, A., Khoo, A.S., Roy-Burman, P., Greenberg, N.M., Van Dyke, T., et al. (2003). Pten dose dictates cancer progression in the prostate. PLoS Biol. *1*, E59.

Vanhaesebroeck, B., Guillermet-Guibert, J., Graupera, M., and Bilanges, B. (2010). The emerging mechanisms of isoform-specific PI3K signalling. Nat. Rev. Mol. Cell Biol. *11*, 329–341.

Vo, T.T.T., and Fruman, D.A. (2015). INPP4B is a tumor suppressor in the context of PTEN deficiency. Cancer Discov. *5*, 697–700.

Walker, S.M., Downes, C.P., and Leslie, N.R. (2001). TPIP: a novel phosphoinositide 3-phosphatase. Biochem. J. *360*, 277–283.

Wang, H., Huang, M., Zhang, D.Y., and Zhang, F. (2011). Global profiling of signaling networks: study of breast cancer stem cells and potential regulation. Oncologist *16*, 966–979.

Wu, X., Wu, J., Huang, J., Powell, W.C., Zhang, J., Matusik, R.J., Sangiorgi, F.O., Maxson, R.E., Sucov, H.M., and Roy-Burman, P. (2001). Generation of a prostate epithelial cell-specific Cre transgenic mouse model for tissue-specific gene ablation. Mech. Dev. *101*, 61–69.

Wu, G., Xing, M., Mambo, E., Huang, X., Liu, J., Guo, Z., Chatterjee, A., Goldenberg, D., Gollin, S.M., Sukumar, S., et al. (2005). Somatic mutation and gain of copy number of PIK3CA in human breast cancer. Breast Cancer Res. *7*, R609–R616.

Yamaguchi, H., Yoshida, S., Muroi, E., Yoshida, N., Kawamura, M., Kouchi, Z., Nakamura, Y., Sakai, R., and Fukami, K. (2011). Phosphoinositide 3-kinase signaling pathway mediated by p110α regulates invadopodia formation. J. Cell Biol. *193*, 1275–1288.

STAR★METHODS

KEY RESOURCES TABLE

REAGENT or RESOURCE	SOURCE	IDENTIFIER
Antibodies		
Anti-phospho-Akt-S473	Cell Signaling	D9E, 4060; RRID: AB_2315049
Anti-phospho-Akt-T308	Cell Signaling	5106; RRID: AB_836861
Anti-β-COP	Babraham Institute	Nick Ktistakis
Anti-βactin	Abcam	Ab6276; RRID: AB_2223210
Anti-INPP4A	Santa Cruz	Sc-12314; RRID: AB_2126009
Anti-INPP4B	Santa Cruz	Sc-12318; RRID: AB_2126126
Anti-SHIP1	Cell Signaling	2728; RRID: AB_2126244
Anti-PTEN	Cell Signaling	9188; RRID: AB_2253290
Anti-EGFR	Cell Signaling	4267; RRID: AB_2246311
Anti-phospho-EGFR	Cell Signaling	3777; RRID: AB_2096270
Anti-Cortactin	Abcam	Ab33333; RRID: AB_731713
Biotinylated anti-PI(3,4)P2	Echelon Biosciences	z-B034b; RRID: AB_427214
Alexa 568 goat anti-mouse	Life Technologies	A11004; RRID: AB_2534072
Alexa 568 goat anti-Rabbit	Life Technologies	A11036; RRID: AB_10563566
Alexa 647 goat anti-mouse	Life Technologies	A21235; RRID: AB_2535804
Alexa 647 goat anti-Rabbit	Life Technologies	A21244; RRID: AB_10562581
Steptavidin-Alexa 647	Life Technologies	S32357
IRdye 680 goat anti-Rabbit IgG	LI-COR	926-68071; RRID: AB_10956166
IRdye 800 goat anti-mouse IgG	LI-COR	926-32210; RRID: AB_621842
Goat anti-mouse antibody HRP	Bio-Rad	170-6516; RRID: AB_11125547
Goat anti-Rabbit antibody HRP	Bio-Rad	170-6515; RRID: AB_11125142
Anti-SHIP2	Cell Signaling	2730; RRID: AB_659982
Biological Samples		
Human Platelets	Dr Ingeborg Hers	University of Bristol
Chemicals, Peptides, and Recombinant Proteins		
TMS-diazomethane	Sigma-Aldrich	362832
Fatty acid free BSA	Sigma-Aldrich	A7906
Dulbecco’s phosphate buffered saline	Sigma-Aldrich	D8537
Human insulin	Sigma-Aldrich	I9278
Human EGF	Sigma-Aldrich	E9644
Hydrocortisone	Sigma-Aldrich	H0888
PBS10x	Life Technologies	70011-036
DMEM/F12	Life Technologies	31330-038
RPMI-1640	Sigma-Aldrich	R8758
Insulin-Transferrin-Selenium	Thermo Fisher Scientific	41400045
Dihydrotestosterone	Sigma-Aldrich	D-073-1ML
Fetal Bovine Serum	Thermo Fisher Scientific	10270106
Distilled water	Life Technologies	15230-089
rhTGF-β1	R&D Systems	240-B
Gelatin from pig skin- Oregon Green 488	Fisher Scientific	11594856
[33]P-γATP	PerkinElmer	NEG602H250UC
BYL719	Biochem Inhibitor	A-1214

(*Continued on next page*)

Continued

REAGENT or RESOURCE	SOURCE	IDENTIFIER
d6-C18:0/C20:4-PI(3,4,5)P3	Synthesized by the Biological Chemistry Department in Babraham Institute	Contact Jonathan Clark
1-heptadecanoyl-2-hexadecanoyl-sn-glycero-3- (phosphoinositol 3,4,5-trisphosphate) (C17:0/C16:0-PI(3,4,5)P3)	Synthesized by the Biological Chemistry Department in Babraham Institute	Contact Jonathan Clark
C17:0/C16:0-PI	Synthesized by the Biological Chemistry Department in Babraham Institute	Contact Jonathan Clark
d6- C18:0/C20:4-PI(3)P	Synthesized by the Biological Chemistry Department in Babraham Institute	Contact Jonathan Clark
d6-C18:0/C20:4-PI(4)P	Synthesized by the Biological Chemistry Department in Babraham Institute	Contact Jonathan Clark
d6-C18:0/C20:4-PI(3,4)P2	Synthesized by the Biological Chemistry Department in Babraham Institute	Contact Jonathan Clark
d6-C18:0/C20:4- PI(4,5)P2	Synthesized by the Biological Chemistry Department in Babraham Institute	Contact Jonathan Clark
C18:0/C20:4-PI(3,4)P2	Synthesized by the Biological Chemistry Department in Babraham Institute	Contact Jonathan Clark
C18:0/C20:4 PI(3,5)P2	Synthesized by the Biological Chemistry Department in Babraham Institute	Contact Jonathan Clark
C18:0/C20:4 PI(4,5)P2	Synthesized by the Biological Chemistry Department in Babraham Institute	Contact Jonathan Clark
C18:0/C20:4 PI(3,4,5)P3	Synthesized by the Biological Chemistry Department in Babraham Institute	Contact Jonathan Clark
C17:0/C20:4-PI	Avanti Polar Lipids	LM-1502
Horse serum	PAA	B15-021
Penicillin/streptomycin	Life Technologies	15140-122
CS-FBS	Life Technologies	12676-029
Cholera toxin	Sigma-Aldrich	C8052
DharamaFECT1	GE Dharmacon	T-2001-04
Optimem	Life Technologies	31985-047
Lipofectamine 2000	Invitrogen	11668-019
Polybrene used at 4 μg/mL	Sigma-Aldrich	TR-1003-G
Tris	Melford	B2005
NaCl	VWR Chemicals	27810.295
EDTA	Sigma-Aldrich	E5134
EGTA	Sigma-Aldrich	E4378
Triton	Sigma-Aldrich	T9284
Anti-protease leupeptin	Sigma-Aldrich	L8511
Anti-protease Aprotinin	Sigma-Aldrich	A1153
Anti-protease antipain	Sigma-Aldrich	A6191
Anti-protease pepstatin	Sigma-Aldrich	P5318
PMSF	Sigma-Aldrich	78830
Na4P2O7	Sigma-Aldrich	P8010
β- glycerolphosphate	Calbiochem	35675
Na3VO4	Sigma-Aldrich	S6508
NaF	Sigma-Aldrich	S7920
PVDF membranes (immobilon P)	Millipore	IPVH00010
DTT	Melford	MB1015
Tris-HCl	Sigma-Aldrich	T3253
Glycerol	Invitrogen	15514-011

(*Continued on next page*)

Continued

REAGENT or RESOURCE	SOURCE	IDENTIFIER
Bromophenol Blue	Sigma-Aldrich	B8026
Methanol	Romil	H410
Chloroform	Romil	H140
Acetone	VWR chemicals	20066.330
Dimethyl sulphide	Sigma-Aldrich	34869
Formic acid	Fisher Scientific	F/1900/PB08
Acetonitrile	Romil	M050
HEPES	Sigma-Aldrich	H3375
MgCl2	VWR Chemicals	25108.295
Protease inhibitor cocktail	Roche	11836170001
Bovin Serum Albumin	Sigma-Aldrich	A7906
KCl	VWR Chemicals	26764.260
Trichloroacetic acid 6.1N	Sigma-Aldrich	T0699
Embedding medium	Thermo Scientific	1310
Mayer's hematoxylin solution	Sigma-Aldrich	SLBP6175V
Eosin Y solution	Sigma-Aldrich	SLBP1949V
Critical Commercial Assays		
AMAXA nucleofection system	Lonza	Kit T
Lasky ozone generator	AirTree Ozon technology	C-L010-DT
Deposited Data		
Original Images deposited at Mendeley Data	Mendeley Data	https://doi.org/10.17632/tnj6m88k6w.1
Experimental Models: Cell Lines		
$PTEN^{-/-}$ and Parental Mcf10a	Horizon Discovery	HD101-006
Experimental Models: Organisms/Strains		
PB-Cre4 mice	JAX	Strain 026662
PTENloxP/loxP mice	JAX	Strain 004597
'WT' ($PTEN^{loxP/loxP}$, $PbCre^{-/-}$) mice	This paper	N/A
'PTEN-KO' ($PTEN^{loxP/loxP}$, $PbCre^{+/-}$) mice	This paper	N/A
$INPP4B^{-/-}$ mice	Kofuji et al., 2015	N/A
BPH-1	DSMZ	ACC143
DU-145	Dr Scholes	ex Tenovus Institute
PC-3	ATCC	CRL 1435
EVSA-T	DSMZ	ACC 433
LnCAP-95	Dr Meeker	John Hopkins University
T47D	ATCC	HTB 133
LnCAP	ATCC	CRL 1740
CAL-120	DSMZ	ACC 459
BT-549	ATCC	HTB 122
MDA-MB-436	ATCC	HTB 130
HCC-70	ATCC	CRL 2315
HCC-1937	ATCC	CRL 2336
MDA-MB-157	ATCC	HTB 24
HCC-1187	ATCC	CRL 2322
Oligonucleotides		
INPP4B knockout sgRNA	designed by https://chopchop.rc.fas.harvard.edu	5′-GATCTCCGTAATCCACCCCG-3′
SHIP2 knockout sgRNA	designed by https://chopchop.rc.fas.harvard.edu	5′- GTGCAGGCCTTTGAGGTACA-3′

(*Continued on next page*)

Continued		
REAGENT or RESOURCE	SOURCE	IDENTIFIER
Recombinant DNA		
pSpCas9(BB)-2A-GFP	Addgene	48138
pMIGR1-mCherry-PH-TAPP	This paper	N/A
GFP-GRP1-PH domain	Halet et al., 2008	N/A
PLVX-IRES-Puro Vector	Clontech	632183
Software and Algorithms		
FIJI	NIH	N/A
Prism	GraphPad	N/A
Imaris	Bitplane	N/A
COPASI	Hoops et al., 2006	N/A

CONTACT FOR REAGENT AND RESOURCE SHARING

Further information and requests for resources and reagents should be directed to and will be fulfilled by the Lead Contact, Phillip Hawkins (phillip.hawkins@babraham.ac.uk).

EXPERIMENTAL MODEL AND SUBJECT DETAILS

Mice

PB-Cre4 mice (Wu et al., 2001) and PTEN$^{loxP/loxP}$ mice (Trotman et al., 2003) have been described previously. PbCre4 mice and PTEN$^{loxP/loxP}$ mice were interbred to generate ‘WT’ (PTEN$^{loxP/loxP}$, PbCre$^{-/-}$) and ‘PTEN-KO’ (PTEN$^{loxP/loxP}$, PbCre$^{+/-}$) mice and backcrossed to the C57BL/6J strain for at least 4 generations; these mice were housed in the Biological Support Unit at The Babraham Institute. INPP4B$^{-/-}$ mice have been described previously (Kofuji et al., 2015) and were interbred with PTEN-KO mice to generate ‘PTEN-INPP4B-KO’ (PTEN$^{loxP/loxP}$, PbCre$^{+/-}$, INPP4B$^{-/-}$) mice; these mice were housed in the Akita University Animal House.

Sample Size Estimation

No estimation of simple size was performed as sample sizes were not chosen based on pre-specified effect size. Instead, multiple independent experiments were carried out using several biological replicates specified in the legends to figures.

How Subjects/Samples Were Allocated to Experimental Groups

Prostates from several age-matched mice of identical genotype were analyzed.

Gender of Subjects or Animals

Male mice were used.

Health/Immune Status

The animals were kept under SPF conditions and the animal facilities where the mice were kept were regularly checked for standard pathogens. Health reports can be provided upon request.

Whether Subjects Were Involved in Previous Procedures

Prostates were prepared from mice not subject to any previous experimentation.

Whether the Subject Is Drug or Test Naive

Mice used for all experiments were naive. No drug tests were done.

Husbandry Conditions of Experimental Animals

All animal experiments at The Babraham Insitute were reviewed and approved by The Animal Welfare and Ethics Review Body and performed under Home Office Project license PPL 70/8100. Animal experiments in Akita were reviewed and approved by the Akita University Institutional Committee for Animal Studies, Akita University. The mice were looked after by professional caretakers. Every animal was checked daily.

Housing Conditions of Experimental Animals

Animals housed in the Biological Support Unit at the Babraham Institute were kept under specific pathogen–free conditions. Mice in the animal facility in Akita were kept in groups of up to six animals in standard IVC cages of 524 cm^2 containing bedding and nesting material. Food and water was provided ad libitum. The light cycle ran from 6 am to 6 pm.

Cell Lines

Mcf10a cells are non-transformed human female breast epithelial cells. PTEN$^{-/-}$ Mcf10a cell lines were generated by targeted homologous recombination and were obtained from Horizon Discovery Ltd together with their parental cell lines. All Mcf10a cell lines

were maintained at 37°C with 5% CO_2 in DMEM/F12 supplemented with 5% horse serum, 10 ng/mL EGF, 10 μg/mL insulin, 0.1 μg/mL cholera toxin, 0.5 μg/mL hydrocortisone, 1% w/v penicillin/streptomycin (complete medium). Starvation medium consisted of DMEM/F12 supplemented with 1% charcoal/dextran treated fetal bovine serum, 0.1 μg/mL cholera toxin, 0.5 μg/mL hydrocortisone, 1% P/S.

Human prostate cancer cells (DU-145, BPH-1, LNCaP, LNCaP 95 and PC-3) and breast cancer cells (T-47D, EVSA-T, CAL-120, BT-549, MDA-MB-436, HCC70, HCC-1187, HCC-1937, MDA-MB-157) were obtained from the AstraZeneca cell bank and had been previously authenticated using DNA fingerprinting short tandem repeat assays. All revived cells were used within 10 passages and cultured at 37°C with 5% CO2 for less than 2 months. Benign prostatic hyperplasia epithelial cell line BPH-1 was cultured in RPMI-1640 supplemented with 20% FBS, 10 μg/mL insulin, 6.7 ng/mL sodium selenite, 5.5 μg/mL transferrin, 0.5 nM dihydrotestosterone and 1% w/v penicillin/streptomycin. The remaining prostate and breast cancer cell lines were grown in RPMI-1640 with 10% FBS and 1% w/v penicillin/streptomycin.

Human Tissue (Platelets)

Venous blood was obtained from a healthy female human volunteer with the approval of the local research ethics committee at the University of Bristol, UK. The donor provided written informed consent, and reported as not having taken medication in the 14 days prior to donation. Blood was drawn into 4% trisodium citrate (1:9, v/v), and acidified with acidic citrate dextrose (1:7, v/v; 120 mM sodium citrate, 110 mM glucose, 80 mM citric acid). Platelet-rich plasma (PRP) was obtained by centrifugation of the blood at 180xg for 17 min at room temperature. Platelets were subsequently pelleted by centrifugation of the PRP at 650 x g for 10 min at room temperature in the presence of 10 μM indomethacin and 0.02 U/mL apyrase (grade VII). Platelets were resuspended at 4×10^8/mL in HEPES–Tyrode buffer (145 mM NaCl, 3 mM KCl, 0.5 mM Na_2HPO_4, 1 mM $MgS0_4.7H_2O$, 10 mM HEPES, pH 7.2) supplemented with 0.1% [w/v] D-glucose, 10 μM indomethacin and 0.02 U/mL apyrase, and allowed to rest at 30°C for 30 min prior to experimentation.

METHOD DETAILS

Preparation of Platelets for $PI(3,4)P_2$ Measurement

1×10^8 platelets were preincubated with DMSO or 100 nM Wortmannin for 10 min, before treatment with HEPES–Tyrode buffer or 0.5 U/mL thrombin for 3 min under stirring at 1200 r.p.m using a Chronolog 490-4D aggregometer at 37°C (Labmedics, Abingdon-on-Thames, UK). Treatment was stopped by the addition of 750 μl ice-cold 1 M HCl and samples were centrifuged at 12000 x g for 10 min at 4°C. Resulting pellets were frozen until lipid extraction.

siRNA Suppression

1.6×10^5 cells were seeded per 35 mm dish, and were subject to reverse transfection (using transfection agent DharamaFECT1) with a pool of 4 different siRNA (40 nM per target; ON-Target-plus pooled siRNA.) in Optimem and 10% complete medium, according to manufacturer's instructions. Media was changed after 16 hr and replaced with complete medium for 32 hr, after which cells were washed with dPBS and maintained in starvation media for 16 hr. Cells were stimulated with 10 ng/mL of EGF for the indicated times. Where indicated, cells were pre-incubated with inhibitors for 20 min prior to EGF stimulation. Stimulations were terminated by aspiration of media and washing with ice-cold PBS, prior to processing of the cells for lipid or western blot analysis as described below.

Gene Editing of Mcf10a Cell Lines Using CRISPR/Cas9

sgRNAs were designed using https://chopchop.rc.fas.harvard.edu/ or http://crispr.mit.edu/ and cloned into all-in-one pSpCas9(BB)-2A-GFP plasmid, plasmid as described previously (Ran et al., 2013). To generate an INPP4B knockout, sgRNA 5′- GATCTCCGTAATCCACCCCG-3′ targeting exon 7 was used. SHIP2 knockout was generated using sgRNA 5′-GTGCAGGCCTTT GAGGTACA-3′ directed against exon 8. Mcf10a cells were transfected with 4 μg DNA using the AMAXA nucleofection system. After 24-48 hr, GFP positive cells were FACS sorted and seeded at the density of up to 1 cell per well in a 96 well plate using a conditioned medium (1:1 mix of fresh Mcf10a medium and conditioned medium harvested after 3 days in culture with Mcf10a cells and 0.45 μm filtered). Single clones were picked after 7 days, expanded, and analyzed for loss of protein by western blot using anti-INPP4B and anti-SHIP2 antibodies.

Generation of Mcf10a Cells Stably Expressing Fluorescent Reporters

The GFP-PH-GRP1 domain construct was kindly provided by Guillaume Halet (Halet et al., 2008). This construct incorporates a nuclear export signal resulting in the exclusion of GFP-PH-GRP1 from the nucleus, and was subcloned into PLVX-IRES-Puro Vector. The generation of Lentivirus as well as the transduction of Mcf10A cells were performed according to the manufacturer's guidelines.

mCherry-PH-TAPP1 expressing Mcf10a cells were generated using the isolated PH domain of TAPP1 cloned into the retroviral vector pMIGR1, previously modified to introduce mCherry fluorescent protein cDNA upstream of the multiple cloning site (pMIGR1-mCherry). Retrovirus was generated by transfecting 10 μg pMIGR1-mCherry-PH-TAPP into amphotropic phoenix cells (maintained in DMEM supplemented with 10% FBS, 1% penicillin/streptomycin in 37°C humidified incubator) using lipofectamine 2000, according to the manufacturer instructions. Following 24 hr incubation, media was replaced with complete Mcf10a medium

and incubated at 32°C for a further 24 hr. Retroviral containing media was then collected and passed through a 0.45 μm filter, before adding to WT or PTEN$^{-/-}$ Mcf10a cells, as indicated, cultured in 6 well dishes. Cells were incubated at 32°C for a further 4 hr in the presence of 4 μg/mL polybrene before transferring to 37°C. Cells were expended and washed several times in complete Mcf10a media before a mixed population of mCherry-PH-TAPP1 expressing cells were used for image analysis as described below.

Western Blot

Mcf10a

Cells were scraped and lysed in 150 μL of ice-cold lysis buffer (20 mM Tris, pH 7.5; 150 mM NaCl; 1 mM EDTA, pH 7.5; 1 mM EGTA, pH 7.5; 0.1% v/v Triton X-100 supplemented with anti-proteases: 10 μg/mL leupeptin, 10 μg/mL aprotinin, 10 μg/mL antipain, 10 μg/mL pepstatin A, 0.1 mM PMSF and anti-phosphatases: 2.5 mM $Na_4P_2O_7$, 5 mM β- glycerophosphate, 1 mM Na_3VO_4, 25 mM NaF). After 30 min solubilisation at 4°C with agitation, lysates were centrifuged (15,000 x g, 10 min, 4°C) and the supernatants collected and diluted in SDS-PAGE sample buffer. Proteins (45 μg/well, or 15 μg/well, where indicated) were resolved on a SDS-PAGE gel, transferred to PVDF membranes and blotted with the indicated primary antibodies at 4°C overnight. They were then washed in TBS (40 mM Tris/HCl, pH 8.0, 22°C; 0.14 M, NaCl) containing 0.1% v/v Tween 20 and incubated with a mix of Infrared Dye coupled secondary antibodies. The membranes were imaged with the Li-Cor Odyssey Infrared Imaging System using the 700 nm and 800 nm channels. Signals were quantified using the Image Studio software. Alternatively, membranes were washed and incubated with HRP-conjugated secondary antibodies and signals detected using the ECL detection system. Signals from the HRP-incubated membranes were quantified using Aida software.

Mouse Tissues

Tissues were pulverized under a continuous flow of $N_{2(l)}$. 1x reducing SDS sample buffer (0.1 M DTT, 40 mM Tris-HCl pH 6.7, 12.5% glycerol, 0.003% Bromophenol Blue) was pre-warmed to 70°C and 750 μL of sample buffer was added per 50 mg tissue to yield an approximate final protein concentration of 4 mg/mL. Lysates were homogenized by vortexing for 15 s followed by a sequential syringe step through a 21G needle (3x), followed by a 23G needle (3x). Proteins were denatured by boiling at 95°C for 5 min. Lysates were cleared by centrifugation for 5 min at 20,238 x g, after which the syringe and centrifugation steps were repeated. Proteins were resolved by SDS-PAGE (20 μg estimated total protein per lane) and immunoblotted for the indicated antibodies.

Lipid Extraction

750,000 Mcf10a cells grown on a 35 mm dish were killed in 750 μL ice-cold 1 M HCL, then scraped and collected into an Eppendorf tube. Each sample was then split into three separate 2 mL polypropylene Eppendorf tubes; 250 μL for PI, PIP, PIP_2, PIP_3 measurement, 250 μL for PI(3,4)P_2/PI(4,5)P_2 measurement, and the remaining cells were kept for analysis by western blot. Cells were pelleted in a microfuge (15,000 x g, 10 min at 4°C), the supernatant removed and cell pellets either processed immediately or snap-frozen in liquid nitrogen and stored at −80°C for up to two weeks.

For human prostate and breast cancer cell lines, 250,000 cells were seeded into 35 mm dishes and grown in the medium optimal for each cell line for 32h. Cells were then starved for 16h by replacing the medium with starvation medium – a phenol red-free RPMI 1640 supplemented with 2mM glutamax. Following stimulation and / or inhibition with appropriate reagents, medium was removed by aspiration and cells killed with 750 μl ice-cold 1M HCL. Cells were then scraped, collected into Eppendorf tubes, pelleted, and snap-frozen, as described above.

920 μL of a solvent mixture containing 2:1:0.79 (v/v) MeOH:$CHCl_3$:$H_2O_{(acidic)}$ was added to the cell pellets and the mixture vortexed thoroughly for 10 s. Relevant internal standards were then added:10 ng C17:0/C16:0-PIP_3, 100 ng C17:0/C16:0-PI, 250 ng d6-C18:0/C20:4-PI(4,5)P_2 for routine analysis of PI, PIP, PIP_2 and PIP_3; 50 ng C17:0/C20:4 PI, 50 ng d6- C18:0/C20:4-PI(3,4)P_2, 250 ng d6-C18:0/C20:4-PI(4,5)P_2 for routine analysis of PI, PI(3,4)P_2 and PI(4,5)P_2. Lipids were then extracted using an acidified Folch phase partition and derivatised with TMS-diazomethane (Clark et al., 2011).

Molecules derived from PI, PIP, PIP_2, and PIP_3 were measured by HPLC-MS (Kielkowska et al., 2014). Response ratios were calculated for the endogenous species of these lipids divided by their relevant C17:0/C16:0 internal standard. We routinely analyzed 5 molecular species of these lipids but present here data for the C38:4 species only, to align with data presented for the C38:4 species of PI(3,4)P_2 and PI(4,5)P_2 (see below). The C38:4 species of PIP_2 and PIP_3 represent approx. 10%–15% of the total species of these lipids in Mcf10a cells and all species behave very similarly upon stimulation with EGF (Anderson et al., 2016). In some experiments, absolute amounts of C38:4 PI(3,4,5)P_3 were generated by reference to standard curves previously generated for this molecular species (Kielkowska et al., 2014). Three technical replicates were routinely analyzed for each experiment and, unless stated otherwise, data are presented as means SD of three biological replicates.

Molecules derived from PI, PI(3,4)P_2 and PI(4,5)P_2 were analyzed by a new HPLC-MS method, see below. Response ratios were calculated for the endogenous C38:4 species of these lipids divided by their relevant d6-labeled internal standard. In some experiments, absolute amounts of these lipids were generated by reference to standard curves (Figure S1). Three technical replicates were routinely analyzed for each experiment and, unless stated otherwise, data are presented as means SD of three biological replicates.

Measurement of PI(3,4)P_2 and PI(4,5)P_2

Sample Preparation

Lipids were extracted and derivatized with TMS-diazomethane; we added 2 M TMS-diazomethane in hexane (50 μl) to lipid extracts prepared as described above (approx I mL of 'lower phase'), to give a yellow solution, and then allowed the reaction to proceed for 10 min, RT. We quenched reactions with glacial acetic acid (6 μl), which removed the sample's yellow color (this reaction releases N_2 gas, thus care should be taken). We added post-derivatisation wash solution (700 μl) to the organic solution, and mixed the samples, which we then centrifuged (5000 rpm, 3 min), and collected the resultant lower phase. We repeated the wash step, and then added MeOH:H_2O (9:1 v:v, 100 μl) to the final collected lower phase. The samples were then dried down under a stream of nitrogen at room temperature without warming until dry. 160 μL methanol was then added and sonicated briefly, then left at RT for about 30 min prior to ozonolysis. A C-Lasky ozone generator was used in the following procedure. The unit was set to use air as the oxygen source at a flow rate of 4 dm^3/min and the flow was split after the ozone generator so that approximately 75 to 90% of the flow went to an ozone destruction unit and the remaining fraction was used to bubble through the solution containing the samples. The power level on the ozone generator was set to about 60% of the maximum level. The Ozonolysis procedure started by placing the glass sample vials containing the methylated lipid solutions in an aluminum block which was cooled in an acetone/dry ice bath to a temperature of about −70°C. Ozone was then bubbled through the solutions for 5 min. Dimethyl sulphide (2 μl) was then added to each sample and then allowed to warm up to RT. Water (40 μl) was then added to each sample which was then ready to be submitted for analysis by UPLC, using the following conditions for PI(3,4P)2/PI(4,5P)2 separation:

Solvent A: Water, 0.1% formic acid
Solvent B: (40% acetonitrile/60% methanol), 0.1% formic acid
Column temperature: 60°C
Injection volume 45 μl

Gradient:

Time (min)	Flow rate (mL/min)	%A	%B	Curve
0	0.4	30	70	
18.99	0.4	30	70	6
19.00	0.4	20	80	1
22.00	0.4	20	80	6
24.00	0.4	0	100	6
29.00	0.4	0	100	6
30.00	0.4	30	70	6
35.00	0.4	30	70	6

QTRAP4000 Mass spectrometer parameters:
Positive mode, Q1 and Q3 unit resolution

CUR	20	CAD	Medium
IS	4500	DP	100
TEM	300	EP	10
GS1	18	CE	35
GS2	20	CXP	10
ihe	ON		

Turbo Spray source

Transitions:

Analyte	Q1 mass (Da)	Q3 mass (Da)	Dwell (ms)
d6-SA-PIP$_2$-Aldehyde product	935.4	445.3	50
Endogenous SA PIP$_2$-Aldehyde product	929.4	439.3	50
Endogenous SA PI-Aldehyde product	713.38	439.3	50
17:0-20:4 PI-Aldehyde product	699.4	425.3	50

Measurement of PI3P and PI4P

Precisely the same conditions were used for the measurement of PI(3,4)P2 and PI(4,5)P2 described above, except for the following UPLC-MS condtions:

UPLC Conditions for PI3P/PI4P Separation

Additional sample preparation: Take 40 µl of sample prepared as above and add to 200µl 70% methanol /30% water. Inject 5 µl

Column: ACE Excel 2 C18-Amide, 150 mm x 0.5 mm
Solvent A: Water, 0.1% formic acid
Solvent B: (40% acetonitrile/60% methanol), 0.1% formic acid
Column temperature: 60°C

Gradient:

Time (min)	Flow rate (ul/min)	%A	%B	Curve
0	10	30	70	
5	10	30	70	6
10	10	22	78	1
35	10	22	78	6
36	10	0	100	6
45	10	0	100	6
46	10	30	70	6
60	10	30	70	6

Mass spectrometer parameters as above.
Transitions:

Analyte	Q1 mass (Da)	Q3 mass (Da)	Dwell (ms)
d6-SA-PIP-Aldehyde product	827.4	445.3	50

[33]P-Pi Labeling of Mcf10a Cells

We added 0.1 mL of 1.5 M NaCl to 1 mL of ^{33}P-Pi and then diluted this mixture into a phosphate-depleted medium (GIBCO) and supplemented with (20 mM HEPES, 1% Dialysed FBS, 500 ng/mL hydrocortisone, 100 ng/mL cholera toxin) to reach a final concentration of 250 µC/mL. After siRNA transfection, as described above, cells were starved for 16 hr, then washed twice with phosphate-depleted medium before adding the ^{33}P-Pi–containing medium for 90 min. Then we stimulated the cells with 10 ng/mL hEGF. We aspirated the medium then stopped the reaction with ice-cold 1 M HCl. We extracted and deacylated the lipids, and analyzed the glycerophoinositides by strong anion-exchange chromatography (Guillou et al., 2007).

In Vitro Phosphatase Assay

Mcf10a cells (1x10^6) were seeded in 10 cm tissue culture dishes and grown for 64 hr, then washed twice with ice-cold PBS prior to lysis with 1 mL of lysis buffer (10 mM Tris pH7.4, 1.5 mM $MgCl_2$, 5 mM KCl, 1 mM DTT, anti-proteases (1 tablet Roche inhibitor cocktail

per 50 mL lysis buffer)) on ice. Cells were then scraped on ice, collected in 2 mL safe lock Eppendorf tubes, vortexed, and kept on ice for 5 min. The lysate was sonicated on ice (4 × 10 s; probe sonication) and then ultracentrifuged (40,000 x g for 30 min at 4°C). A 50 μL aliquot was taken for subsequent protein analysis and this, along with the remainder of the sample, were snap frozen in liquid N2 and stored at −80°C. Protein determination was performed on the 50 μL aliquots. The remainder of each sample was then thawed and all samples normalized to the same protein concentration with lysis buffer. The lysates were then divided into 100 μL aliquots and frozen at −80°C. For in-lysate phosphatase assays, a 100 μL aliquot was thawed on ice, then an aliquot containing 4 μg of protein was diluted to 20 μL with a solution containing 2 mg/mL of BSA, 160 mM KCl, 1 mM MgCl2, 20 mM HEPES pH7.3 and 1 mM EGTA. These 20 μL aliquots of cytosol were then used directly in the phosphatase assays, see below.

Micelles consisting of a mixture of lipids and a d6-phosphoinositide substrate were prepared at RT as follows. A lipid solution containing liver PI, brain PE, brain PC, brain PS, sphingomyelin, cholesterol, brain PI(4,5)P2 and either d6-C18:0/C20:4-PI(3,4)P2 or, d6-C18:0/C20:4-PI(3,4,5)P3, was prepared in a glass vial at 32.4:23.2:10.5:23.7:2.6:0.9:2.9:3.7 (w/w) ratio (all lipids in their respective solvents) and solvents evaporated under vacuum. In order to reconstitute the lyophilized lipid mixture into micelles, 200 μL of a solution containing 20 mM HEPES pH7.3, 0.1 mM EDTA and 1 mM EGTA was added and the lipid solution bath sonicated for 1 min and divided into 20 μL aliquots.

A 20 μL aliquot of diluted cell lysate was then added to 20 μL micellar solutions and incubated at 30°C for the indicated times. Reactions were terminated by the addition of 1 mL ice-cold 20% trichloroacetic acid (TCA), followed by centrifugation (13,000 x g, 5 min, 4°C) and a single wash with 5% TCA. Samples were then incubated on ice for 5 min, centrifuged (13,000 x g, 5 min, 4°C) and the supernatant aspirated. The resultant pellet was then processed for lipid analysis as described for cell pellets, see above.

Invadopodia Assay

Slides and Cells Preparation

Prior to the experiment, 13 mm coverslips were pre-coated with gelatin enriched with fluorescently labeled Oregon Green 488 gelatin (Martin et al., 2012). Mcf10a cells were grown for 6 days in complete medium supplemented with 10 ng/mL rhTGF-β1. 5x10^4 cells were seeded in complete medium on the fluorescent gelatin surface and left to adhere (between 2-2.5 h). Next, cells were washed with PBS, starved for 4 hr, and then stimulated with 20 ng/mL hEGF for another 6 hr. Both complete and starvation media were supplemented with 10 ng/mL rhTGF-β1. After washing with PBS and fixing in 3.7% PFA (15 min at RT), cells were labeled for cortactin following a previously described protocol (Gligorijevic et al., 2014) and the nuclei (Hoechst dye, 0.8 ng/mL added to a PBS wash).

Invadopodia Imaging and Data Analysis

In each experiment, 3 coverslips were prepared per condition. Fixed and labeled cells were imaged using a confocal Nikon A1R microscope with a 60x oil objective. 15 images were obtained per coverslip with an average of 15 cells per field of view. All cells were scored for the instances in which cortactin labeling aligned with a hole created in the gelatin and these were then normalized to the number of nuclei per field of view.

Live Cell Imaging of Mcf10a Cells

Mcf10a cells expressing mCherry-PH-TAPP1 or GFP-PH-GRP1 were imaged by Spinning Disc Microscopy, z = 0.5uM at 37°C and 5% CO2, with a 100x objective.

Mouse Prostate Dissection and Processing

Mice were sacrificed using Schedule 1 methods, in agreement with the Animals (Scientific Procedures) Act 1986 (ASPA) and tissues rapidly dissected. Prostates, consisting of anterior, ventral, and dorsolateral lobes (one pair of each lobe), were dissected intact and one set of anterior, dorsolateral, and ventral lobes was used for western blot, while the other set was used for IHC. For western blot and measurements of phosphoinositides, tissues were rinsed in PBS and flash-frozen in $N_{2(l)}$. For IHC, prostates were rinsed in PBS and fixed in 4% paraformaldehyde for 1 hr at room temperature. Prostates were then cryo-protected by immersion in 30% w/v sucrose in PBS, while rotating at 4°C, for 1 hr for WT prostates, and for 2-3 hr for PTEN-KO prostates. Prostates were then immersed in embedding medium and slowly frozen on dry ice. Embedded prostates were stored at −80°C until use. 12 μm cryosections were prepared on charged glass slides using a Leica CM1850 cryostat. INPP4B-KO and PTEN-INPP4B-KO prostates were dissected intact and prepared for measurements of phosphoinositides or for immunofluorescence on site at Akita University.

Mouse Prostate Imaging

H&E Staining

H&E staining of prostate cryosections prepared on glass slides was performed using Mayer's hematoxylin and Eosin Y solutions, following a standard protocol. Images were acquired using a Zeiss Laser Microdissection microscope (20x or 40x air objectives), stitched with AxioVision4 software (5% overlap) and gaps automatically filled with Adobe Photoshop. Alternatively, an Olympus BX41 microscope equipped with a 40x oil objective was used to obtain higher resolution images. These were then manually stitched using Velocity software with a brightness correction.

Immunofluorescence

12 μm mouse prostate cryosections prepared on glass slides were stained for PI(3,4)P2 by permeabilising the sections with saponin (30 min at RT in 0.5% saponin, 1% BSA in PBS). Cells were labeled with the anti-PI(3,4)P2 antibody at 1:150 dilution for 2 hr at room

temperature, then sections were washed 3 times with PBS and incubated with streptavidin-Alexa Fluor 674 for 1 hr at room temperature, in a humid dark room. In some experiments, an additional incubation with anti-phospho-Akt-S473 antibody was carried out according to the manufacturer's instructions (Cell Signaling Technologies). After having washed the sections three times with PBS, sections were incubated in PBS supplemented with Hoechst dye for 10 min; after 3 washes with PBS, sections were then mounted with a hard-set medium. Sections were imaged with a wide field Nikon Live Cell Imager microscope and 20x air objective. Multiple fields of view were stitched automatically with 10% overlap using NIS-Elements software integrated with the microscope.

HPLC-MS Measurement of Mouse Prostate Phophoinositides

Unpublished work from our laboratories had indicated that the most predominant molecular species of PI(3,4,5)P3 and PI(3,4)P2 which accumulate in mouse prostate on deletion of PTEN were the shorter chain, more saturated species. We therefore used a new HPCL-MS method (manuscript in preparation) to analyze 17 different species of phosphoinositides in this tissue and present results for the combined total of all species measured (C32:0; C32:1; C34:0; C34:1; C34:2; C36:0; C36:1; C36:2, C36:3; C36:4; C38:3; C38:4; C38:5; C38:6; C40:4; C40;5; C40:6). Data were corrected for wet weight of tissue. At least three individual mice were used per genotype.

Mathematical Modeling

A range of mathematical models were built and analyzed in COPASI (Hoops et al., 2006). The models included the activation of EGFR by EGF, its effect on Class I PI3K activity, the phosphorylation and dephosphorylation PI(4,5)P2, PI(3,4)P2 and PI(3,4,5)P3. Reactions were modeled using mass-action or Michaelis-Menten kinetics (see Data S1, S2, and S3). PI-103 inhibition was represented as affecting the concentration of effective PI3K, while knockdown and knockout were implemented as modifications of rate constants for the corresponding enzymes. The final model was parameterized using all the available datasets, using a genetic algorithm. The complete model is provided in the COPASI format as Data S1, and in the standard format SBML (Hucka et al., 2003) as Data S2. The accession number for the mathematical model created in this manuscript is BioModels: MODEL1704190000 (Le Novère et al., 2006).

Statistics

Unless stated otherwise, data are means ± SEM of at least three biological replicates, $*p < 0.05$, $**p < 0.01$, $***p < 0.005$, and $****p < 0.0001$. For the invadopodia assay in Mcf10a clones (Figure 6D), Tukey's multiple comparisons test was used (with p values of $p < 0.05$, $p < 0.01$, $p < 0.001$ and $p < 0.0001$ corresponding to 1-4 stars on the graph).

Experimental Design

A strategy for randomization, stratification or blind selection of samples has not been carried out. Sample sizes were not chosen based on pre-specified effect size. Instead, multiple independent experiments were carried out using several sample replicates as detailed in the figure legends.

Molecular Cell, Volume *68*

Supplemental Information

PTEN Regulates PI(3,4)P_2 Signaling

Downstream of Class I PI3K

Mouhannad Malek, Anna Kielkowska, Tamara Chessa, Karen E. Anderson, David Barneda, Pınar Pir, Hiroki Nakanishi, Satoshi Eguchi, Atsushi Koizumi, Junko Sasaki, Véronique Juvin, Vladimir Y. Kiselev, Izabella Niewczas, Alexander Gray, Alexandre Valayer, Dominik Spensberger, Marine Imbert, Sergio Felisbino, Tomonori Habuchi, Soren Beinke, Sabina Cosulich, Nicolas Le Novère, Takehiko Sasaki, Jonathan Clark, Phillip T. Hawkins, and Len R. Stephens

A Calibration curve for $PI(3,4)P_2$　　B Calibration curve for $PI(4,5)P_2$

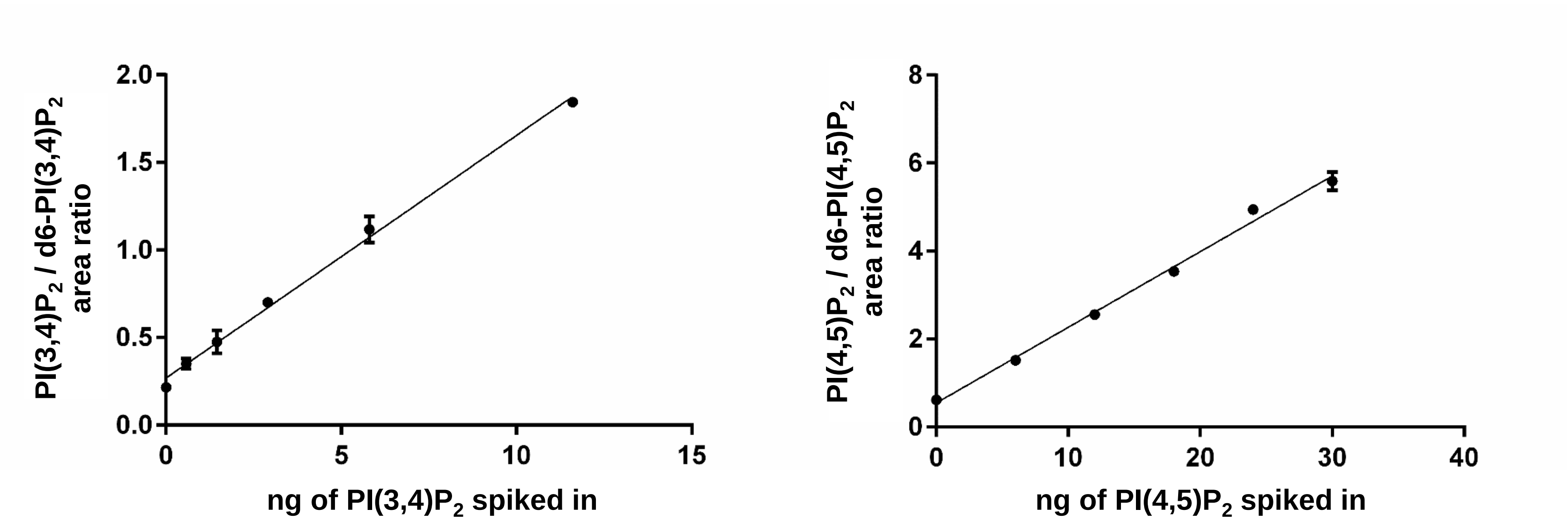

Figure S1. A HPLC-MS method for measuring $PI(3,4)P_2$ and $PI(4,5)P_2$. Related to Figure 1.
(A) Calibration curve for $PI(3,4)P_2$. **(B**) Calibration curve for $PI(4,5)P_2$. **Methods:** Mcf10a cells were incubated with 100 µM rotenone and 100 µM CCCP decoupler for 24 hrs to reduce endogenous levels of polyphosphoinositides. Cells were then killed in 1.0 M HCL and processed to form an initial homogenous lipid extraction phase, as described in Methods. The indicated quantities of synthetic C38:4 $PI(3,4)P_2$ or $PI(4,5)P_2$ and a fixed quantity of d6-C38:4 $PI(3,4)P_2$ and $PI(4,5)P_2$ were then added to portions of this extraction phase and processed by HPLC-MS. The data shown are for means +/- SD of three technical replicates.

A PI(3,4,5)P_3 levels in Mcf10a cells with altered expression of phosphatases

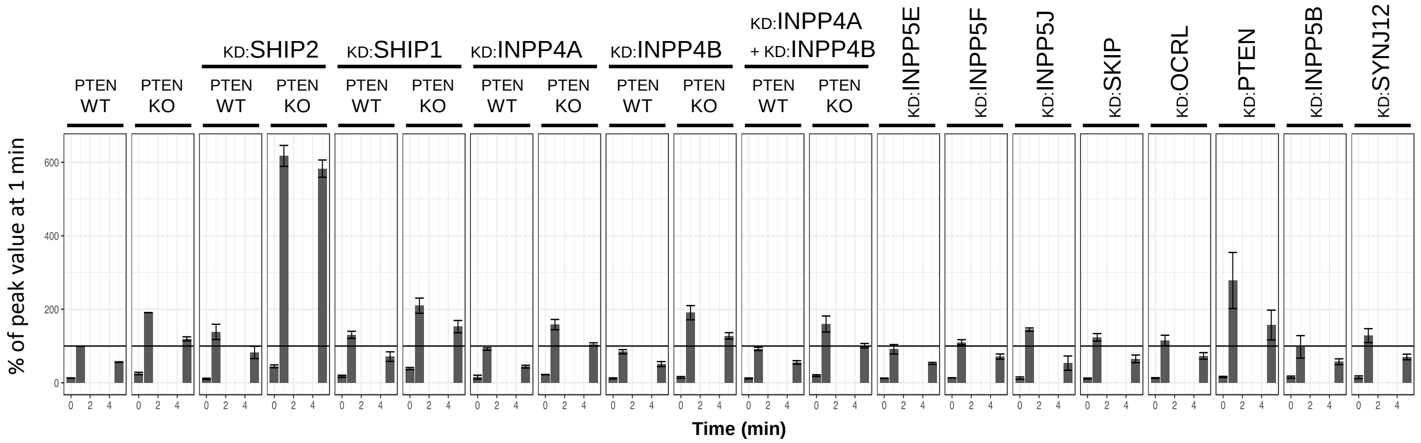

B PIP levels in Mcf10a cells with altered expression of phosphatases

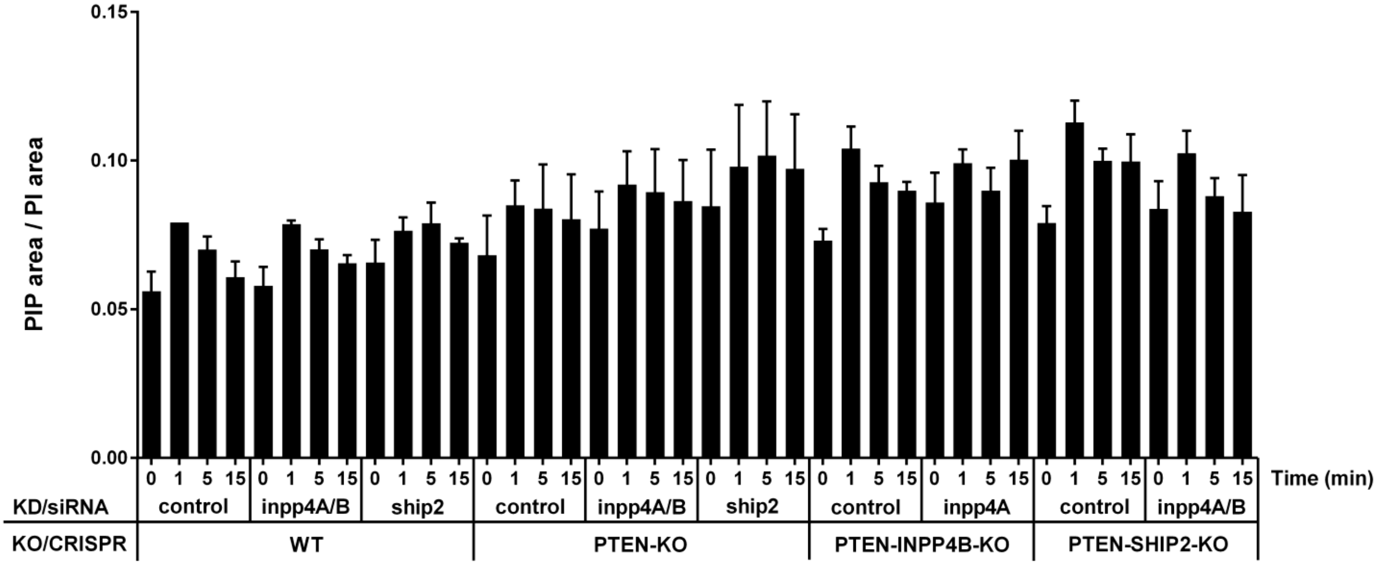

C Phosphorylation of the EGFR in genetically modified Mcf10a cells

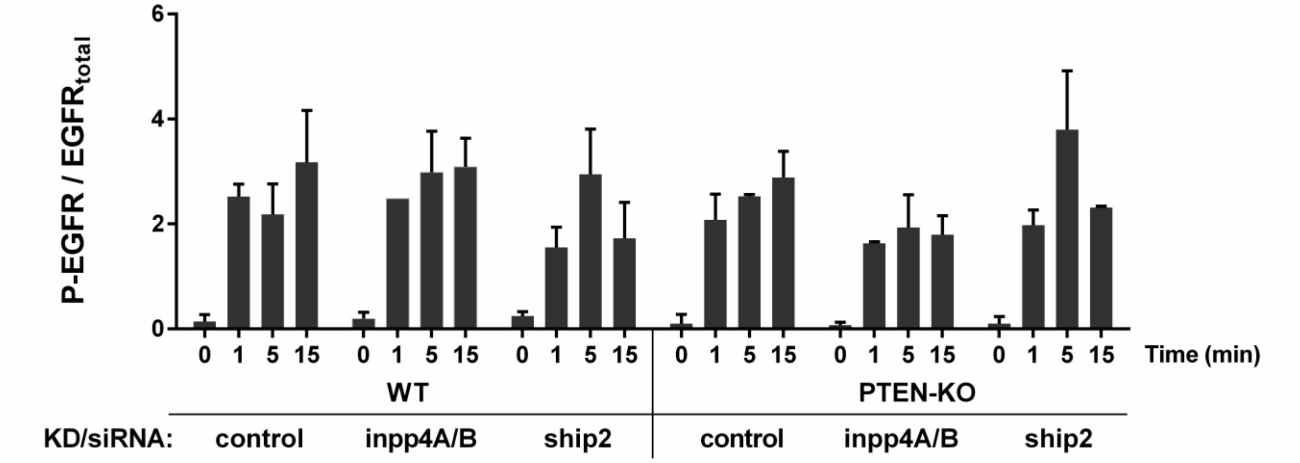

Figure S2. Characterisation of genetically modified Mcf10a cells. Related to Figure 2.

Figure S2. Characterisation of genetically modified Mcf10a cells. Related to Figure 2.

(A) $PI(3,4,5)P_3$ levels in WT or $PTEN^{-/-}$ (PTEN-KO) Mcf10a cells treated with the indicated siRNAs, starved and then stimulated with EGF (10ng/ml) for 0, 1 or 5 min. Phosphoinositides were analysed by HPLC-MS using a C4 column and $PI(3,4,5)P_3$/PI ratios calculated, as described in the Methods. For each population of cells, the data shown are normalised to the value for 1 min EGF. Data are means +/- SD of three independent experiments.

(B) PIP levels in WT or $PTEN^{-/-}$ (PTEN-KO) Mcf10a cells treated with the indicated siRNAs, starved and then stimulated with EGF (10ng/ml) for 0, 1, 5 or 15 min. Phosphoinositides were analysed by HPLC-MS using a C4 column and PIP/PI ratios calculated, as described in the Methods. Data represent means ± SD of 3 biological replicates (for siRNA suppression in WT or PTEN-KO cells) or 3 technical replicates (for PTEN-INPP4B-KO or PTEN-SHIP2-KO cells).

(C) The phosphorylation of the EGFR in Mcf10a cells treated with the indicated siRNAs, starved and then stimulated for the indicated times with EGF. The levels of EGFR and phospho-EGFR were quantified by Western blot and LiCor and represent means +/- SD of three independent experiments.

A Expression of phosphatases in cell populations treated with siRNA

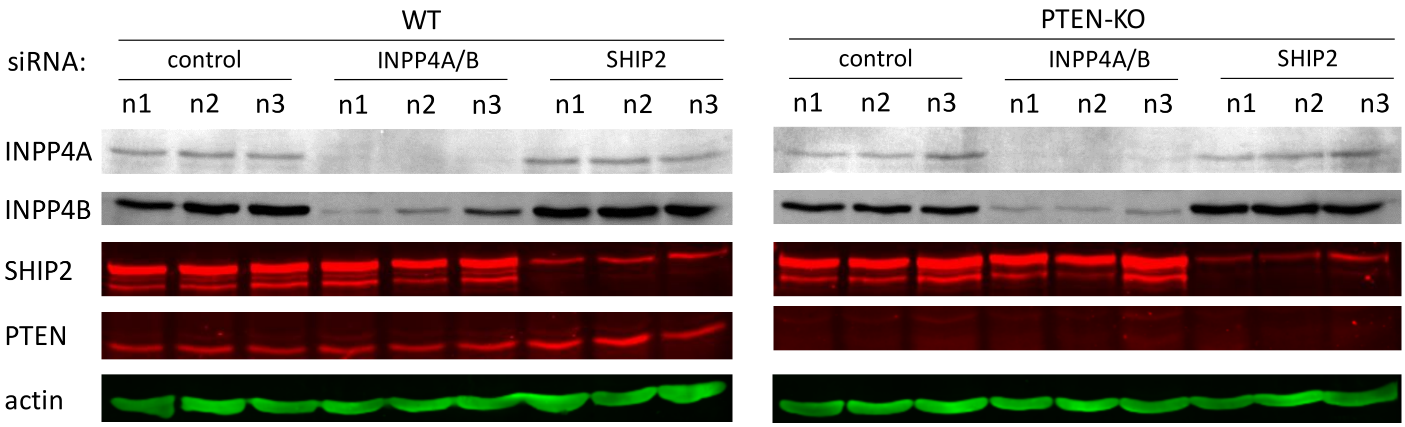

B Expression of phosphatases in clones derived by CRISPR/Cas9 gene editing

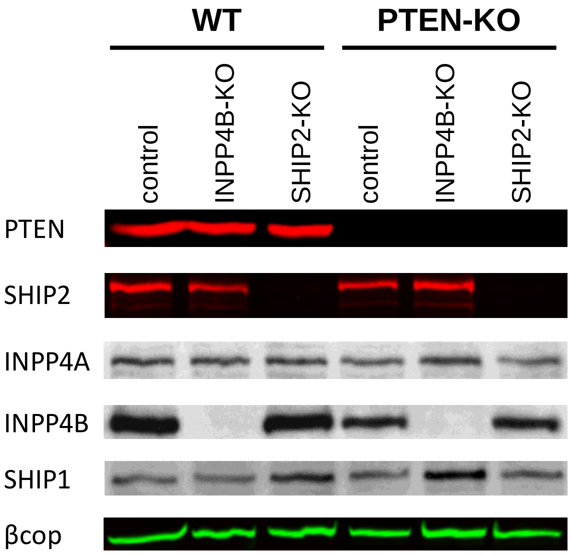

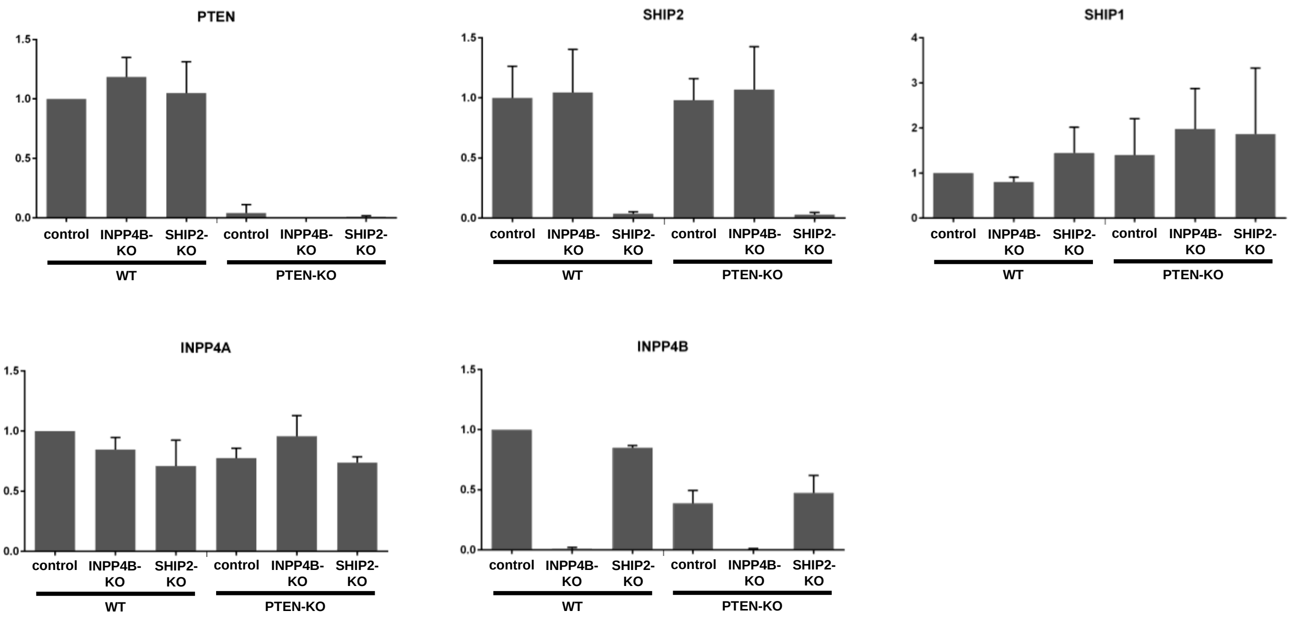

Figure S3. Expression of phosphatases in genetically modified Mcf10a cells. Related to Figure 2.

Expression of phosphatases in Mcf10a cell clones derived by siRNA gene suppression (**A**) or CRISPR/Cas9 gene editing (**B**). A representative Western blot is shown, together with the results of quantifying analogous blots using LiCor or Aida software; data are for expression levels of the indicated protein normalised to WT and are means +/- SD of three independent experiments.

A The effect of PI-103 on the accumulation of PI(3,4,5)P_3 and PI(3,4)P_2 in EGF-stimulated [INPP4A/B-KD, PTEN-KO] Mcf10a cells

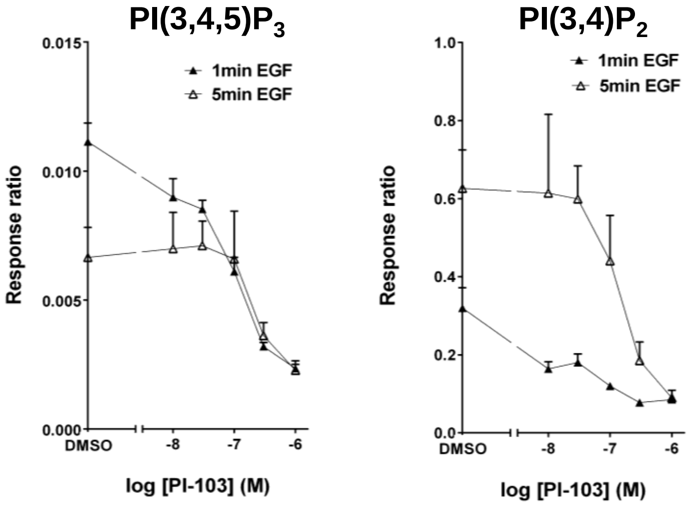

B The effect of BYL-719 on the accumulation of PI(3,4,5)P_3 and PI(3,4)P_2 in EGF-stimulated [PTEN-INPP4B-KO] Mcf10a cells

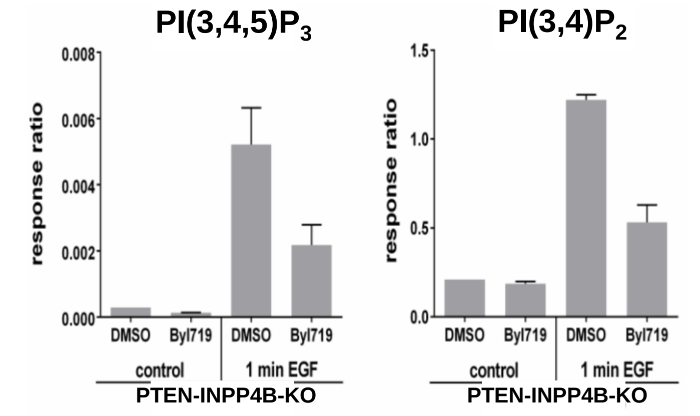

C The effect of siRNA-directed suppression of Class II PI3Kα and β on the accumulation of PI(3,4,5)P_3 and PI(3,4)P_2 in EGF-stimulated [INPP4A/B-KD, PTEN-KO] Mcf10a cells

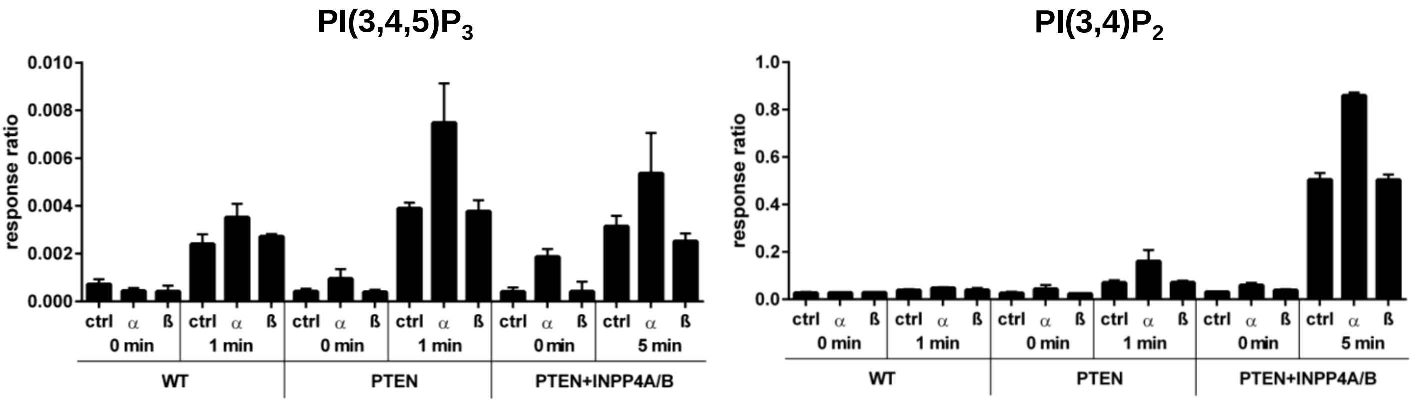

D siRNA-directed suppression of Class II PI3Kα and β in Mcf10a cells

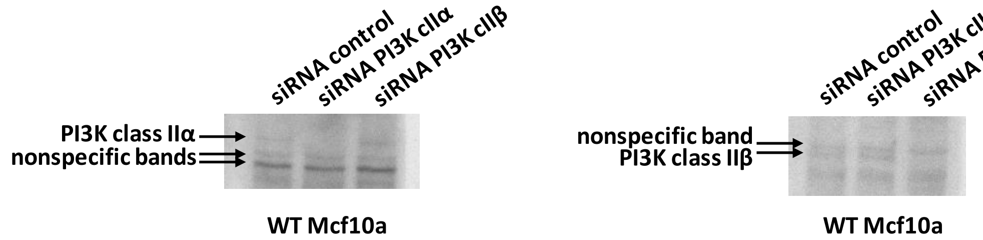

E The effect of reduced 5-phosphatase expression on EGF-stimulated PI(3,4,5)P_3 and PI(3,4)P_2 accumulation in EGF-stimulated Mcf10a cells

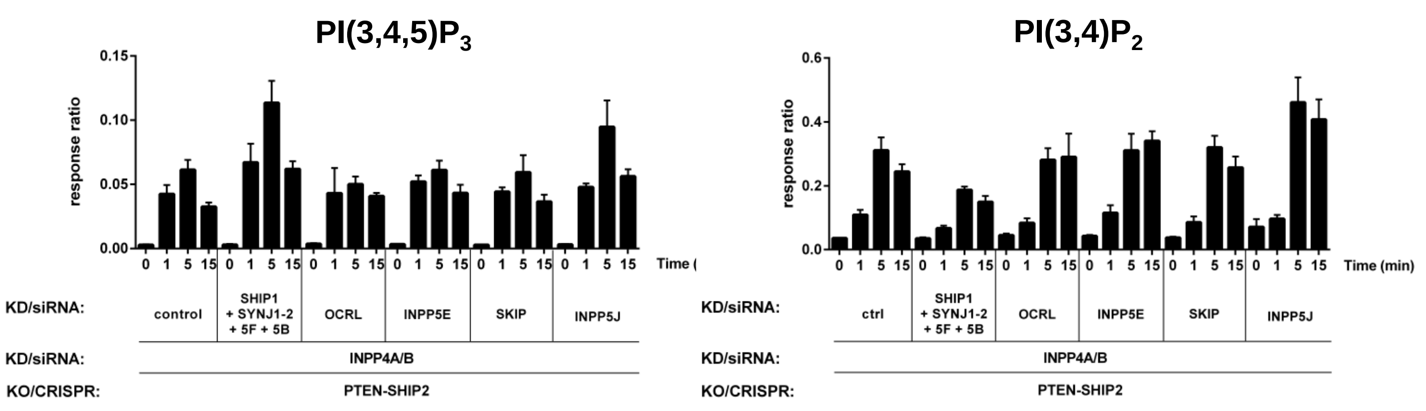

Figure S4. Evidence for Class I PI3K-driven accumulation of PI(3,4)P_2 in EGF-stimulated Mcf10a cells. Related to Figure 4.

(A) PI(3,4,5)P_3 and PI(3,4)P_2 measurements in Mcf10a cells starved, treated with the indicated concentration of PI-103 for 20 min and then stimulated with EGF (10ng/ml) for 1 min or 5 min. Data are means +/- SD of 3 technical replicates.

(B) PI(3,4,5)P_3 and PI(3,4)P_2 measurements in [PTEN-INPP4B-KO] cells starved, treated with 2μM BYL-719 or vehicle for 10 min and then stimulated with EGF (10ng/ml) or vehicle for 1 min. Data are means +/- SD of three biological replicates.

(C) PI(3,4,5)P_3 and PI(3,4)P_2 measurements in WT, PTEN-KO and [INPP4A/B-KD, PTEN-KO] cells incubated with the indicated siRNAs directed against Class II PI3Kα or β, starved, and then stimulated with EGF (10ng/ml) for 5 min. Representative data of 1 of 3 biological replicates is shown, as means +/- SD of 3 technical replicates.

(D) Representative Western blots showing expression of Class II PI3Kα or β in analogous experiments to those shown in Panel (C).

(E) PI(3,4,5)P3 and PI(3,4)P2 measurements in PTEN-SHIP2-KO Mcf10a cells treated with the indicated siRNAs and then starved and stimulated with EGF (10 ng/ml) for 0, 1, 5 or 15 min. Data are means +/- SD of 3 technical replicates.

A Comparison between HPLC-MS and ^{33}P-labelling: example HPLC traces

HPLC-MS on C18-column

HPLC on anion exchange column

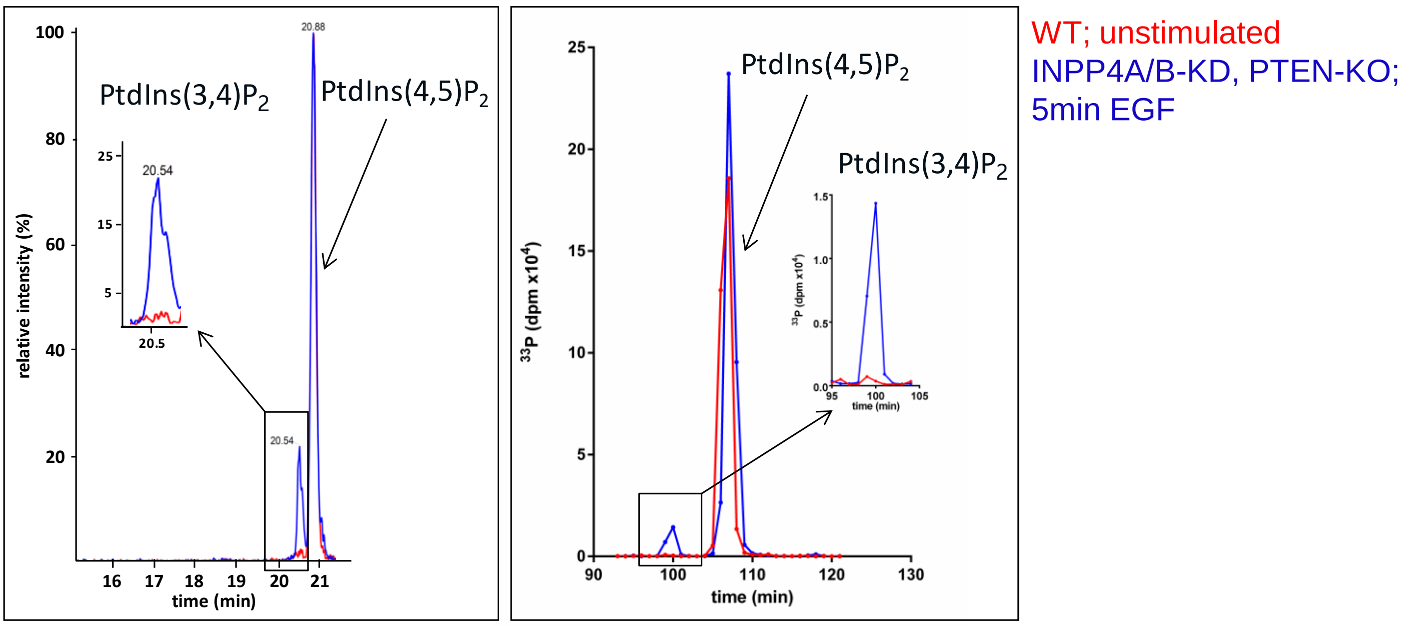

B Comparison between HPLC-MS and ^{33}P-labelling

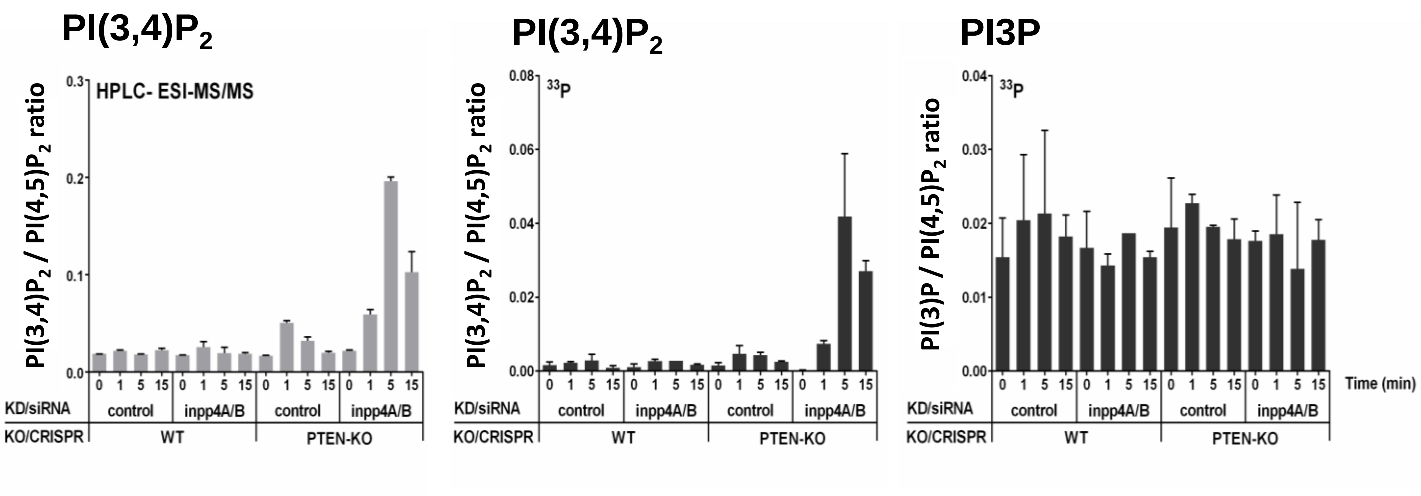

C Comparison between MS methods

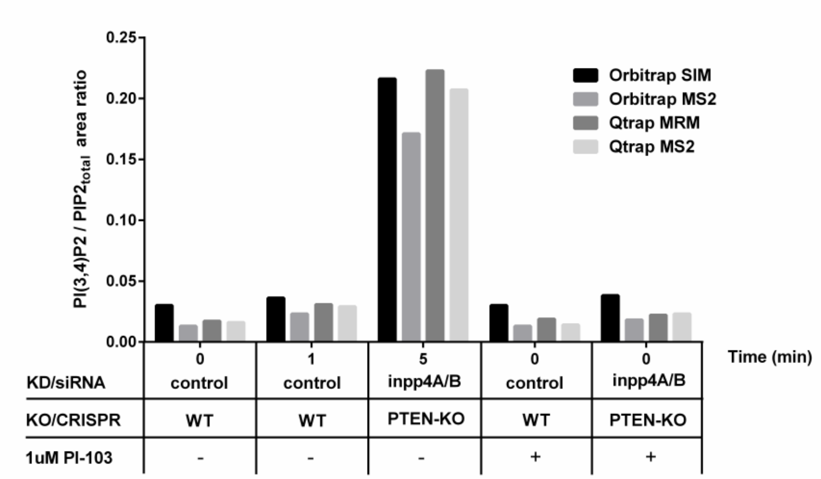

Figure S5. Comparison between different methods used to measure PI(3,4)P_2. Related to Figure 4.

(A) Example HPLC traces illustrating the raw data derived from the analysis of parallel samples of unlabelled or [^{33}-P]Pi-labelled, WT or [INPP4A/B-KD, PTEN-KO] Mcf10a cells. Cells were starved and then left unstimulated or stimulated with EGF (10ng/ml) for 5 min and analysed by HPLC-MS (left panel) or via deacylation and anion-exchange chromatography (right panel); see methods.

(B) Quantitation of traces analogous to those shown in Panel A. WT or PTEN-KO cells treated with the indicated siRNA were starved and then stimulated with EGF for 0, 1, 5 or 15 min. For the HPLC-MS values, peak areas derived from molecules corresponding to C38:4 PI(3,4)P_2 and PI(4,5)P_2 were first corrected for the recovery of internal deuterated standards before calculating a PI(3,4)P_2/PI(4,5)P_2 ratio. For the ^{33}P-labelling values, HPLC fractions corresponding to the appropriate glycerophosphoinositide species were summed, background radioactivity subtracted, and then a simple PI(3,4)P_2/PI(4,5)P_2 or PI3P/PI(4,5)P_2 ratio derived. The data shown are means +/- SD of two biological replicates.

(C) A comparison of the values obtained for PI(3,4)P_2 using identical HPLC chromatography conditions but different fragmentation strategies. WT or PTEN-KO Mcf10a cells were incubated with the indicated siRNAs, starved, treated with or without PI-103 (1 μM) for 20 min and then stimulated with EGF (10ng/ml) for 0, 1 or 5 min. Levels of PI(3,4)P_2 were measured using triple quadrupole mass spectrometer (AB SCIEX 4000 QTrap) and fragment ions were detected in multiple reaction monitoring (MRM) or MS2 mode. Parallel measurements were performed on an orbitrap mass spectrometer (Thermo Scientific Orbitrap Elite), where ions' m/z was measured with high accuracy using single ion monitoring (SIM) or MS2 approach. The values we obtained using alternative fragmentation strategies for our MS analyses reported similarly high values for PI(3,4)P_2 in both starved and PI-103-treated cells (C). A comparison between measurements for PI(3,4)P_2 obtained by HPLC-MS or [^{33}P]Pi-radiolabeling in both starved and EGF-stimulated WT, INPP4A/B-KD, PTEN-KO and [INPP4A/B-KD, PTEN-KO] cells generated a remarkably similar pattern of changes in PI(3,4)P_2/PI(4,5)P_2 ratios between conditions, but the absolute values for this ratio were higher, and the relative increases upon EGF-stimulation were lower, for the values generated by mass spectrometry (B). Note: the radiolabelling methodology allowed an estimate of PI3P levels and these are included for comparison to show they were not significantly affected by loss of PTEN (B).

Comparisons between HPLC-MS and [^{33}P]Pi-radiolabeling are difficult, because the radiolabeling approach only measures phosphoinositides that have both incorporated ^{33}P and are susceptible to methylamine-mediated deacylation, whilst our HPLC-MS method only measures intact C38:4 species. However, if the assumption is made that EGF would stimulate a similar increase in PI(3,4)P_2 measured by each method, then it must be considered plausible that contamination from non-PI(3,4)P_2 derived molecules significantly contributes to our HPLC-MS measurements for PI(3,4)P_2 in starved or PI-103-treated cells, and hence leads to an overestimation of the Class I PI3K-insensitive pool predicted by the modelling.

A Identification of PIP derived from PI(3,4)P_2 by Mcf10a cytosol as PI4P

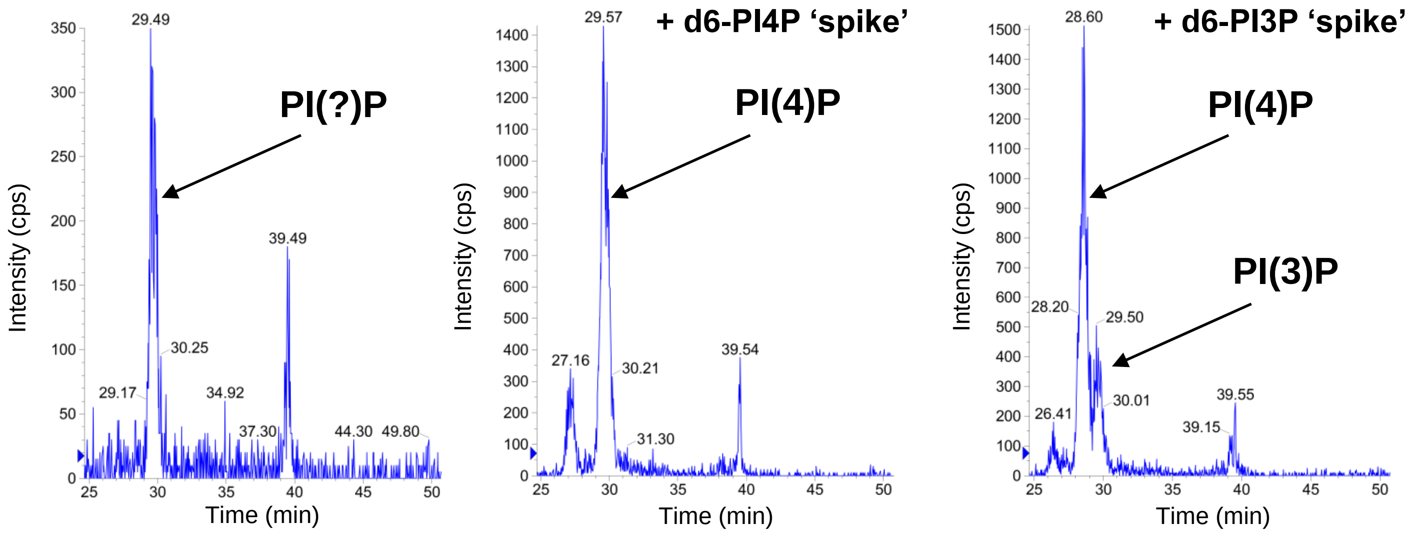

B Recombinant PTEN acts as PI(3,4)P_2 phosphatase in Mcf10a cytosol

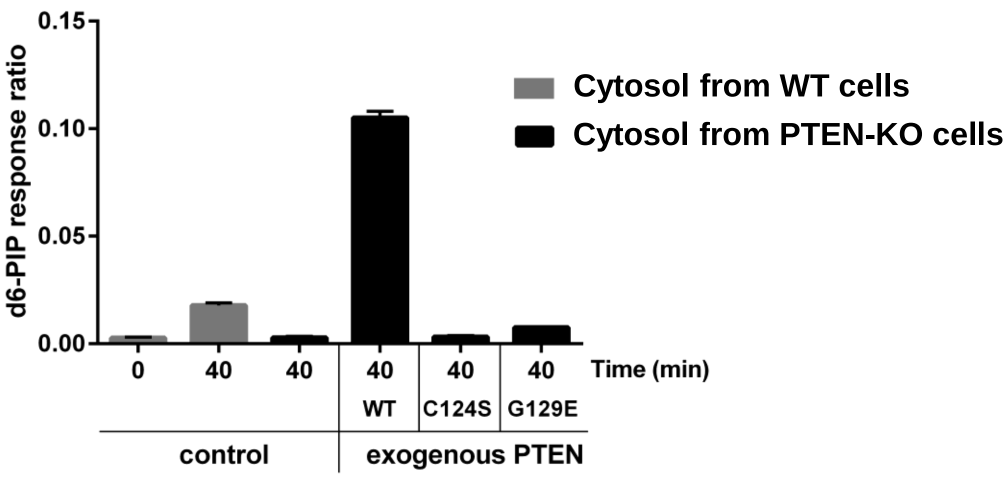

Figure S6. Dephosphorylation of PI(3,4,5)P_3 and PI(3,4)P_2 by Mcf10a cytosol. Related to Figure 5.

(A) HPLC-MS separation of the products of d6-PI(3,4)P_2 dephosphorylation by [INPP4B CRSIPR-KO] Mcf10a cytosol (left panel), together with analogous separations of samples in which additional d6-PI4P (middle) or d6-PI3P (right panel) had been added after reactions were terminated.

(B) The formation of d6-PIP by Mcf10a cytosol incubated with d6-PI(3,4)P_2. Cytosol was prepared from WT or PTEN-KO cells, and supplemented with 30nM recombinant WT or mutant PTEN, as indicated; see Methods for details.

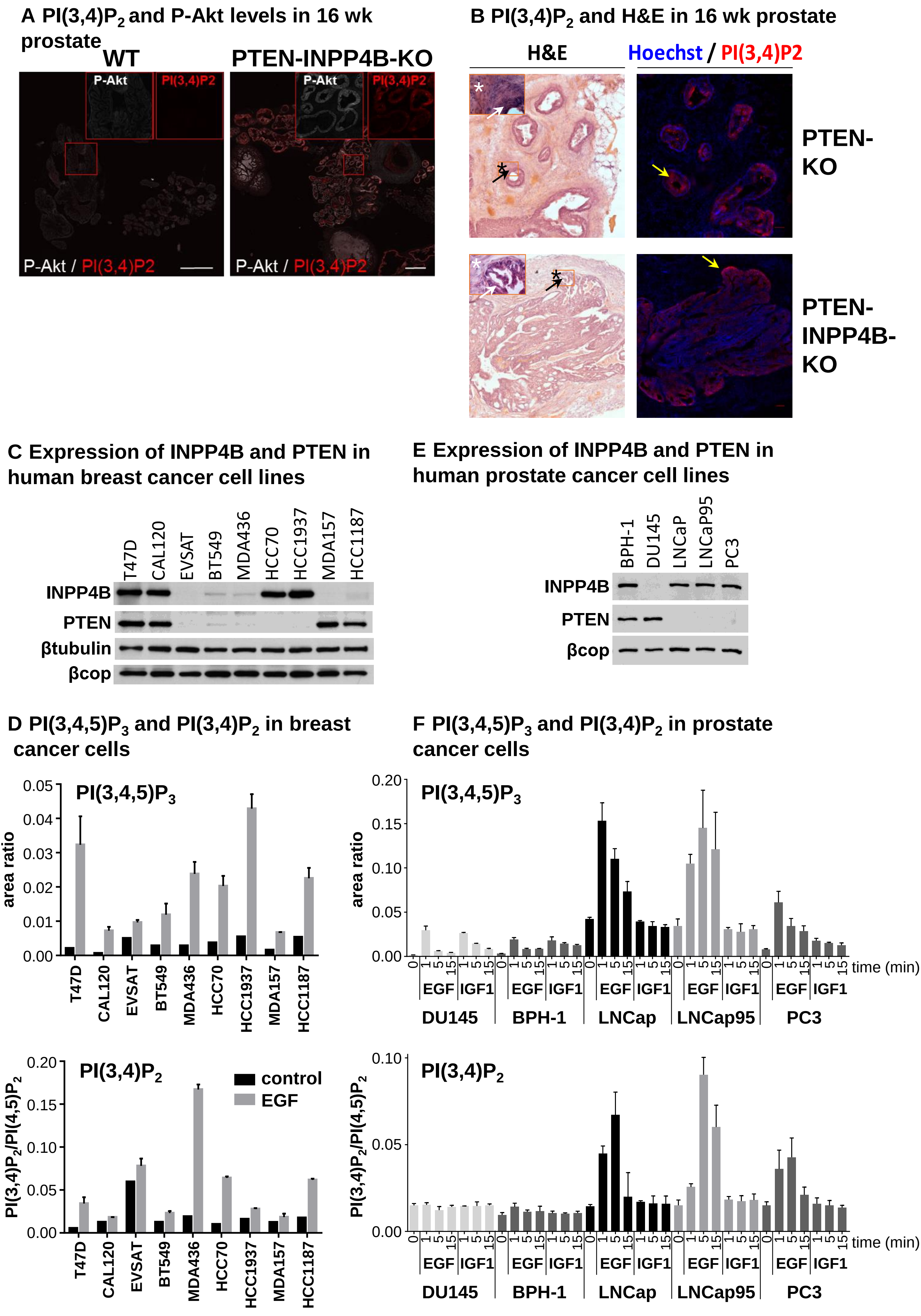

A PI(3,4)P2 and P-Akt levels in 16 wk prostate
WT
PTEN-INPP4B-KO
P-Akt
PI(3,4)P2
P-Akt / PI(3,4)P2
B PI(3,4)P2 and H&E in 16 wk prostate
H&E
Hoechst / PI(3,4)P2
PTEN-KO
PTEN-INPP4B-KO
C Expression of INPP4B and PTEN in human breast cancer cell lines
T47D
CAL120
EVSAT
BT549
MDA436
HCC70
HCC1937
MDA157
HCC1187
INPP4B
PTEN
βtubulin
βcop
E Expression of INPP4B and PTEN in human prostate cancer cell lines
BPH-1
DU145
LNCaP
LNCaP95
PC3
INPP4B
PTEN
βcop
D PI(3,4,5)P3 and PI(3,4)P2 in breast cancer cells
PI(3,4,5)P3
area ratio
0.05
0.04
0.03
0.02
0.01
0.00
PI(3,4)P2
PI(3,4)P2/PI(4,5)P2
0.20
0.15
0.10
0.05
0.00
control
EGF
F PI(3,4,5)P3 and PI(3,4)P2 in prostate cancer cells
PI(3,4,5)P3
area ratio
0.20
0.15
0.10
0.05
0.00
0 1 5 15 1 5 15
EGF IGF1
time (min)
DU145
BPH-1
LNCap
LNCap95
PC3
PI(3,4)P2
PI(3,4)P2/PI(4,5)P2
0.10
0.05
0.00

Figure S7. The impact of PTEN on PI(3,4)P_2 levels in mouse prostate and human prostate and breast cancer lines. Related to Figure 7.

(A) An example of anti-phospho-S473-AKT and anti-PI(3,4)P_2 stained sections of prostates taken from PTEN$^{flox/flox}$, PbCre$^{-/-}$ ('WT') and PTEN$^{flox/flox}$, PbCre$^{+/-}$ INPP4B$^{-/-}$ ('PTEN-INPP4B-KO') mice at 16 weeks of age (12μm sections). Some areas in anterior prostate of WT and PTEN-INPP4B-KO wide field images are shown at higher magnification, to show localisation of antibody staining. Scale bar represents 1 mm.

(B) H&E, DAPI and anti-PI(3,4)P_2 stained sections of prostates taken from PTEN$^{flox/flox}$, PbCre$^{+/-}$ ('PTEN-KO') and PTEN$^{flox/flox}$, PbCre$^{+/-}$ INPP4B$^{-/-}$ ('PTEN-INPP4B-KO') mice at 16 weeks of age (scale bar represents 69 μm). In the H&E panel and insets, arrows indicate position of hyperproliferative epithelium and asterisks indicate reactive stroma and loss of smooth muscle cell layer. In the Hoechst/PI(3,4)P_2 panel, yellow arrows indicate high PI(3,4)P_2 levels in the acini that correspond to the insets in the H&E images. The images shown are typical of 3 prostate sections analysed from 3 mice in each genotype. H&E images were stitched manually using Volocity software with brightness correction.

(C) Western blot showing relative expression of INPP4B, PTEN, βtubulin and βcop in the indicated human breast cancer cell lines (20μg total protein loaded per lane).

(D) PI(3,4,5)P_3 and PI(3,4)P_2 levels in human breast cancer cell lines starved and then stimulated with EGF (10ng/ml) for 5 min or left unstimulated. Phosphoinositides were analysed by HPLC-MS using a C4 or C18 column for measurement of PI(3,4,5)P_3 and PI(3,4)P_2, respectively. The value for the ion current area for endogenous PIP_3 was divided by the value for the ion current area of endogenous PIP_2, to correct for cell mass. The values for the ion current areas of PI(3,4P_2 and PI(4,5)P_2 were first corrected for the recovery of the appropriate internal deuterated standards and then presented as an approx molar ratio for PI(3,4P_2 divided by PI(4,5)P_2 (for details refer to the Methods section). Data are means +/- range of two biological replicates (EGF) or a single biological replicate (control).

(E) Western blot showing relative expression of INPP4B, PTEN and βcop in the indicated human prostate cancer cell lines (20μg total protein loaded per lane).

(F) PI(3,4,5)P_3 and PI(3,4)P_2 levels in human prostate cancer cell lines starved and then stimulated with EGF (10ng/ml) of IGF1 (50ng/ml) for 0, 1, 5 or 15 min. Phosphoinositides were analysed by HPLC-MS using a C4 or C18 column for measurement of PI(3,4,5)P_3 and PI(3,4)P_2, respectively. Data are presented as described for Panel (D) and are means +/- SD of at least two independent experiments.
